# Adamantylidenecarbene: Photochemical Generation, Trapping, and Theoretical Studies

**DOI:** 10.1021/acs.joc.3c01399

**Published:** 2023-09-28

**Authors:** Alexander D. Roth, Christine E. Wamsley, Sarah M. Haynes, Dasan M. Thamattoor

**Affiliations:** Department of Chemistry, Colby College, Waterville, Maine 04901, United States

## Abstract

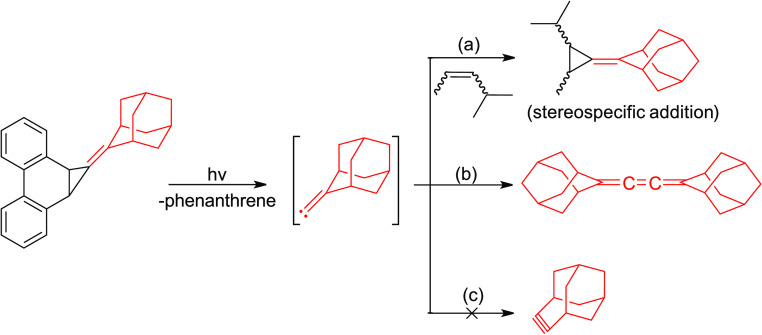

Photolysis of 1-(2-adamantylidene)-1a,9b-dihydro-1*H*-cyclopropa[*l*]phenanthrene in benzene (or benzene-*d*_6_) at ambient temperature produces adamantylidenecarbene. The carbene undergoes dimerization to a cumulene and may also be trapped in a stereospecific fashion by *cis*- and *trans*-4-methyl-2-pentene. No products attributable to 4-homoadamantyne, resulting from ring expansion of the carbene, could be detected. Coupled cluster/density functional theory calculations place the singlet carbene ∼49 kcal/mol below the triplet and show that the former must overcome a barrier of ∼13.5 kcal/mol to rearrange into 4-homoadamantyne.

## Introduction

**Scheme 1:**
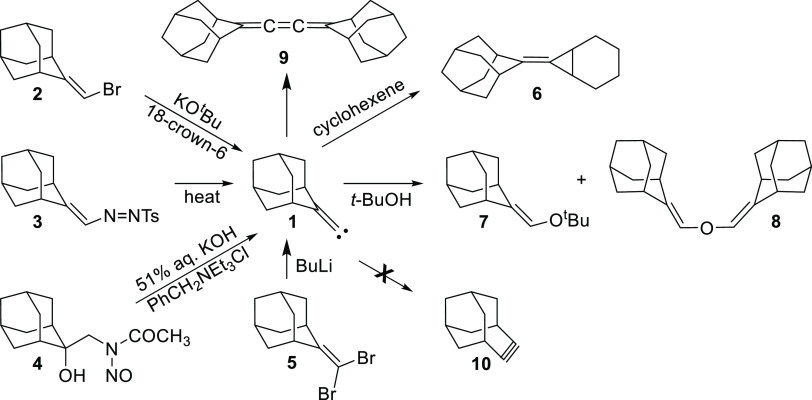
Previously Reported Thermal Routes to 2-Adamantylidenecarbene (**1**) and Its Reactions

The first reported thermal route to the caged alkylidene carbene, adamantylidenecarbene (**1**), involved heating 2-(bromomethylene)adamantane (**2**) with potassium *tert*-butoxide in various solvents using 18-crown-6 as a catalyst (Scheme 1).^1^ Subsequently, compounds **3** and **4** were identified as thermal precursors to **1** (Scheme 1), although **2** was still deemed to be superior, given its ease of synthesis, stability, and efficient conversion into the carbene.^2^ A decade later, the dibromide **5** was used as a source of **1** (or the corresponding carbenoid).^3^

**Scheme 2:**
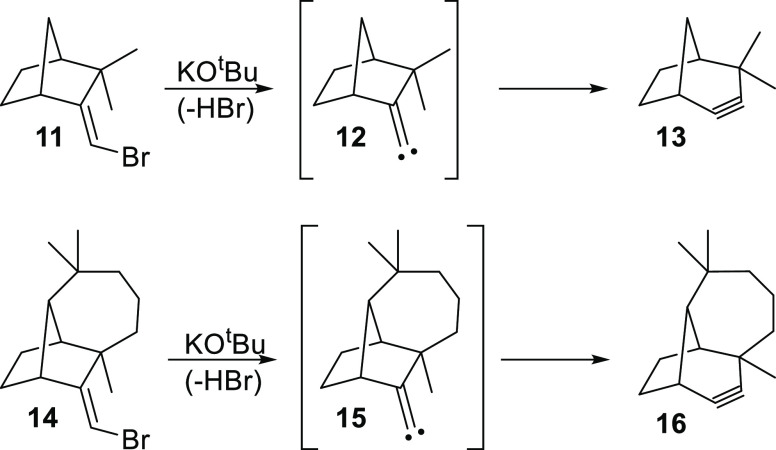
Known Ring Expansion of Vinyl Bromides **11** and **14** into the Cycloalkynes **13** and **16**, respectively

When precursors **2**–**4** were decomposed in the presence of an alkene, exemplified by cyclohexene in Scheme 1, the adduct **6** was obtained.^1,2^ Furthermore, when **2** was used as a precursor in the absence of an alkene trap, ether **7** was the major product along with minor amounts of **8**.^1,2^ Formation of **6** was rationalized as a product of the trapping of **1** by alkene, whereas **7** was attributed to the insertion of **1** into the O–H bond of *tert*-butanol that is formed as a byproduct in the reaction. The ether **8** was thought to arise by the further reaction of **7** with **1**. The cumulene **9**, presumably formed by dimerization of **1** or a surrogate carbenoid, was isolated from the reaction of **5** with butyllithium in THF at low temperatures.^3^ Conspicuously absent in all of these reactions, however, were products derived from 4-homoadamantyne (**10**) that might have resulted from the Fritsch–Buttenberg–Wiechell (FBW)^4−7^ ring expansion of **1** or its carbenoid surrogate.^1−3^ This observation stands in sharp contrast to the behavior of ω-bromocamphene (**11**)^8^ and ω-bromolongifolene (**14**)^9^ which are known to produce the corresponding cycloalkyne derivatives **13** and **16**, via the putative alkylidene carbenes **12** and **15**, respectively, upon treatment with potassium *tert*-butoxide (Scheme 2).

**Scheme 3:**
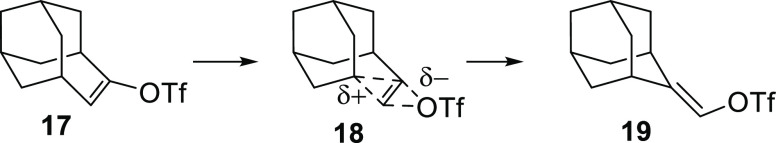
Known Ring Contraction of Triflate **17** to **19** via an Ion-Pair Intermediate. The Triflate **19**, However, Does Not Ring Expand to **17**

It was noted that nonformation of the ring-expanded product **10** was “presumably ascribable to a considerable strain increase expected in the ring enlargement” of **1** to **10** “due to the rigidity of molecular framework.”^2^ In support of this observation, it was offered^2^ that 4-homoadamanten-4-yl triflate (**17**) is known to ring contract to the adamantylidene methyl triflate (**19**) via the ion-pair intermediate **18** but **19** does not ring expand to **17** (Scheme 3).^10^ Clearly implicit in these remarks is the notion that it is **10** that would prefer to ring contract,^11^ and **10** is more strained and thus less stable than **1**.

While it is debatable whether a reasonable comparison can be made between the **17**–**18**–**19** and **2**–**1**–**10** systems, the ideas espoused in the preceding paragraph appear to be inconsistent with other experimental observations. For instance, previous work has shown that cyclohexylidenecarbene (**20)**, which is not constrained by a rigid molecular framework as **1**, does not ring expand to cycloheptyne (**21**).^12^ Likewise, later work has demonstrated that the closely related 4-methylcyclohexylidenecarbene (**22**) also fails to ring expand to **23**.^13^ In addition, Komatsu and co-workers have reported the generation of **10** by an alternative method starting from the dibromide **24**, but they do not make any mention of **10** undergoing a ring contraction.^14^ It is also important to note that as neither the **1** to **10** nor the **20 (22)** to **21 (23)** potential energy surface has received any kind of computational attention to date, theoretical insights into the nature of these rearrangements are currently unavailable in the literature.
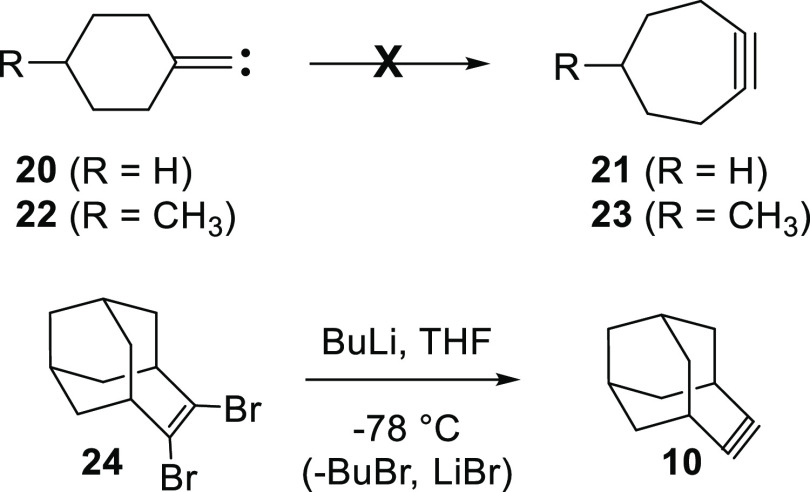


Another important question is whether **2** produces the real carbene **1** or a metal-coordinated carbenoid species. The likelihood that a true carbene was involved came from the observation that **1**, generated from either **2** or **3** as a precursor, showed comparable reactivity in competition experiments with binary mixtures of cyclohexene and another alkene.^2^ The reactivity pattern of **1** was also found to be quite close to that of isopropylidenecarbene generated from Stang’s triflate precursor.^15^

**Scheme 4:**
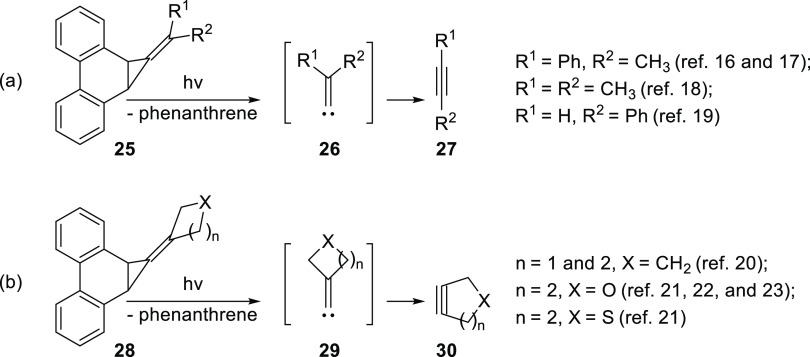
Photochemical Generation of (a) Acylic and (b) Cyclic Alkylidene Carbenes from Phenanthrene-Based Methylenecyclopropanes and Their Rearrangement into Alkynes

Our laboratory has previously demonstrated that methylenecyclopropanes appended to the 9,10 position of phenanthrene, such as **25**, can serve as viable photochemical precursors to alkylidene carbenes **26** which then rearrange into the linear alkynes **27** (Scheme 4a).^16−19^ We subsequently reported the extension of this approach to the generation of strained carbocyclic^20^ and heterocyclic^21−23^ alkynes **30** from precursors **28** via carbenes **29** (Scheme 4b). Herein, we show that a similar approach could be successfully employed as a metal-free route to produce **1** and investigate its chemistry. To the best of our knowledge, this is the first report of a photochemical generation of **1**. Detailed computational studies of the structure of **1** and the **1** to **10** potential energy surface are also presented.

## Results and Discussion

### Synthesis of a Phenanthrene-Based Photochemical Precursor to **1**

**Figure 1:**
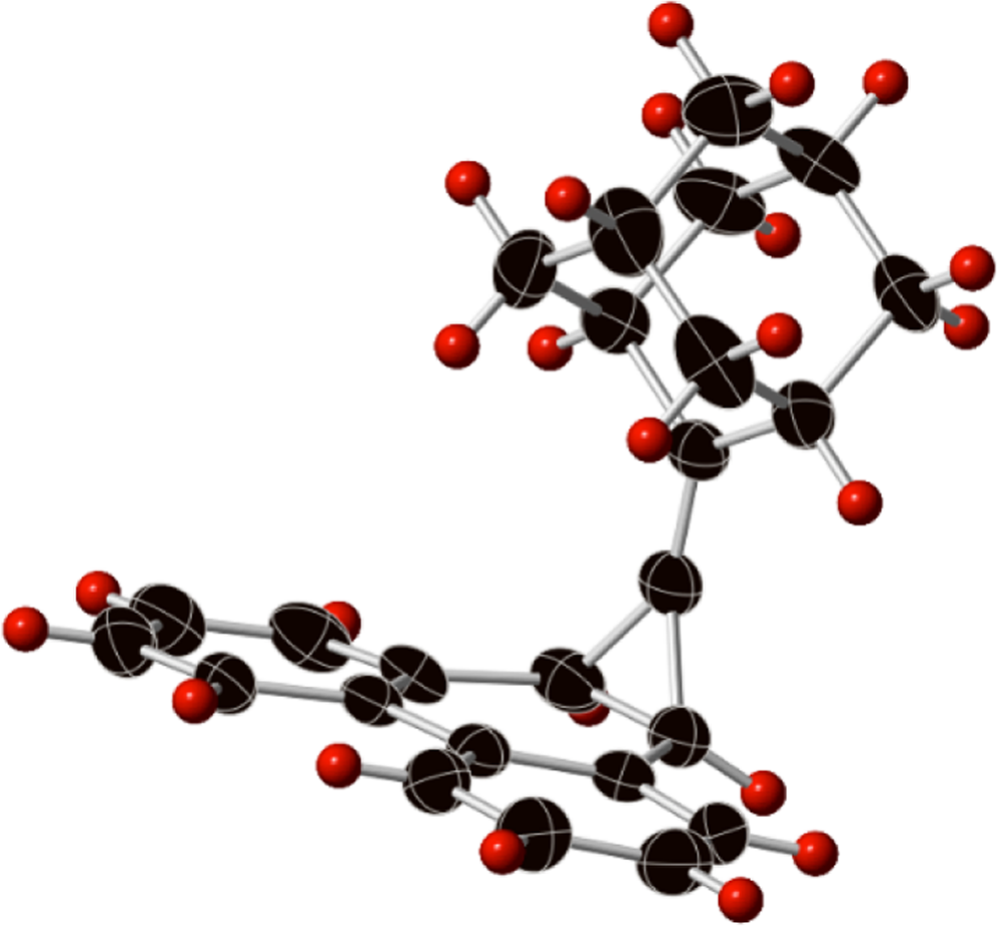
Single-crystal X-ray structure of **32**. Thermal ellipsoids are shown at the 50% probability level.

**Scheme 5:**
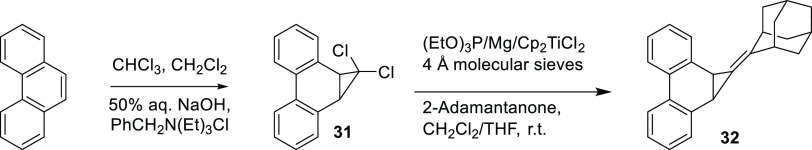
Synthesis of **32**, a Phenanthrene-Based Photochemical Precursor to **1**

Our synthesis of 1-(2-adamantylidene)-1a,9b-dihydro-1*H*-cyclopropa[*l*]phenanthrene (**32**), a photochemical precursor to **1**, was accomplished in two straightforward steps as shown in Scheme 5. The first step involved the addition of dichlorocarbene, under phase-transfer catalyzed conditions, to the 9,10 double bond of phenanthrene to form the 1,1-dichlorocyclopropane derivative **31**.^24^ In the second step, we adapted the procedure of Takeda and co-workers to convert **31** into **32** using a Cp_2_Ti[P(OEt)_3_]_2_ system.^25^ The single-crystal X-ray structure of **32** is shown in Figure 1, and its salient features are presented in the Supporting Information.

### Photochemical Experiments

Photolysis of precursor **32** was performed in several trapping agents. The alkenes *cis*- and *trans*-4-methyl-2-pentene were used as trapping agents for carbene **1**, while 1,3-diphenylisobenzofuran was used in an attempt to intercept **10**.^14^

**Scheme 6:**
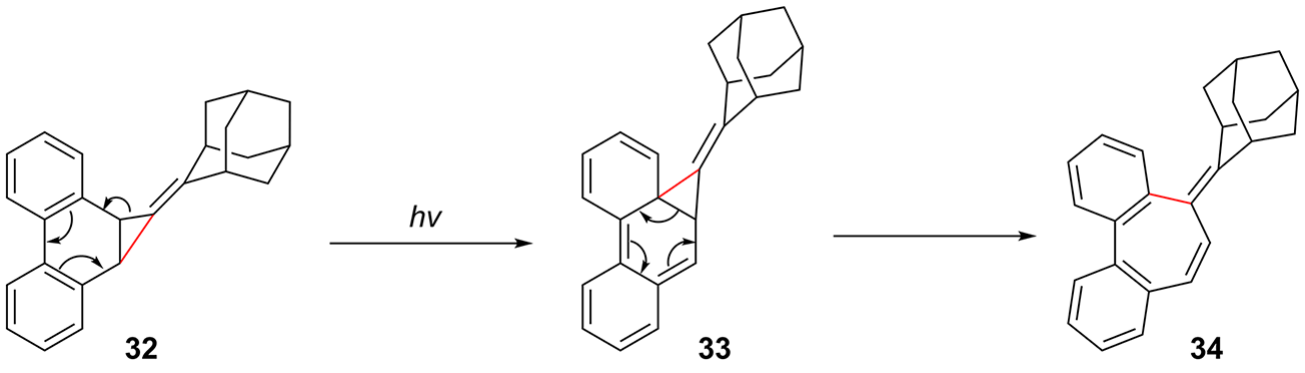
Proposed Mechanism for the Rearrangement of Precursor **32** into Isomer **34**

A competing process in all photolyses was the rearrangement of **32** to the seven-membered ring containing compound **34**. This may be attributed to the initial conversion of **32** into **33** likely via a 1,5-alkyl shift, followed by an electrocyclic ring opening of **33** to produce **34** as shown in Scheme 6. Similar rearrangements have been reported before in the indane and phenanthrene systems.^18,26^

#### Photolysis with *cis*- and *trans*-4-methyl-2-pentene

**Scheme 7:**
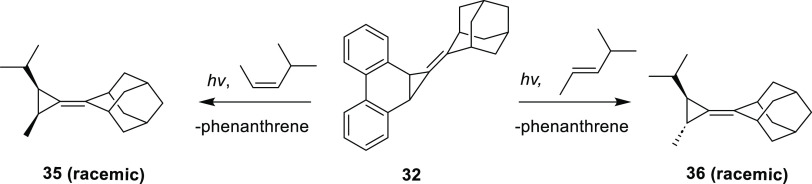
Stereospecific Trapping of Adamantylidenecarbene with *cis-* and *trans*-4-Methyl-2-pentene

**Scheme 8:**
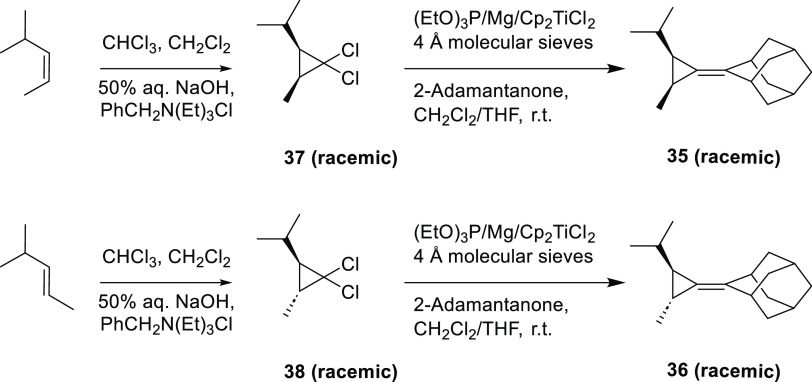
Synthesis of Authentic Samples of Adamantylidene Cyclopropanes **35** and **36**

Photolysis of **32** in *cis*- and *trans*-4-methyl-2-pentene formed the corresponding alkylidene cyclopropanes, **35** and **36**, respectively, in a stereospecific manner as shown in Scheme 7. An undetermined amount of **34** was also generated. While **35** and **36** were isolated from their respective photolysates (**35 =** 14% yield, **36** = 15% yield), the chemical shifts of the protons in the trapped adducts were too similar to accurately assign stereochemistry in the product cyclopropanes. Therefore, authentic samples of **35** and **36** were independently synthesized, as shown in Scheme 8, for comparison. The first step involved the phase-transfer catalyzed addition of dichlorocarbene to isomerically pure samples of *cis-* and *trans*-4-methyl-2-pentene to produce the corresponding dichlorocyclopropane adducts **37** and **38**, respectively. A subsequent Takeda reaction^25^ yielded **35** from **37** and **36** from **38**. The independently synthesized compounds **35** and **36** were found to be identical, per GC-MS and NMR, to the adducts formed in the photolysis of **32** with *cis-* and *trans*-4-methyl-2-pentene, respectively.

#### Photolysis with no external trapping agent

**Scheme 9:**
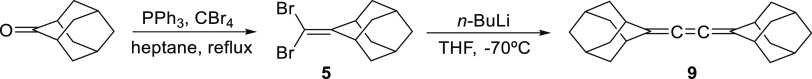
Synthesis of Cumulene **9**

Photolysis of **32** without any alkene trapping agent generated the carbene dimer, cumulene **9**, in addition to the rearranged precursor **34**. Monitoring the photolysis with NMR using 1,3-benzodioxole as an internal standard, the yields of **9** and **34** were determined to be 21 and 49%, respectively, with the remaining 30% of the photolysate consisting of material that was unable to be characterized. The identity of **9** was confirmed by preparing an authentic sample as detailed in the literature (Scheme 9)^3,27^ and comparing GC-MS and NMR data. Product **34** was isolated and characterized from a separate photolysis experiment (vide infra).

#### Photolysis with 1,3-diphenylisobenzofuran

**Scheme 10:**
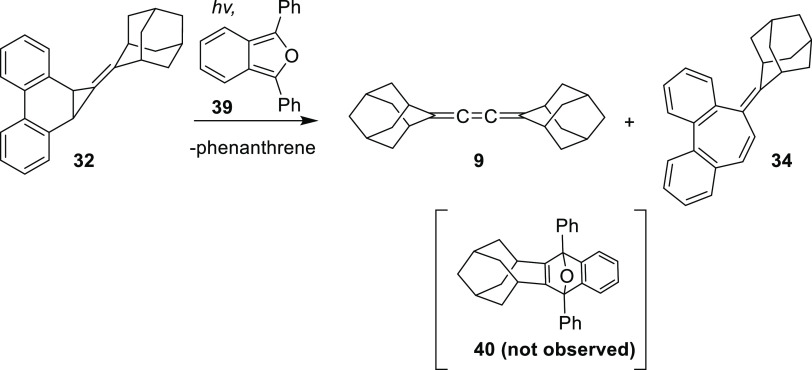
Photolysis of Precursor **32** in the Presence of Isobenzofuran (**39**) Trap

Photolysis of **32** was also performed in the presence of 1,3-diphenylisobenzofuran (**39**), a known trap for 4-homoadamantyne (**10**).^14^ However, the trapped adduct **40** expected from the reaction of **10** with **39** was not detected in the photolysate. Instead, only phenanthrene, an undetermined amount of dimer **9**, and the rearranged product **34** were observed (Scheme 10). Compound **34** was isolated from this photolysis in 35% yield.

#### Photolysis with *t*-butanol

In an attempt to determine whether **1** undergoes O–H insertion, we also performed the photolysis of **32** in an excess of *t*-butanol. In contrast to the thermal reactions reported in the literature,^1,2^ no evidence of **7** or **8** was detected, with only phenanthrene and **34** being observed.

### Computational Results

In addition to the experimental work described above, we performed quantum chemical calculations to gain further insight into the structure of **1** and its ability to ring expand to **10**. Single-point DLPNO-CCSD(T) calculations,^28−30^ performed on geometries optimized using double-hybrid density functional theory (B2PLYP)^31^ and range-separated hybrid density functional theory (ωB97x-D3BJ),^32^ placed triplet **1** at a higher energy than singlet **1** by ∼49 kcal/mol after applying zero-point vibrational energy (ZPVE) corrections (Table 1), a finding in concurrence with the literature on alkylidene carbenes.^33−35^

**Table 1:** Computed Singlet-Triplet Gaps, including ZPVE Corrections, for Adamantylidenecarbene (**1**)

computational level	Δ*E*_ST_ of **1** in kcal/mol
DLPNO-CCSD(T)/def2-TZVP//B2PLYP/def2-TZVP	–48.43
DLPNO-CCSD(T)/def2-TZVP//ωB97x-D3BJ/def2-TZVP	–49.06

**Figure 2:**
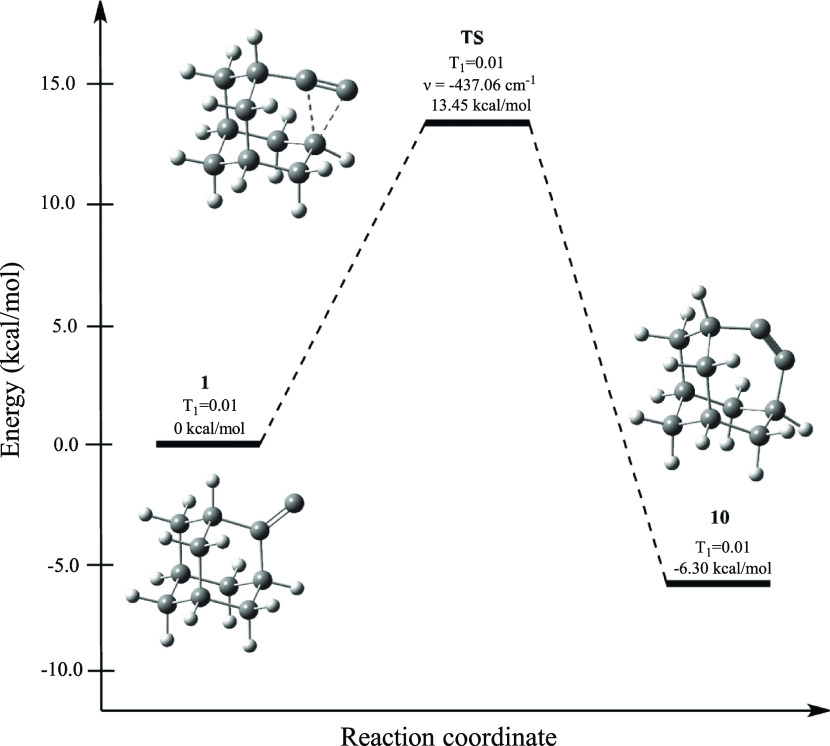
Reaction pathway for the rearrangement of singlet **1** into **10**, connected by transition state **TS**, with energies computed at the CCSD(T)/def2-TZVP//B2PLYP/def2-TZVP level. Optimized geometries, relative energies (kcal/mol), T_1_ diagnostics, and the imaginary frequency of **TS** are shown.

We then turned our attention to the potential energy surface (PES) connecting singlet **1** with the strained alkyne **10**. Geometry optimizations were initially performed at four different levels of theory (the two already mentioned above as well as with the hybrid density functional theory methods PBE0^36−38^ and B3LYP^39,40^ with def2-TZVP basis sets). Subsequently, single-point energies were calculated for each of the four optimized geometries at the DLPNO-CCSD(T)/def2-TZVP level of theory. As shown in Table 2, all four calculations unanimously place **10** at a lower energy than singlet **1** by about 6 kcal/mol, with a barrier of ∼13.5 kcal/mol separating **1** from **10**. Perhaps the severe geometric distortion of the rigid adamantyl cage required to consummate the rearrangement prevented access to **10**. Figure 2 shows a representative diagram of **1** → **TS** → **10** on the singlet PES using data from the DLPNO-CCSD(T)/def2-TZVP//B2PLYP/def2-TZVP level of theory. Similar diagrams constructed from the other three levels of theory are provided in the Supporting Information.

**Table 2:** Relative Single-Point Energies (kcal/mol), including ZPVE Corrections, for the Ring Expansion of Singlet Carbene **1** into Strained Alkyne **10**

computational level	**1** (singlet)	**TS**	**10**
DLPNO-CCSD(T)/def2-TZVP//B2PLYP/def2-TZVP	0	13.45	–6.30
DLPNO-CCSD(T)/def2-TZVP//B3LYP/def2-TZVP	0	13.45	–6.06
DLPNO-CCSD(T)/def2-TZVP//PBE0/def2-TZVP	0	13.54	–5.97
DLPNO-CCSD(T)/def2-TZVP//ωB97x-D3BJ/def2-TZVP	0	13.60	–5.84

**Figure 3:**
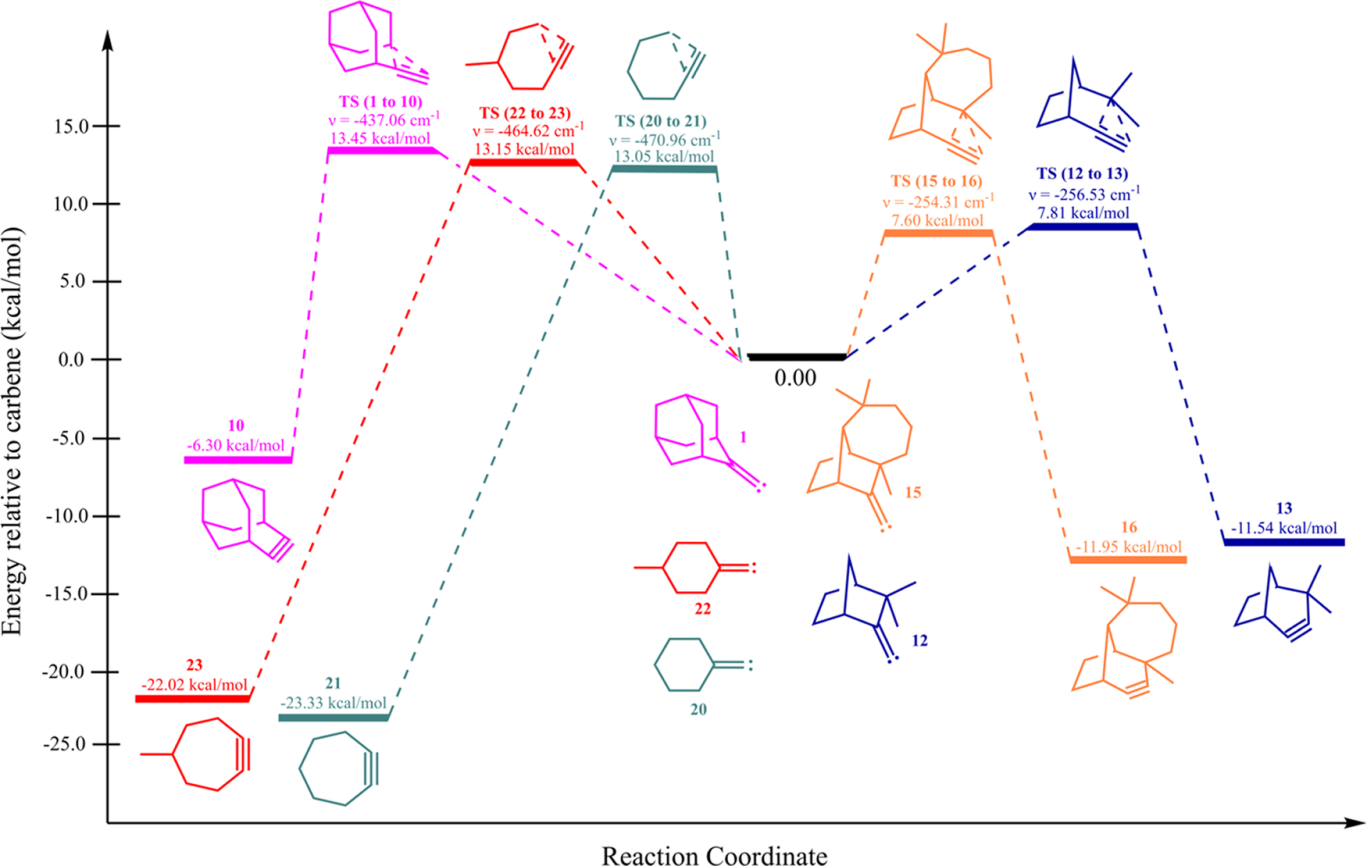
Reaction pathways for the rearrangement of various cyclic alkylidene carbenes (**1**, **12**, **15**, **20**, and **22**) into their respective alkynes, with energies computed at the CCSD(T)/def2-TZVP//B2PLYP/def2-TZVP level. Energies in kcal/mol are relative to the carbene undergoing the rearrangement. All T_1_ diagnostics are ≤0.02.

For comparison purposes, we performed additional computations at the CCSD(T)/def2-TZVP//B2PLYP/def2-TZVP level on the PES connecting **12** → **13**, **15** → **16**, **20** → **21**, and **22** → **23**. The results are collected in Table 3 and diagrammatically illustrated in Figure 3. It is noteworthy that for carbenes **1**, **20**, and **22** that do not ring expand, the barriers are all ∼13 kcal/mol and require that a six-membered ring become seven membered. For carbenes **12** and **15** that do ring expand, the barriers are significantly smaller (∼8 kcal/mol), and the process may be alternatively viewed as a five-membered ring becoming six membered. These favorable ring expansions are also consistent with our previous observations.^20−23^

**Table 3:** CCSD(T)/def2-TZVP//B2PLYP/def2-TZVP Energies (kcal/mol), including ZPVE Corrections, for the Ring Expansion of Various Cyclic Alkylidene Carbenes into the Respective Strained Alkyne^a^

reaction	carbene (singlet)	TS	alkyne
**1** to **10**	0	13.45	–6.30
**20** to **21**	0	13.05	–23.33
**22** to **23**	0	13.15	–22.02
**12** to **13**	0	7.81	–11.54
**15** to **16**	0	7.60	–11.95

a All energies are relative with respect to the carbene undergoing the rearrangement.

## Conclusions

Herein, we report the photochemical generation of adamantylidenecarbene (**1**) from the phenanthrene-based precursor 1-(2-adamantylidene)-1a,9b-dihydro-1*H*-cyclopropa[*l*] phenanthrene (**32**). We observed that the carbene dimerizes, forming a cumulene, and that it can also be trapped by *cis*- and *trans*-4-methyl-2-pentene in a stereospecific fashion to yield the corresponding cyclopropane adducts, suggesting that adamantylidenecarbene reacts as a singlet. Furthermore, we did not observe any products attributable to 4-homoadamantyne (**10**), which could have potentially arisen from the Fritsch–Buttenberg–Wiechell ring expansion of carbene **1**. Our experimental findings are consistent with results from quantum chemical calculations that indicate that singlet **1** is ∼49 kcal/mol lower in energy than the triplet and needs to overcome a barrier of ∼13.5 kcal/mol to rearrange into strained alkyne **10**. We have demonstrated, what is to our knowledge, the first example of a caged alkylidene carbene produced photochemically; thus, our phenanthrene-based method may provide an effective means of generating other such carbenes, an area of research currently being pursued in our laboratory.

## Experimental Methods

### General Experimental Procedures

Tetrahydrofuran was degassed by purging with nitrogen and dried by passage through two activated alumina columns (2 ft × 4 in). All other solvents and reagents were used as obtained from commercial sources. Unless otherwise noted, all reactions were carried out under an argon atmosphere in oven-dried glassware. The synthesis of 1,1-dichloro-1a,9b-dihydro-1H-cyclopropa[*l*]phenanthrene (**31**) was performed according to the literature.^24^ Medium-pressure flash chromatography was performed on an automated system on prepacked silica gel columns (70–230 mesh) using hexanes as an eluent. NMR spectra were recorded at 500 MHz for proton (^1^H) and 126 MHz for proton-decoupled carbon ^13^C{^1^H} using CDCl_3_. The chemical shifts are reported in δ ppm with reference to the signal of tetramethylsilane set to 0 ppm. Infrared spectra (resolution 0.4 cm^–1^) were acquired with an FTIR instrument equipped with an attenuated total reflectance (ATR) accessory and were processed with SpectraGryph.^41^ GC/MS data were obtained with a capillary gas chromatograph interfaced with a quadrupole, triple-axis mass-selective detector operating in electron impact (EI) mode. High-resolution mass spectra were obtained on a time-of-flight mass spectrometer (TOF-MS). Melting points are uncorrected.

All photolyses were performed in benzene in a long-necked quartz cuvette or in benzene-*d*_6_ in a quartz NMR tube and monitored using GC-MS and NMR, respectively. Photolyses were conducted with a medium-pressure Hg–Xe lamp (equipped with a 280–400 nm dichroic) at ambient temperature.

A Bruker D8 Quest Eco diffractometer equipped with a graphite monochromated MoKα radiation (λ= 0.71073 Å) and PHOTON 50 CMOS (**c**omplementary **m**etal-**o**xide **s**emiconductor) detector was used to collect X-ray diffraction data at 173 K with the Bruker Apex 3 suite of programs.^42^ Frames were integrated with a narrow-frame algorithm using the Bruker data reduction software package SAINT+^43^ and absorption effects were corrected with the multiscan method (SADABS).^44^ The Olex2 suite of programs^45^ was used to process data along with the Bruker SHELXTL software package^46,47^ that was used to perform structure solution by direct methods and refinement by full-matrix least-squares on F^2^. All non-hydrogen atoms were refined anisotropically with suggested weighting factors, and the hydrogens were calculated on a riding model. All cif files were validated with the checkCIF/Platon facility of IUCr that was implemented through Olex2.^45^

The quantum chemistry program Orca (version 5.0) was used to perform all calculations.^48^ Geometry optimization calculations were performed using double-hybrid density functional theory (B2PLYP),^31^ hybrid density functional theory (PBE0^36−38^ and B3LYP^39,40^), and range-separated hybrid functional theory (ωB97x-D3BJ)^32^ methods. All single-point calculations utilized domain-based local pair natural orbital-coupled cluster [DLPNO-CCSD(T)] methods.^28−30^ Calculations were universally performed in combination with Ehrlich’s def2-TZVP^49^ using the auxiliary basis sets def2/J^50^ and def2-TZVP/C.^51,52^ The resolution-of-identity option^53^ and the chain-of-spheres^54,55^ algorithm (RIJCOSX) were employed to accelerate SCF and exchange integral calculations, respectively. All calculations used Grimme’s atom pairwise dispersion correction with Becke–Johnson damping (D3BJ).^38,56^ Frequency calculations were performed to verify the stationary points as minima (0 imaginary frequency) or maxima (1 imaginary frequency). T1 diagnostic^57^ values for all CCSD(T) calculations were ≤0.02, suggesting the species had negligible multireference character and were sufficiently represented by the wave functions. SCF stability analysis for all wave functions indicated stable HF/KS wave functions with negligible triplet contamination. ChemCraft^58^ was used to visualize computational data.

#### 1-(2-Adamantylidene)-1a,9b-dihydro-1H-cyclopropa[*l*]phenanthrene (**32**)

To a 100 mL three-neck flask were added 4 Å molecular sieves (0.717 g), crushed magnesium turnings (0.174 g, 7.16 mmol), and a stir bar, all of which were dried in an oven overnight. The flask was then cooled to r.t. under Ar, and titanocene dichloride (1.824 g, 7.33 mmol), P(OEt_3_) (2.5 mL, 2.42 g, 14.6 mmol), and THF (15 mL) were added. The reaction mixture was allowed to stir for 3 h or until the magnesium reacted fully. Then, **31** (0.716 g, 2.75 mmol) in THF was added all at once to the black reaction mixture. The reaction mixture remained black and was stirred for 40 min, at which point 2-adamantanone (0.210 g, 1.40 mmol) in THF was added. The reaction mixture was stirred overnight, and it was subsequently filtered through a plug of Celite. The Celite was washed multiple times with DCM (3 × 40 mL), and the resultant organic filtrate was washed with H_2_O (2 × 20 mL) and brine (3 × 40 mL) and dried with sodium sulfate. The crude product was purified first by flash column chromatography on silica gel using hexanes and then via manual column on silica treated with 25% w/w AgNO_3_ using hexanes/ethyl acetate to obtain **32** as a white solid. Single crystals suitable for X-ray crystallography were obtained by slow evaporation of the hexanes from the column fractions. Yield: 0.137 g (31%); mp: 140–142 °C. ^1^H NMR (500 MHz, CDCl_3_): δ 8.00–7.87 (m, 2H), 7.40–7.34 (m, 2H), 7.25–7.17 (m, 4H), 3.17 (s, 2H), 2.56 (t, *J* = 3.2 Hz, 2H), 1.94 (p, *J* = 3.2 Hz, 1H), 1.83 (t, *J* = 2.9 Hz, 4H), 1.69 (d, *J* = 3.2 Hz, 2H), 1.63 (p, *J* = 3.2 Hz, 1H), 1.56–1.47 (m, 2H), 1.06–0.93 (m, 2H).^13^C {^1^H} NMR (126 MHz, CDCl_3_): 138.5, 134.8, 129.1, 128.6, 127.5, 125.5, 123.2, 112.5, 38.9, 38.6, 37.2, 36.3, 28.2, 28.2, 21.8. FTIR: ν 3065, 2904.4, 2844.1, 1497.8, 1484.5, 1464.5, 1444.5 cm^–1^. HRMS (ESI): [M + H]^+^ calcd for C_25_H_25_: 325.1956; found: 325.1965.

#### 2-(5H-Dibenzo[*a*,*c*][7]annulen-5-ylidene)adamantane (**34**)

Isolated from photolysis D (vide infra). Yield: 17 mg (35%). ^1^H NMR (500 MHz, CDCl_3_): δ 7.66–7.62 (m, 1H), 7.55–7.51 (m, 1H), 7.35–7.28 (m, 4H), 7.26–7.24 (m, 1H), 7.16–7.11 (m, 1H), 6.66 (d, *J* = 10.9 Hz, 1H), 6.50 (d, *J* = 11.0 Hz, 1H), 2.94 (s, 1H), 2.75 (s, 1H), 2.06–1.71 (m, 8H), 1.58 (dq, *J* = 12.2, 2.7 Hz, 1H), 1.43 (dd, *J* = 10.0, 5.1 Hz, 1H), 1.27 (dd, *J* = 15.5, 3.1 Hz, 2H). ^13^C {^1^H} NMR (126 MHz, CDCl_3_): δ 146.8, 143.1, 139.7, 138.5, 136.3, 134.4, 130.4, 129.7, 129.0, 128.3, 128.2, 127.1, 126.6, 126.4, 126.3, 124.2, 39.6, 39.4, 39.2, 38.6, 37.0, 33.6, 33.1, 28.4, 28.0. FTIR: ν 3057.9, 3012.5, 2901.6, 2845.4, 1481, 1465.8, 1446.9, 1436.9 cm^–1^. HRMS (ESI): [M + H]^+^ calcd for C_25_H_25_: 325.1956; found: 325.1971.

#### *cis*-1,1-Dichloro-2-isopropyl-3-methylcyclopropane (**37**)

Reported previously in the literature,^59^ but synthesized according to our own procedure. To a 50 mL Erlenmeyer under ambient atmosphere with vigorous stirring were added *cis*-4-methyl-2-pentene (0.625 mL, 0.419 g, 4.99 mmol), benzyltriethylammonium chloride (58.7 mg, 0.255 mmol), and chloroform (10 mL). The reaction mixture was cooled to ∼5 °C, where 4 mL of 50% NaOH (aq) was slowly added. The reaction was allowed to stir for 30 min at 5 °C, then it was warmed to room temperature and allowed to react overnight. The layers were separated, the aqueous layer was extracted with CH_2_Cl_2_ (2 × 20 mL), and the organic layer was washed with brine (1 × 20 mL) and dried with sodium sulfate. The crude product was purified by flash column chromatography on silica gel using hexanes as an eluent to obtain **37** as a light yellow oil. Yield: 0.632 g (76.1%). ^1^H NMR (500 MHz, CDCl_3_): δ 1.62 (dq, *J* = 11.1, 6.6 Hz, 1H), 1.49 (dp, *J* = 10.8, 6.6 Hz, 1H), 1.25 (t, *J* = 11.0 Hz, 1H), 1.14 (d, *J* = 6.6 Hz, 3H), 1.12 (d, *J* = 6.5 Hz, 3H), 0.96 (d, *J* = 6.7 Hz, 3H). ^13^C {^1^H} NMR (126 MHz, CDCl_3_): δ 65.7, 39.8, 27.4, 25.5, 21.6, 21.3, 9.0. FTIR: ν 2961.4, 2934.5, 2872.5, 1466.1 cm^–1^.

#### *cis*-2-(2-Isopropyl-3-methylcyclopropylidene)adamantane (**35**)

To a 100 mL three-necked flask were added 4 Å molecular sieves (0.717 g), crushed magnesium turnings (0.174 g, 7.16 mmol), and a stir bar, all of which were dried in an oven for 3 h. The flask was then cooled to r.t. under Ar, and titanocene dichloride (1.836 g, 7.40 mmol), P(OEt_3_) (2.5 mL, 2.42 g, 14.6 mmol), and THF (15 mL) were added. The mixture was allowed to stir for 3 h or until the magnesium reacted fully. Then, **37** (0.298 g, 1.80 mmol) in THF was added at once to the black reaction mixture. The mixture was stirred for 40 min, at which point an excess of 2-adamantanone (0.512 g, 3.41 mmol) in THF was added. The reaction mixture was allowed to stir for 2 more hours, and it was subsequently filtered through a plug of Celite. The Celite was washed multiple times with DCM (3 × 20 mL), and the resultant organic filtrate was washed with water (2 × 20 mL) and brine (3 × 40 mL) and dried with sodium sulfate. The crude product was purified by flash column chromatography on silica gel using hexanes as an eluent, yielding **35** as a viscous colorless oil. Yield: 0.077 g (37.2%). ^1^H NMR (500 MHz, CDCl_3_): δ 2.70 (q, *J* = 3.1 Hz, 1H), 2.61 (dt, *J* = 5.3, 2.7 Hz, 1H), 1.98–1.94 (m, 2H), 1.91 (dt, *J* = 12.3, 2.7 Hz, 2H), 1.88–1.82 (m, 4H), 1.82–1.76 (m, 4H), 1.40 (dq, *J* = 8.8, 6.3 Hz, 1H), 1.31 (dp, *J* = 9.9, 6.6 Hz, 1H), 1.20 (dd, *J* = 9.9, 8.8 Hz, 1H), 1.07 (d, *J* = 6.3 Hz, 3H), 1.03 (d, *J* = 6.6 Hz, 3H), 0.99 (d, *J* = 6.6 Hz, 3H). ^13^C {^1^H} NMR (126 MHz, CDCl_3_): δ 135.8, 118.1, 40.2, 39.9, 39.0, 38.8, 37.5, 37.1, 37.1, 28.8, 28.4, 28.1, 27.2, 24.0, 23.1, 12.1 (two overlapping carbon resonances). FTIR: ν 2950.5, 2905.6, 2865.7, 2847.1, 1464.9, 1446.9 cm^–1^. HRMS (ESI): [M + H]^+^ calcd for C_17_H_27_: 231.2113; found: 231.2096.

#### *trans*-1,1-Dichloro-2-isopropyl-3-methylcyclopropane (**38**)

Reported previously in the literature but synthesized according to our own procedure. To a 50 mL Erlenmeyer under ambient atmosphere with vigorous stirring were added *trans*-4-methyl-2-pentene (0.610 mL, 0.418 g, 4.99 mmol), benzyltriethylammonium chloride (58.7 mg, 0.255 mmol), and chloroform (10 mL). The reaction mixture was cooled to ∼5 °C, and 8 mL of 50% NaOH (aq) was slowly added. The reaction was allowed to stir for 30 min at 5 °C, then it was warmed to r.t. and allowed to react overnight. The layers were separated, the aqueous layer was extracted with CH_2_Cl_2_ (3 × 20 mL), and the organic layer was washed with brine (1 × 20 mL) and dried with sodium sulfate. The crude product was purified by flash column chromatography on silica gel using hexanes as an eluent to obtain **38** as a light yellow oil. Yield: 0.549 g (65.9%). ^1^H NMR (500 MHz, CDCl_3_): δ 1.41 (ddt, *J* = 13.3, 10.0, 6.7 Hz, 1H), 1.26 (d, *J* = 6.0 Hz, 3H), 1.22–1.16 (m, 1H), 1.12 (d, *J* = 6.6 Hz, 3H), 1.01 (d, *J* = 6.8 Hz, 3H), 0.81 (dd, *J* = 10.1, 7.8 Hz, 1H). ^13^C {^1^H} NMR (126 MHz, CDCl_3_): δ 67.1, 44.4, 31.0, 30.3, 21.8, 22.2, 15.0. FTIR: ν 2960.1, 2898.1, 2872.7, 1464.7, 1415.5 cm^–1^.

#### *trans*-2-(2-Isopropyl-3-methylcyclopropylidene)adamantane (**36**)

To a 100 mL three-necked flask were added 4 Å molecular sieves (0.717 g), crushed magnesium turnings (0.174 g, 7.16 mmol), and a stir bar, all of which were dried in an oven for 3 h. The flask was then cooled to r.t. under Ar, and titanocene dichloride (1.836 g, 7.40 mmol), P(OEt_3_) (2.5 mL, 2.42 g, 14.6 mmol), and THF (15 mL) were added. The mixture was allowed to stir for 3 h or until the magnesium reacted fully. Then, **38** (0.279 g, 1.68 mmol) in THF was added at once to the black reaction mixture. The mixture was stirred for 40 min, at which point an excess of 2-adamantanone (0.522 g, 3.48 mmol) in THF was added. The reaction mixture was allowed to stir for 2 more hours, and it was subsequently filtered through a plug of Celite. The Celite was washed multiple times with DCM (3 × 20 mL), and the resultant organic filtrate was washed with water (2 × 20 mL) and brine (3 × 40 mL) and dried with sodium sulfate. The crude product was purified by flash column chromatography on silica gel using hexanes as an eluent, yielding **36** as a viscous colorless oil. Yield: 0.041 g (21.2%). ^1^H NMR (500 MHz, CDCl_3_): δ 2.64 (t, *J* = 3.2 Hz, 1H), 2.60 (t, *J* = 3.2 Hz, 1H), 1.96 (t, *J* = 3.2 Hz, 2H), 1.88 (dt, *J* = 12.8, 2.9 Hz, 3H), 1.84–1.82 (m, 2H), 1.81–1.75 (m, 5H), 1.30 (dp, *J* = 7.8, 6.7 Hz, 1H), 1.08 (d, *J* = 5.7 Hz, 3H), 1.04–1.00 (m, 1H), 0.95 (d, *J* = 6.7 Hz, 3H), 0.92 (d, *J* = 6.7 Hz, 3H), 0.82 (dd, *J* = 7.7, 3.5 Hz, 1H). ^13^C {^1^H} NMR (126 MHz, CDCl_3_): δ 136.1, 118.8, 39.6, 39.5, 39.4, 39.4, 37.5, 37.3, 31.8, 31.3, 29.7, 28.7, 28.6, 22.5, 22.3, 18.0, 14.1. FTIR: ν 2952.5, 2906.8, 2848.2, 1465, 1447.2 cm^–1^. HRMS (ESI): [M + H]^+^ calcd for C_17_H_27_: 231.2113; found: 231.2124.

### Procedures for Photolysis

#### Photolysis with *cis*-4-Methyl-2-pentene

The adamantylidenecarbene precursor **32** (83 mg, 0.26 mmol) was dissolved in 1 mL of benzene in a long-necked quartz cuvette. One milliliter of *cis*-4-methyl-2-pentene was added thereafter to serve as a trapping agent. Photolysis was carried out under the HgXe lamp, and the progress of the reaction was monitored via GC-MS. The photolysis lasted for 3 h, or until all **32** had either photolyzed or rearranged into **34**. The crude product was purified by flash column chromatography on silica gel using hexanes as an eluent, yielding exclusively the *cis*-cyclopropylideneadamantane **35** as a viscous clear oil. Yield: 8 mg (13.6%).

#### Photolysis with *trans*-4-Methyl-2-pentene

Precursor **32** (92 mg, 0.28 mmol) and *trans*-4-methyl-2-pentene (0.6 mL) were dissolved in 1 mL of benzene in a long-necked quartz cuvette. Photolysis was monitored via GC-MS until all **32** had reacted or 1.5 h. While a minor amount of the *cis* product was formed due to impurities within our sample of trapping agent, the *trans* cyclopropylideneadamantane was the major product. The crude product was purified by flash column chromatography on silica gel using hexanes as an eluent, yielding the *trans* adduct **36** as a viscous clear oil. Yield: 9 mg (14.9%).

#### Photolysis with No Trapping Agent

Precursor **32** (15 mg, 0.046 mmol) was dissolved in 1 mL of benzene-*d*_6_ in a quartz NMR tube, and 5.0 μL of 1,3-benzodioxole was added to the tube for use as an internal standard. Photolysis was monitored by NMR until all **32** had reacted or 6 h. Yields of **34** (7.6 mg, 0.023 mmol, 49%), **9** (1.5 mg, 0.0051 mmol, 21%), and phenanthrene (4.1 mg, 0.023 mmol 49%) were thus determined.

#### Photolysis with 1,3-Diphenylisobenzofuran

Precursor **32** (49 mg, 0.15 mmol) and 1,3-diphenylisobenzofuran (41 mg, 0.15 mmol) were dissolved in 2 mL of benzene in a long-necked quartz cuvette. Photolysis was monitored by GC-MS and MS until all **32** had reacted or 20 h. No evidence of trapped **10** was observed. To isolate the rearranged precursor **32**, the crude product was purified first by flash column chromatography on silica gel using hexanes as an eluent, then via manual columns on silica treated with 33% w/w AgNO_3_ using hexanes/ethyl acetate. **34** was obtained as a waxy solid. Yield: 17 mg (35.0%).

#### Photolysis with *t*-Butanol

Precursor **32** (33 mg, 0.10 mmol) and an excess of t-butanol (0.5 mL) were dissolved in 1 mL of benzene in a long-neck quartz cuvette. Photolysis was monitored by GC-MS and MS until all **32** had reacted or 2 h. No evidence of ethers **7** or **8** was observed.

## Data Availability

The data underlying this study are available in the published article and its Supporting Information.

## Supporting Information Available

Experimental procedures, spectral data, computational figures, and Cartesian coordinates of the relevant optimized structures and transition states (PDF)
