# Supplementary material for: Adamantylidenecarbene: Photochemical Generation, Trapping, and Theoretical Studies
Source: J Org Chem. 2023 Sep 28;88(20):14413–22. doi: 10.1021/acs.joc.3c01399 (PMC10594661; doi:10.1021/acs.joc.3c01399)
Supplement: Supplementary file 1 — jo3c01399_si_001.pdf [file jo3c01399_si_001.pdf]

ELECTRONIC SUPPORTING INFORMATION

Adamantylidenecarbene: Photochemical Generation, Trapping, and Theoretical Studies

Alexander D. Roth, Christine E. Wamsley, Sarah M. Haynes, and Dasan M. Thamattoor*

[†]*Department of Chemistry, Colby College, Waterville, ME 04901 USA*

*dmthamat@colby.edu

Table of Contents

(1) Characterization data for *1-(2-adamantylidene)-1a,9b-dihydro-1H-cyclopropa[l]phenanthrene* **(32)**

(a) GC-MS data……………………………………………………………...S4
(b) ^{1}H NMR spectrum……………………………………………………S5
(c) ^{13}C NMR spectrum…………………………………………………....S6
(d) FTIR spectrum………………………………………………………...S7
(e) Crystal structure and salient data……………………………………...S8

(2) Characterization data for *2-(5H-dibenzo[a,c][7]annulen-5-ylidene)adamantane* **(34)**

(a) GC-MS data……………………………………………………………...S9
(b) ^{1}H NMR spectrum……………………………………………………S10
(c) ^{13}C NMR spectrum…………………………………………………....S11
(d) FTIR spectrum………………………………………………………..S12

(3) Characterization data for *cis-1,1-Dichloro-2-isopropyl-3-methylcyclopropane* **(37)**

(a) GC-MS data……………………………………………………………..S13
(b) ^{1}H NMR spectrum……………………………………………………S14
(c) ^{13}C NMR spectrum…………………………………………………...S15
(d) FTIR spectrum………………………………………………………..S16

(4) Characterization data for *cis-2-(2-isopropyl-3-methylcyclopropylidene)adamantane* **(35)**

(a) GC-MS data……………………………………………………………..S17
(b) ^{1}H NMR spectrum……………………………………………………S18
(c) ^{13}C NMR spectrum…………………………………………………...S19
(d) FTIR spectrum………………………………………………………..S20

(5) Characterization data for *trans-1,1-Dichloro-2-isopropyl-3-methylcyclopropane* **(38)**

(a) GC-MS data……………………………………………………………..S21
(b) ^{1}H NMR spectrum……………………………………………………S22
(c) ^{13}C NMR spectrum…………………………………………………...S23
(d) FTIR spectrum………………………………………………………..S24

(6) Characterization data for *trans-2-(2-isopropyl-3-methylcyclopropylidene)adamantane* **(36)**

(a) GC-MS data……………………………………………………………..S25

(b) ^{1}H NMR spectrum……………………………………………………………S26
(c) ^{13}C NMR spectrum…………………………………………………………...S27
(d) FTIR spectrum……………………………………………………………..S28

(7) Photolysis Data
(a) GC-MS data for photolysis of **32** in *cis*-4-methyl-2-pentene (t= 3hrs)..………...S29
(b) GC-MS data for photolysis of **32** in *trans*-4-methyl-2-pentene (t=1.5 hrs)….….S30
(c) GC-MS data for photolysis of **32** in 1,3-diphenylisobenzofuran (t=20 hrs)……..S31
(d) GC-MS data for pure sample of **9**……………………………………………..S32

(8) Computational Data
(a) Adamantylidene System
(i) Optimized energies, coordinates, stability analyses, and frequencies for B2PLYP/def2-TZVP calculations..…………..………………………....S33-39
(ii) Optimized energies, coordinates, stability analyses, and frequencies for B3LYP/def2-TZVP calculations…….………………………………..S39-44
(iii) Optimized energies, coordinates, stability analyses, and frequencies for PBE0/def2-TZVP calculations……..…………………………………S44-49
(iv) Optimized energies, coordinates, stability analyses, and frequencies for ωB79x-D3BJ/def2-TZVP calculations……………………………….……S49-56
(v) Single point energies and T1 diagnostics for DLPNO-CCSD(T)/def2-TZVP// B2PLYP/def2-TZVP calculations..…………………….……….….……S56
(vi) Single point energies and T1 diagnostics for DLPNO-CCSD(T)/def2-TZVP// B3LYP/def2-TZVP calculations…………………………...…….….……S56-57
(vii) Single point energies and T1 diagnostics for DLPNO-CCSD(T)/def2-TZVP// PBE0/def2-TZVP calculations..…………………………….………/……..…S57
(viii) Single point energies and T1 diagnostics for DLPNO-CCSD(T)/def2-TZVP//ωB79x-D3BJ/def2-TZVP calculations…………………….………S57-58
(b) Cyclohexylidene System
(i) Optimized energies, coordinates, stability analyses, and frequencies for B2PLYP/def2-TZVP calculations..…………..……………………………....S58-62
(ii) Single point energies and T1 diagnostics for DLPNO-CCSD(T)/def2-TZVP// B2PLYP/def2-TZVP calculations..………………………….……….…….…S62-63
(c) 4-Methylcyclohexylidene System
(i) Optimized energies, coordinates, stability analyses, and frequencies for B2PLYP/def2-TZVP calculations..…………..………………………....S63-67
(ii) Single point energies and T1 diagnostics for DLPNO-CCSD(T)/def2-TZVP// B2PLYP/def2-TZVP calculations..…………………….……….…..……S67-68
(d) ω-camphylidene System
(i) Optimized energies, coordinates, stability analyses, and frequencies for B2PLYP/def2-TZVP calculations..…………..………………………....S68-73
(ii) Single point energies and T1 diagnostics for DLPNO-CCSD(T)/def2-TZVP// B2PLYP/def2-TZVP calculations..…………………….……….….……S73
(e) ω-longifolene System
(i) Optimized energies, coordinates, stability analyses, and frequencies for B2PLYP/def2-TZVP calculations..…………..………………………....S73-79
(ii) Single point energies and T1 diagnostics for DLPNO-CCSD(T)/def2-TZVP// B2PLYP/def2-TZVP calculations..…………………….………..……S79

(9) Potential Energy Surface Diagrams
Adamantylidene System
(i) DLPNO-CCSD(T)/def2-TZVP//B2PLYP/def2-TZVP calculations…...…..S80
(ii) DLPNO-CCSD(T)/def2-TZVP//B3LYP/def2-TZVP calculations…......…S81
(iii) DLPNO-CCSD(T)/def2-TZVP//PBE0/def2-TZVP calculations………....S82
(iv) DLPNO-CCSD(T)/def2-TZVP//ωB79x-D3BJ/def2-TZVP calculations....S83

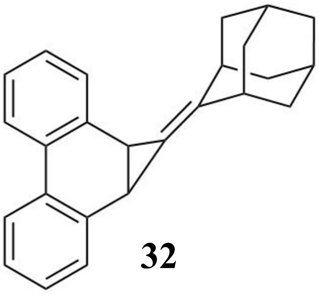

32

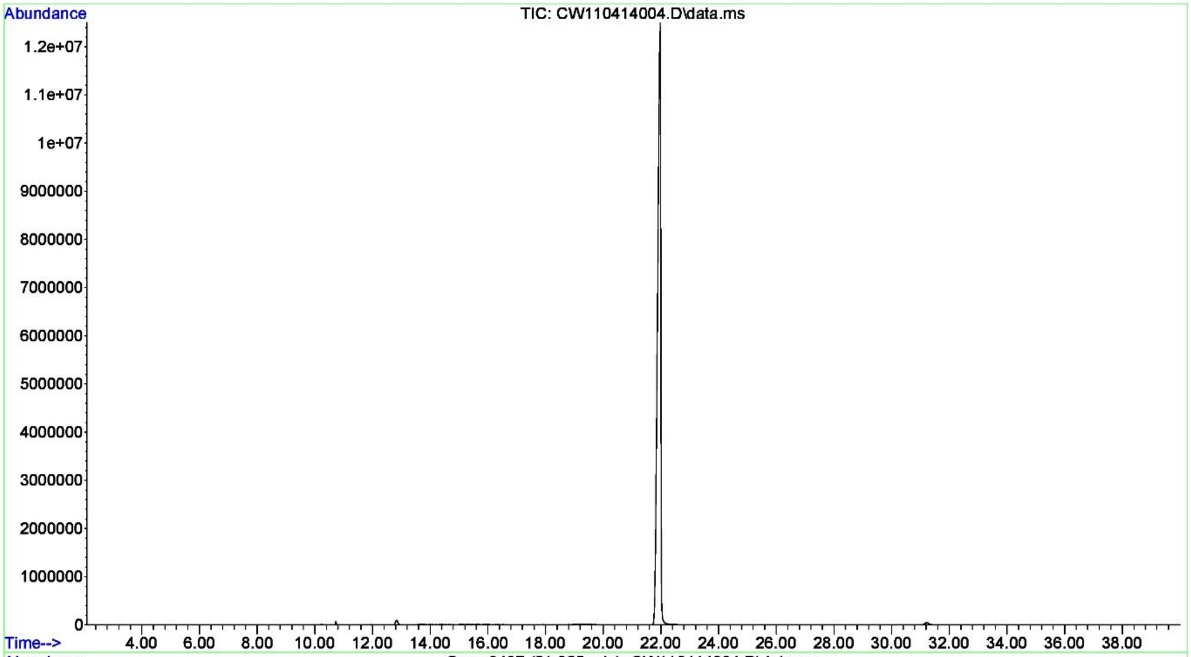
Abundance
TIC: CW110414004.D\data.ms
1.2e+07
1.1e+07
1e+07
9000000
8000000
7000000
6000000
5000000
4000000
3000000
2000000
1000000
0
Time-->
4.00 6.00 8.00 10.00 12.00 14.00 16.00 18.00 20.00 22.00 24.00 26.00 28.00 30.00 32.00 34.00 36.00 38.00

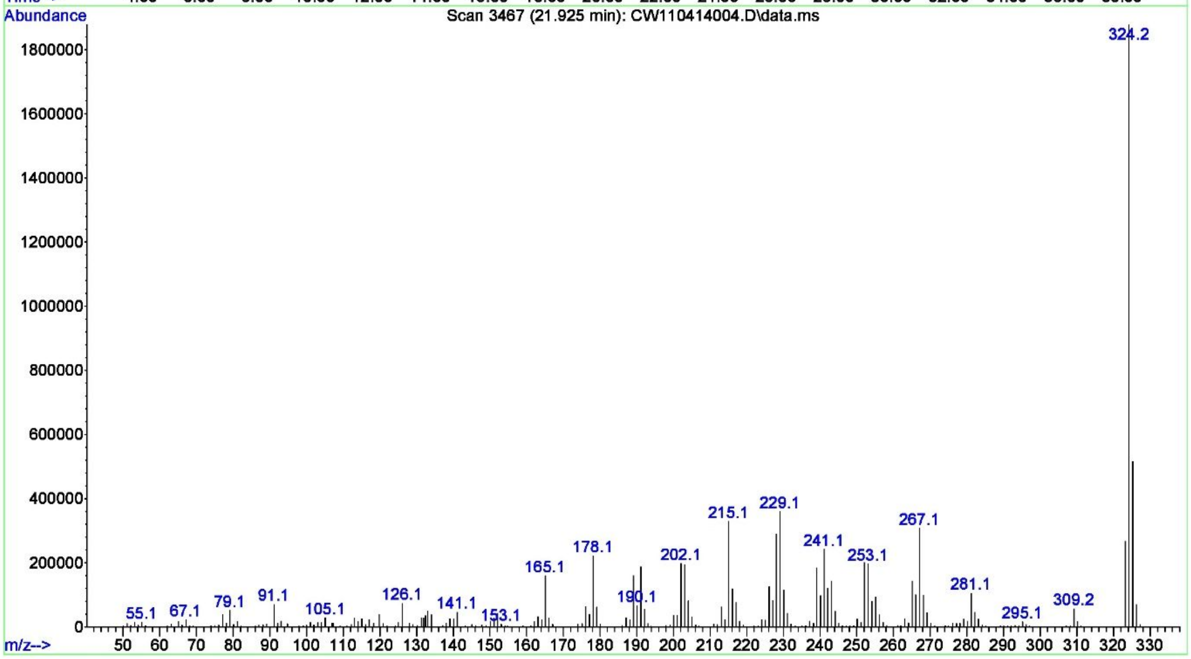
Abundance
Scan 3467 (21.925 min): CW110414004.D\data.ms
1800000
1600000
1400000
1200000
1000000
800000
600000
400000
200000
0
324.2
215.1
229.1
267.1
241.1
253.1
178.1
202.1
165.1
190.1
281.1
126.1
141.1
91.1
79.1
67.1
55.1
105.1
153.1
295.1
309.2
m/z-->
50 60 70 80 90 100 110 120 130 140 150 160 170 180 190 200 210 220 230 240 250 260 270 280 290 300 310 320 330

^{1}H NMR, 500 MHz, $CDCl_3$

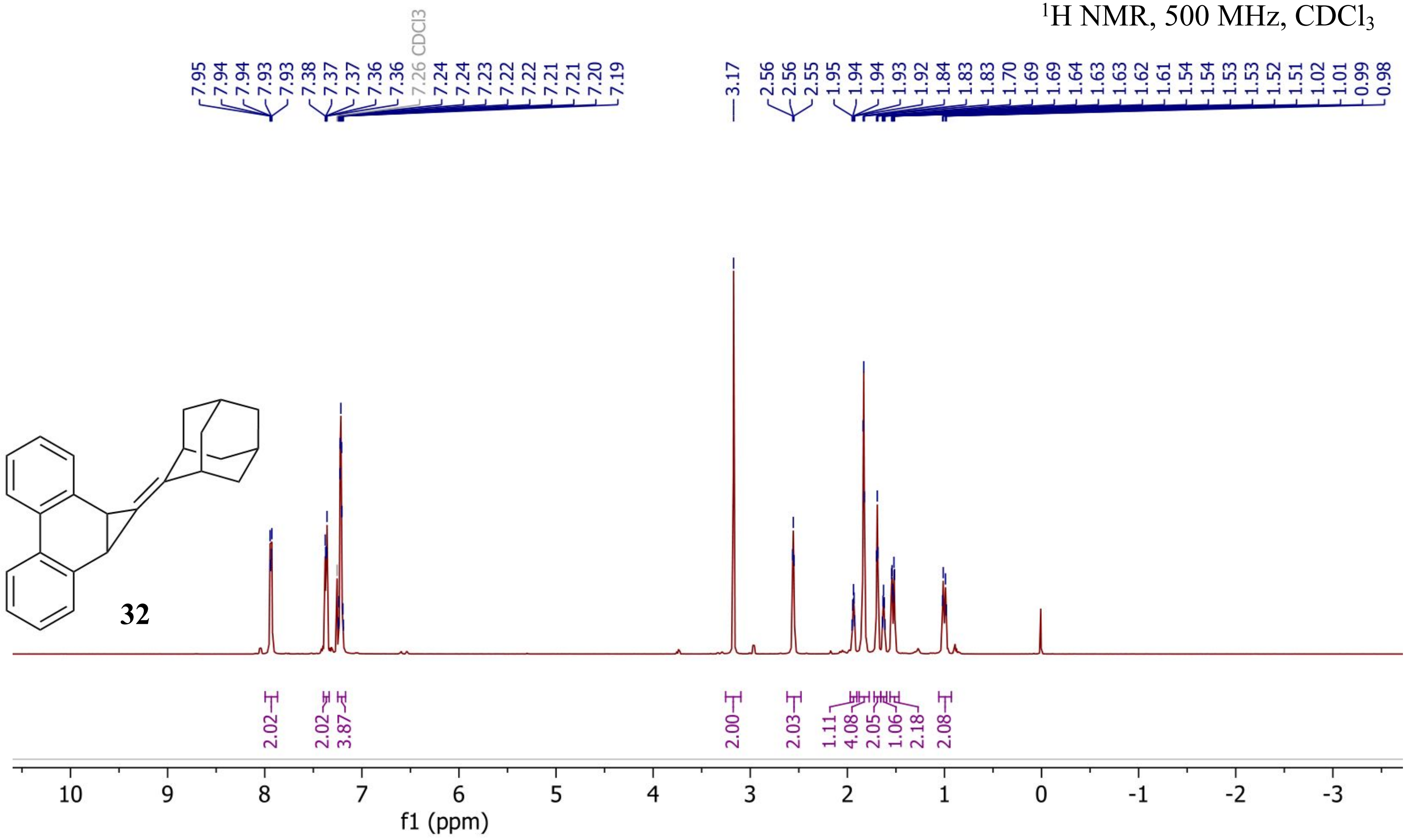

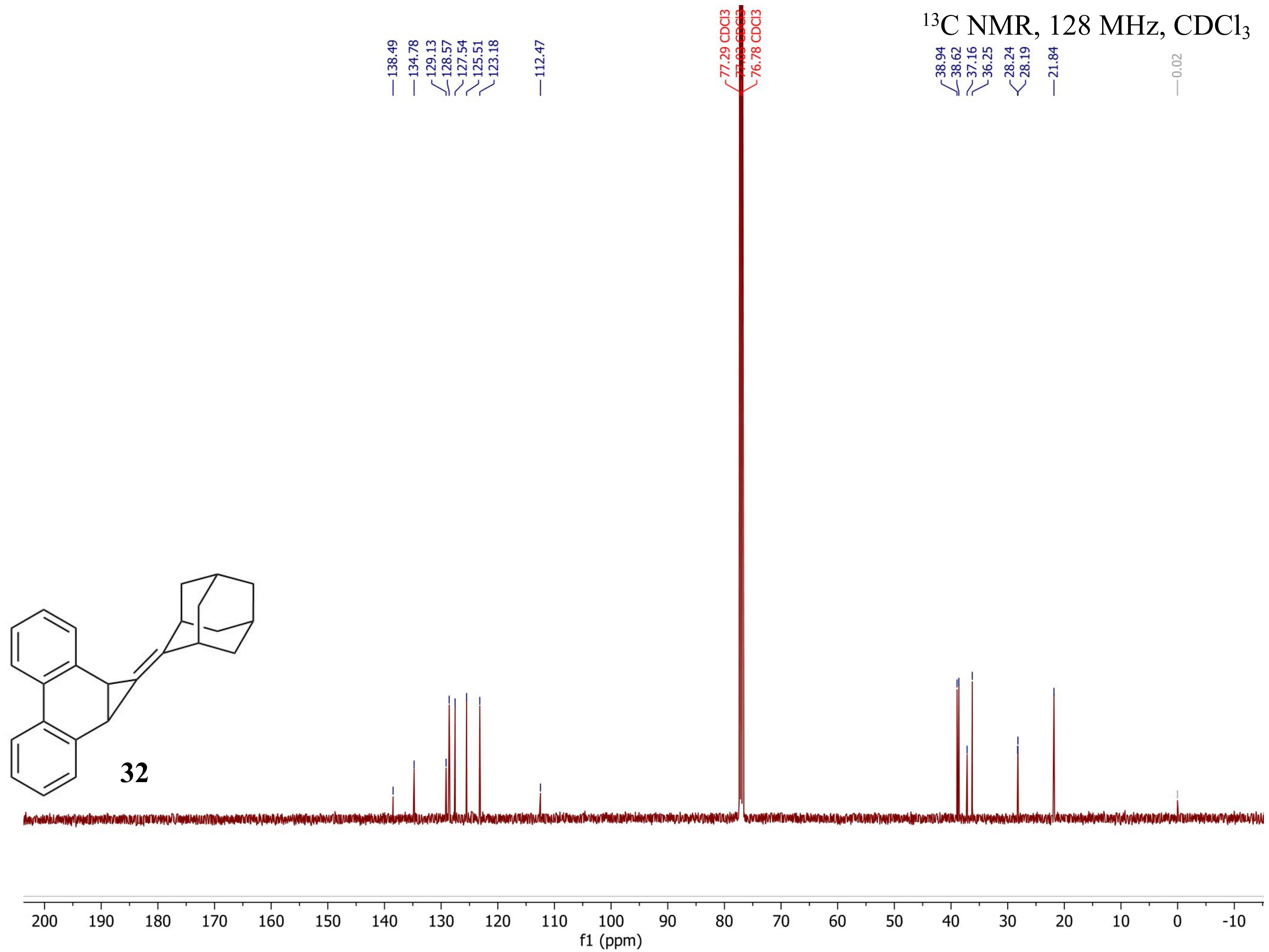

^{13}C NMR, 128 MHz, $CDCl_3$
138.49
134.78
129.13
128.57
127.54
125.51
123.18
112.47
77.29 CDCl3
77.03 CDCl3
76.78 CDCl3
38.94
38.62
37.16
36.25
28.24
28.19
21.84
0.02
32
200 190 180 170 160 150 140 130 120 110 100 90 80 70 60 50 40 30 20 10 0 -10
f1 (ppm)

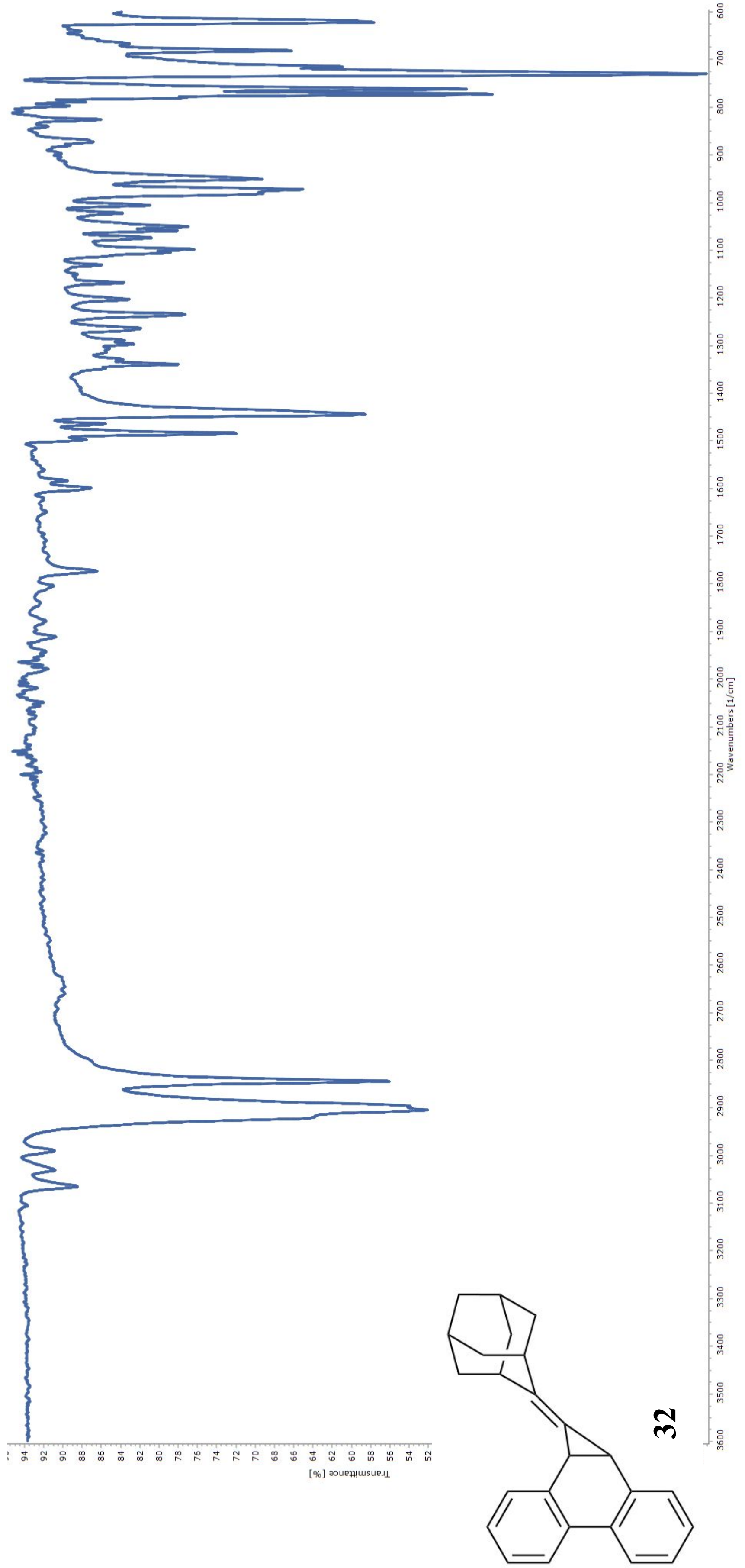

32

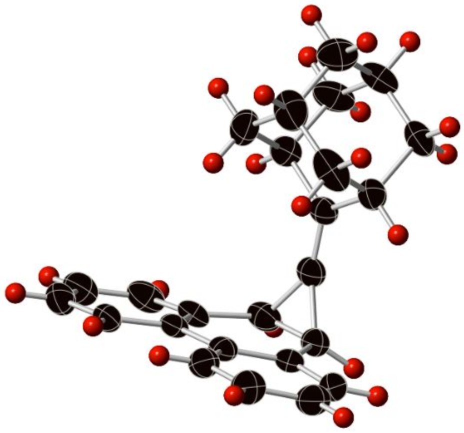

Empirical formula	$C_{25}H_{24}$
Formula weight	324.44
Temperature/K	173.15
Crystal system	orthorhombic
Space group	$P2_12_12_1$
a/Å	6.2278(15)
b/Å	11.247(3)
c/Å	25.354(6)
α/°	90
β/°	90
γ/°	90
Volume/Å^3	1775.9(7)
Z	4
ρ_{calc}g/cm^3	1.213
μ/mm^{-1}	0.068
F(000)	696.0
Crystal size/mm^3	0.37 × 0.13 × 0.08
Radiation	Mo Kα (λ = 0.71073)
2Θ range for data collection/°	3.212 to 52.768
Index ranges	$-7 \leq h \leq 7$, $-13 \leq k \leq 14$, $-31 \leq l \leq 27$
Reflections collected	14687
Independent reflections	3636 [R_{int} = 0.0341, R_{sigma} = 0.0296]
Data/restraints/parameters	3636/0/226
Goodness-of-fit on F^2	1.041
Final R indexes [I>=2σ (I)]	R_1 = 0.0403, wR_2 = 0.0857
Final R indexes [all data]	R_1 = 0.0508, wR_2 = 0.0921
Largest diff. peak/hole / e Å^{-3}	0.17/-0.14
Flack parameter	-1.3(10)
CCDC Number	2240257

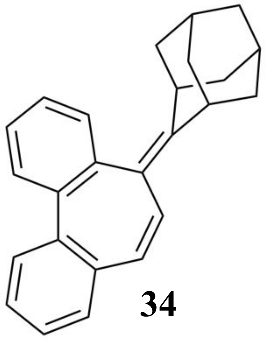

34

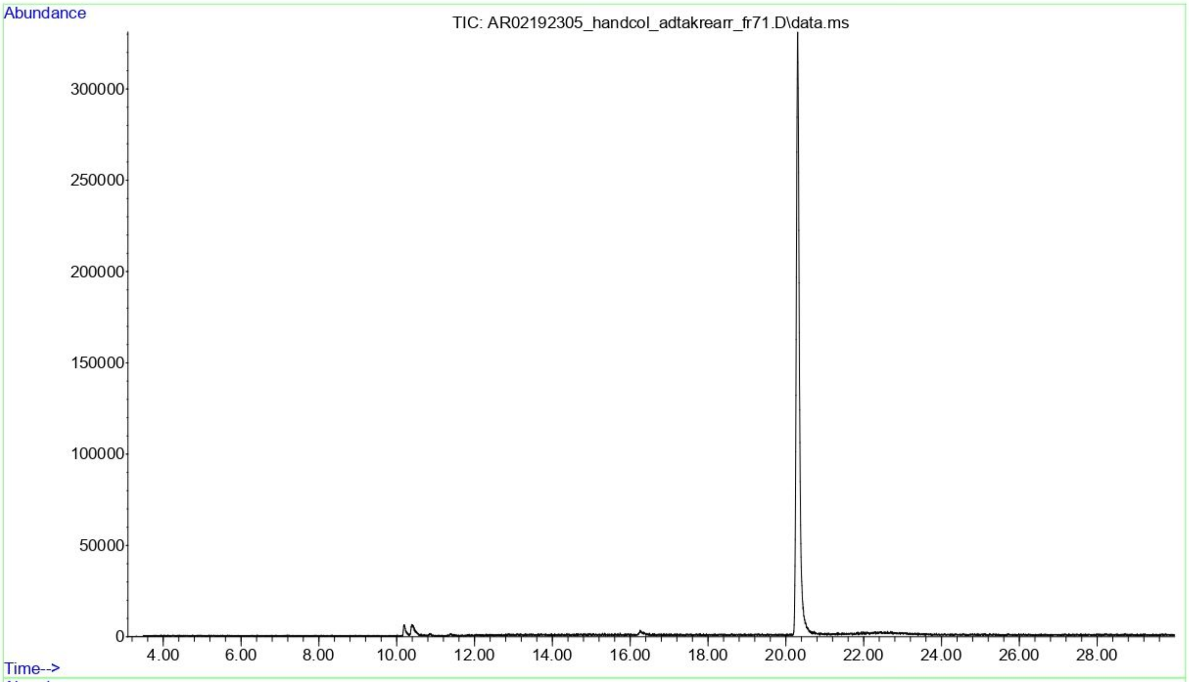
Abundance
TIC: AR02192305_handcol_adtakrearr_fr71.D\data.ms
Time-->

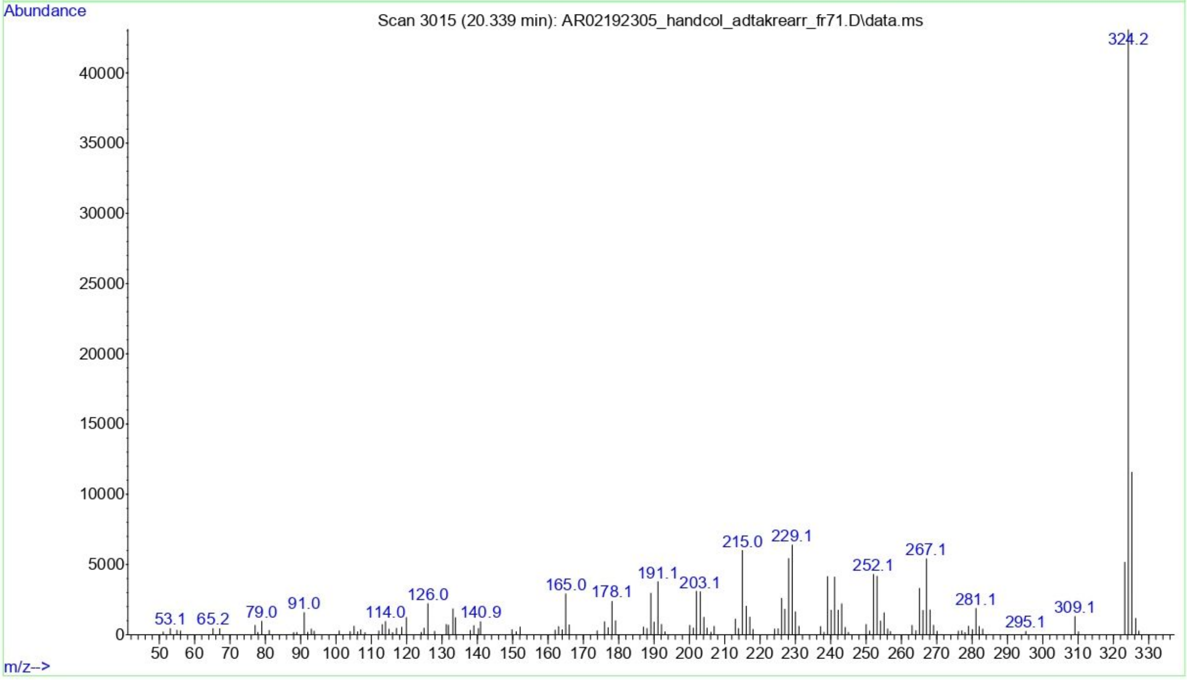
Abundance
Scan 3015 (20.339 min): AR02192305_handcol_adtakrearr_fr71.D\data.ms
324.2
m/z-->

^{1}H NMR, 500 MHz, $CDCl_3$

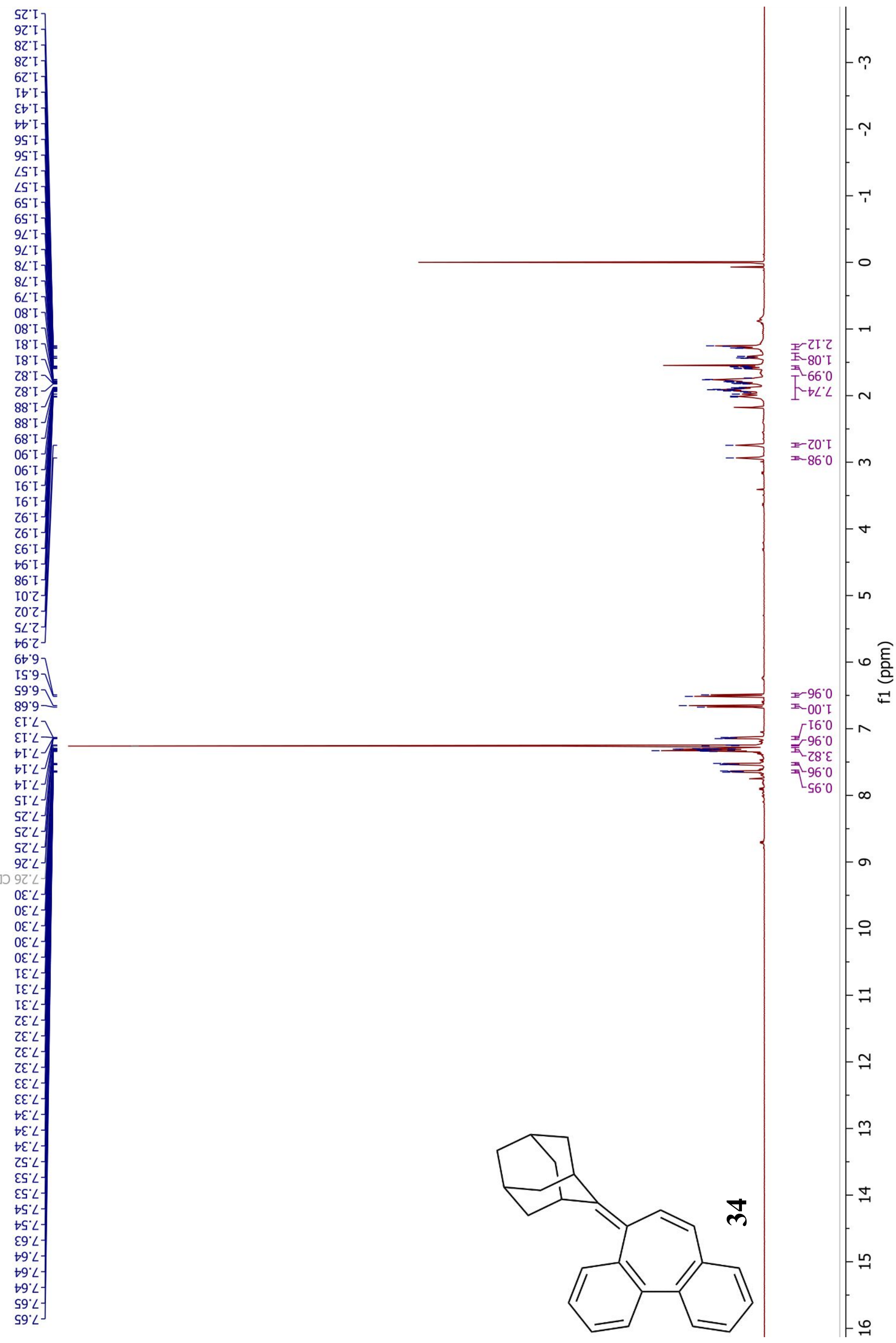

^{13}C NMR, 128 MHz, $CDCl_3$

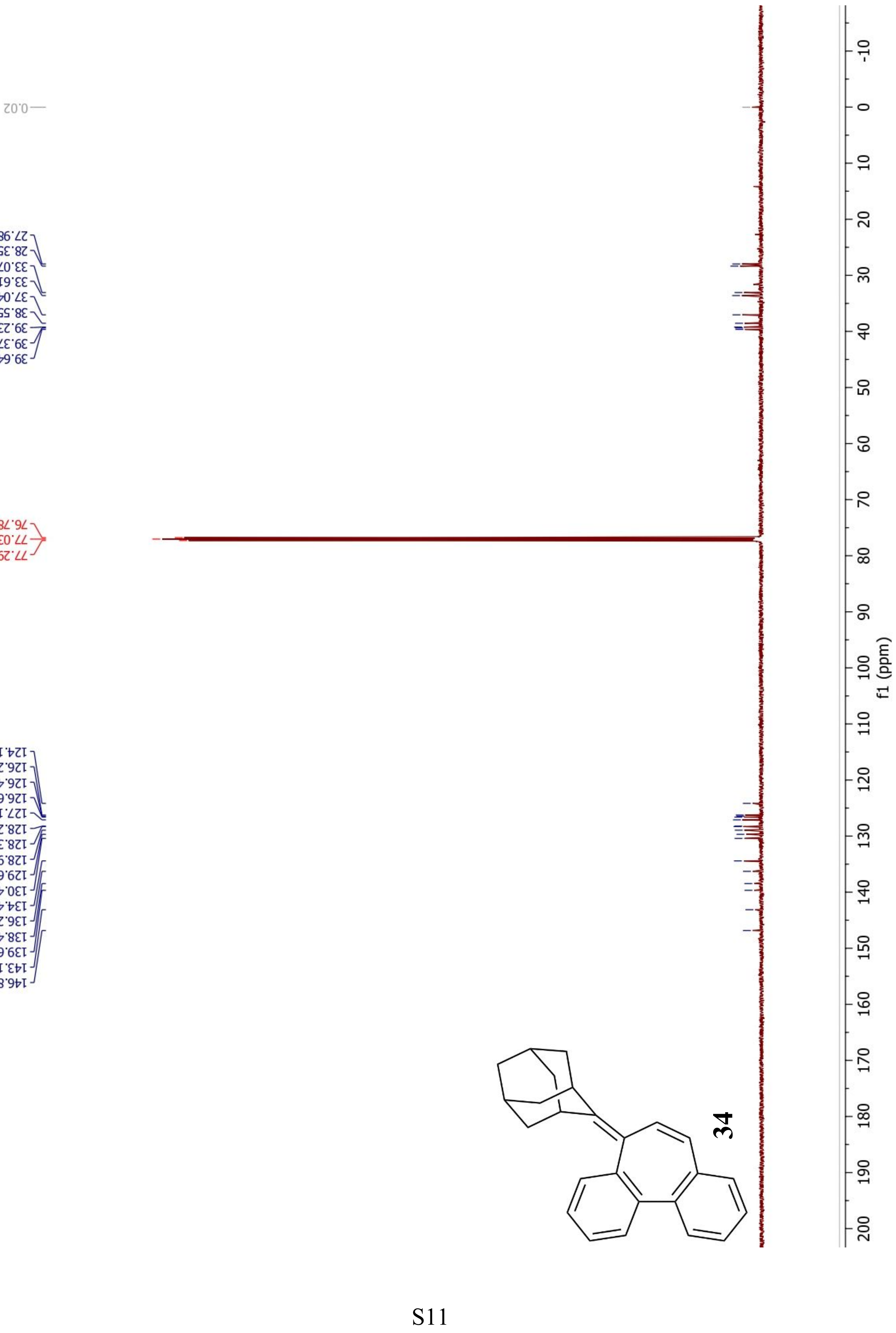

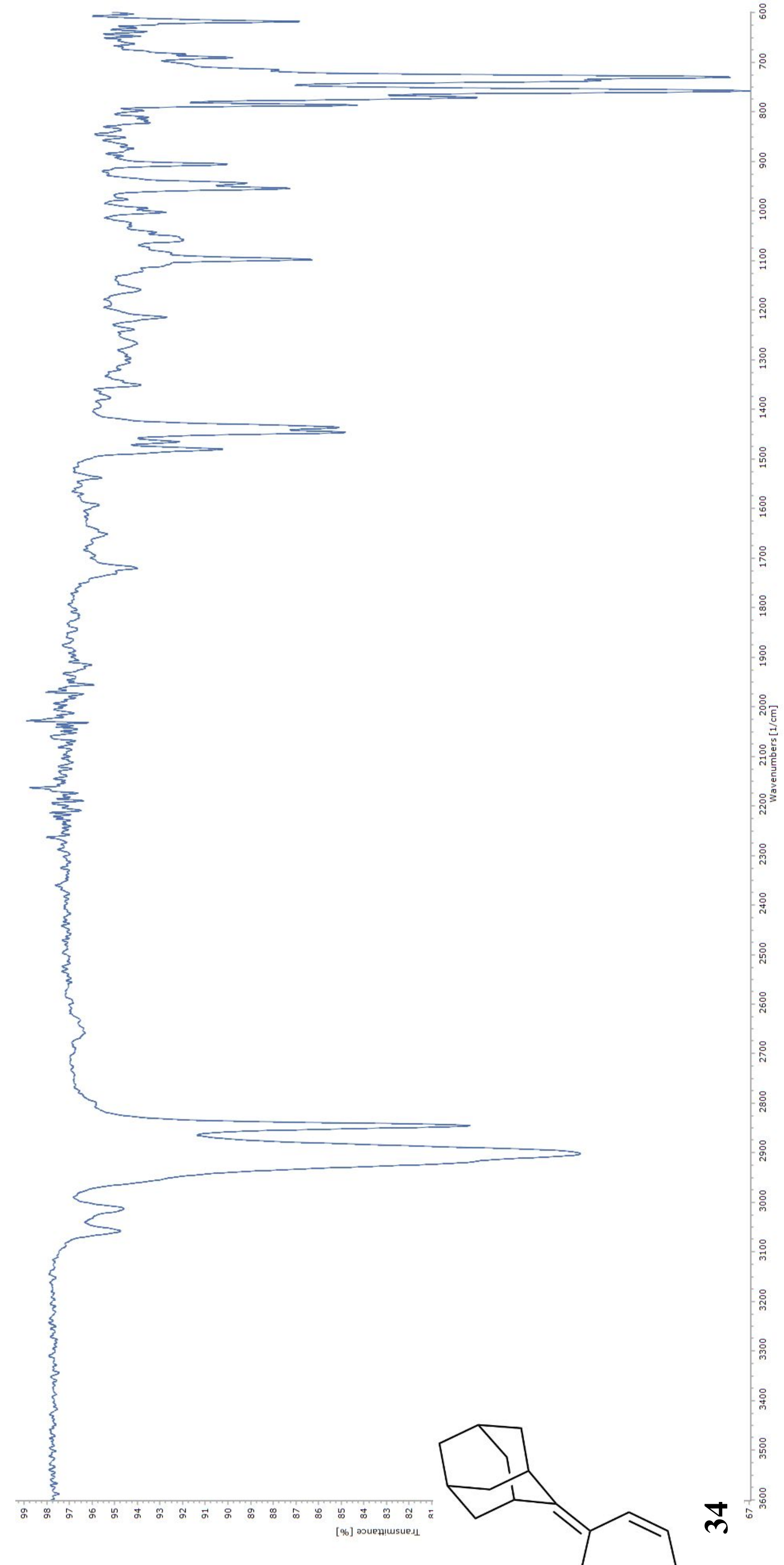

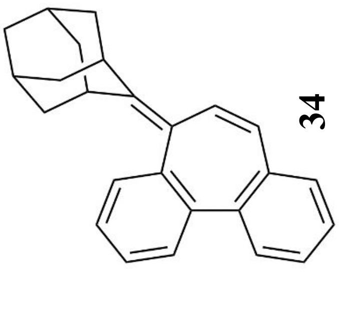

34

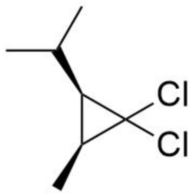

37 (racemic)

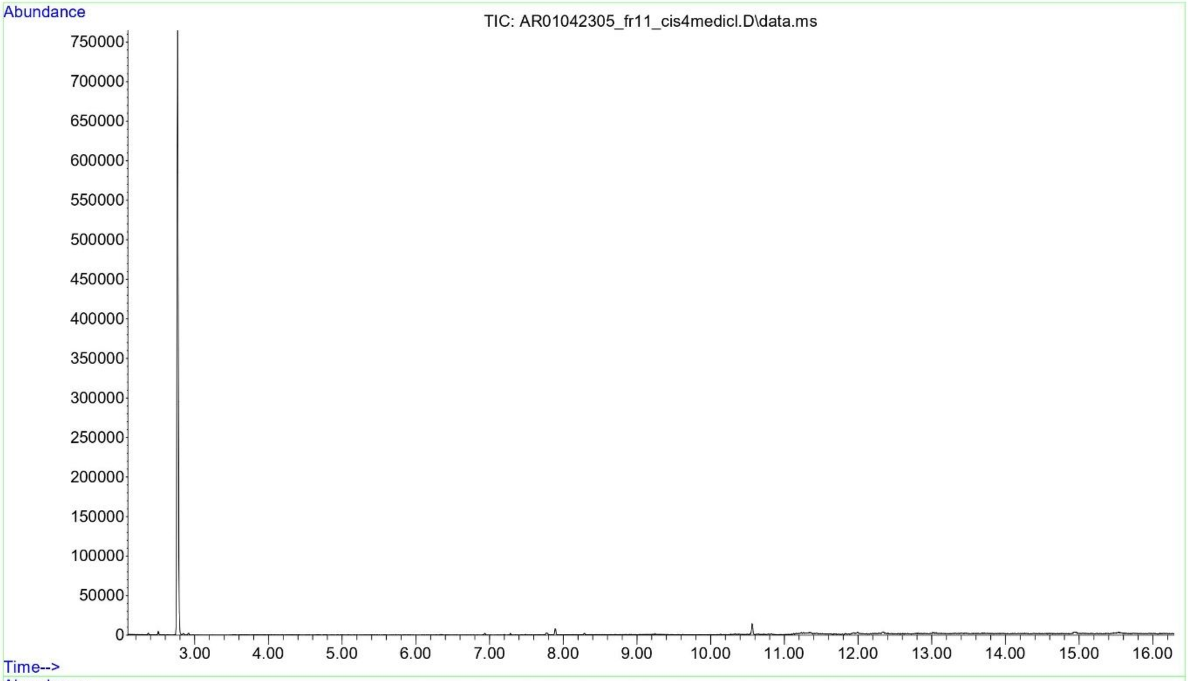

Abundance
TIC: AR01042305_fr11_cis4medicl.D\data.ms
Time-->

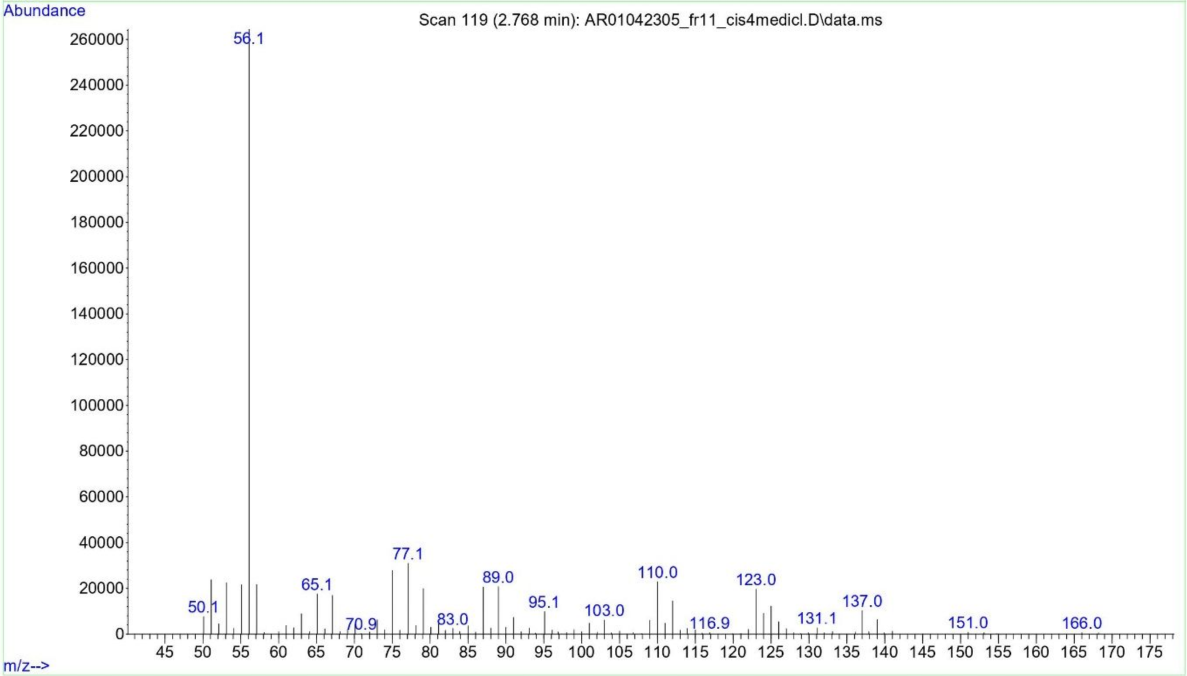

Abundance
Scan 119 (2.768 min): AR01042305_fr11_cis4medicl.D\data.ms
56.1
77.1
65.1
89.0
110.0
123.0
137.0
50.1
70.9
83.0
95.1
103.0
116.9
131.1
151.0
166.0
m/z-->

^{1}H NMR, 500 MHz, $CDCl_3$

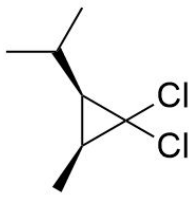

37 (racemic)

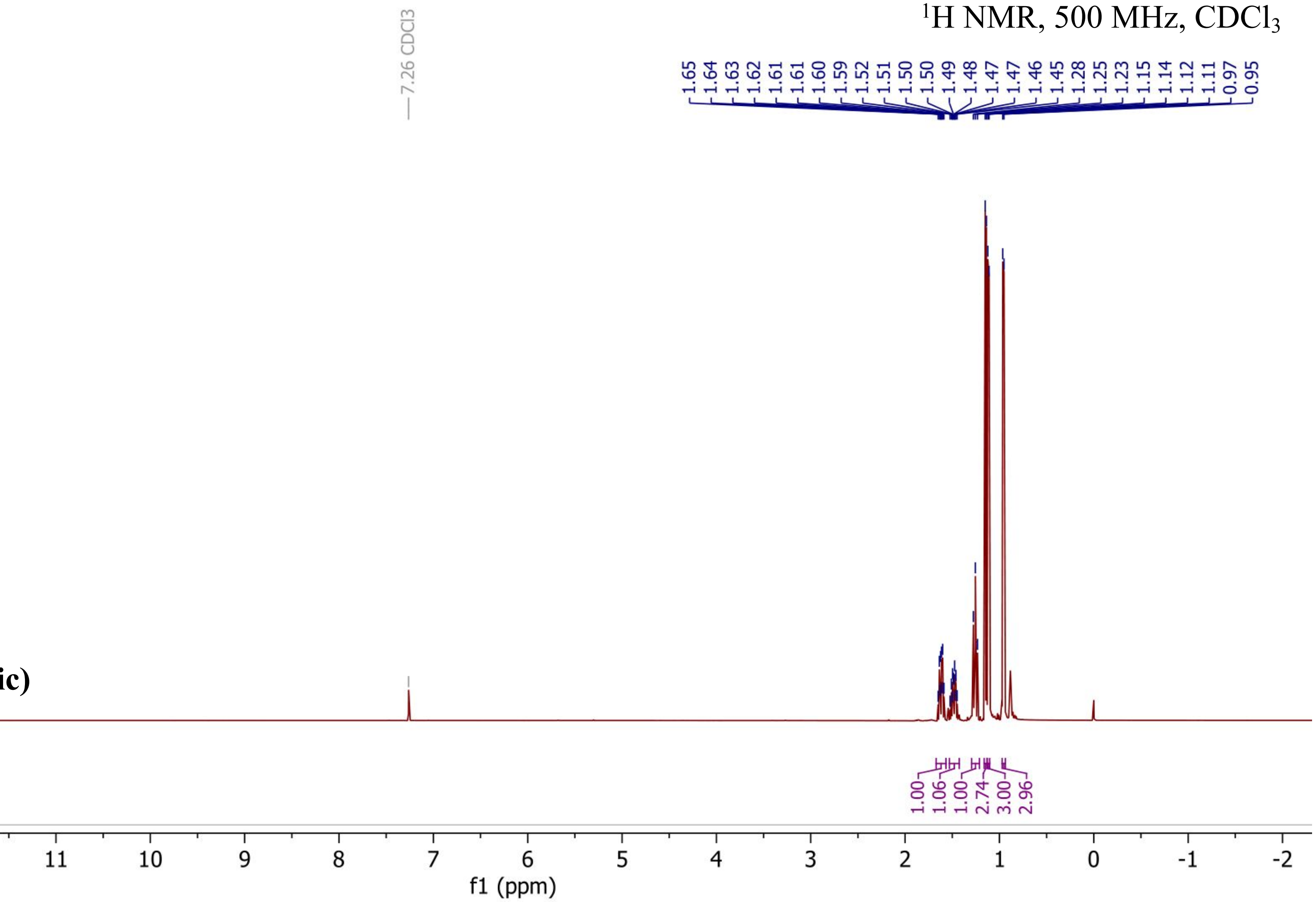

^{13}C NMR, 128 MHz, $CDCl_3$

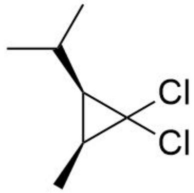

37 (racemic)

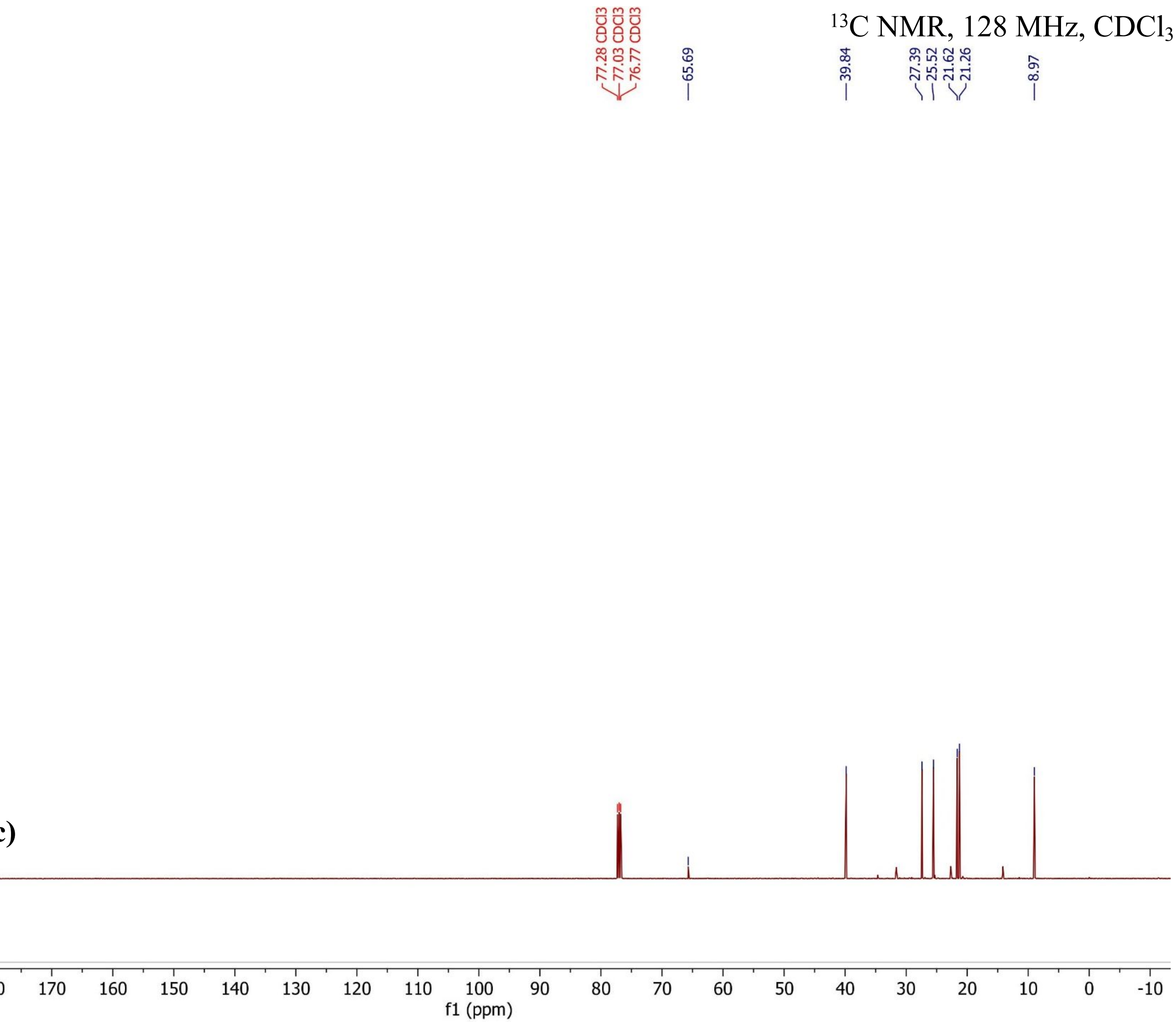

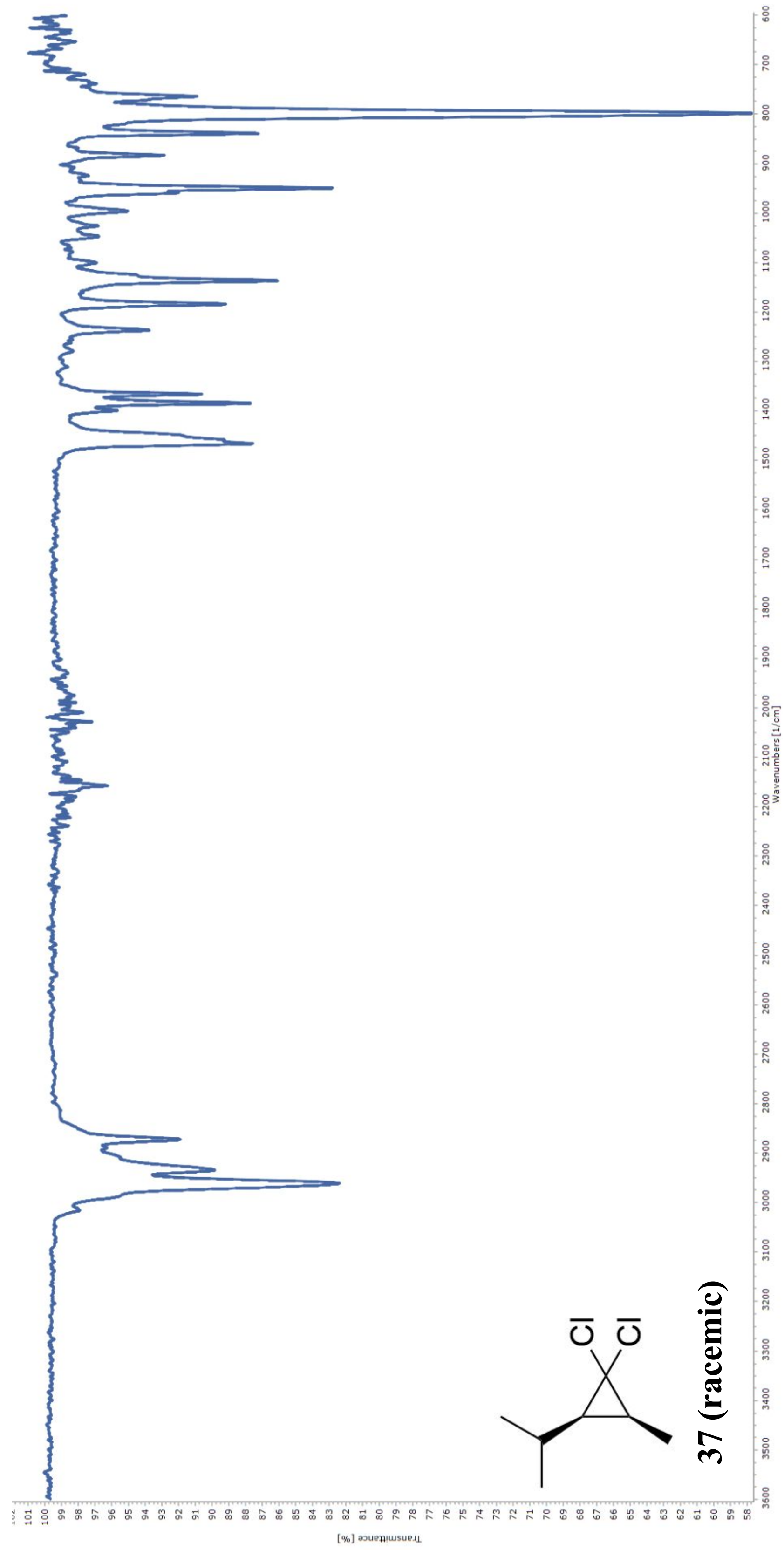

37 (racemic)

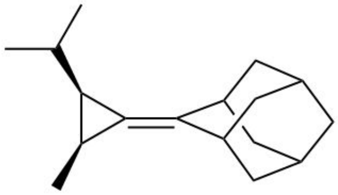

35 (racemic)

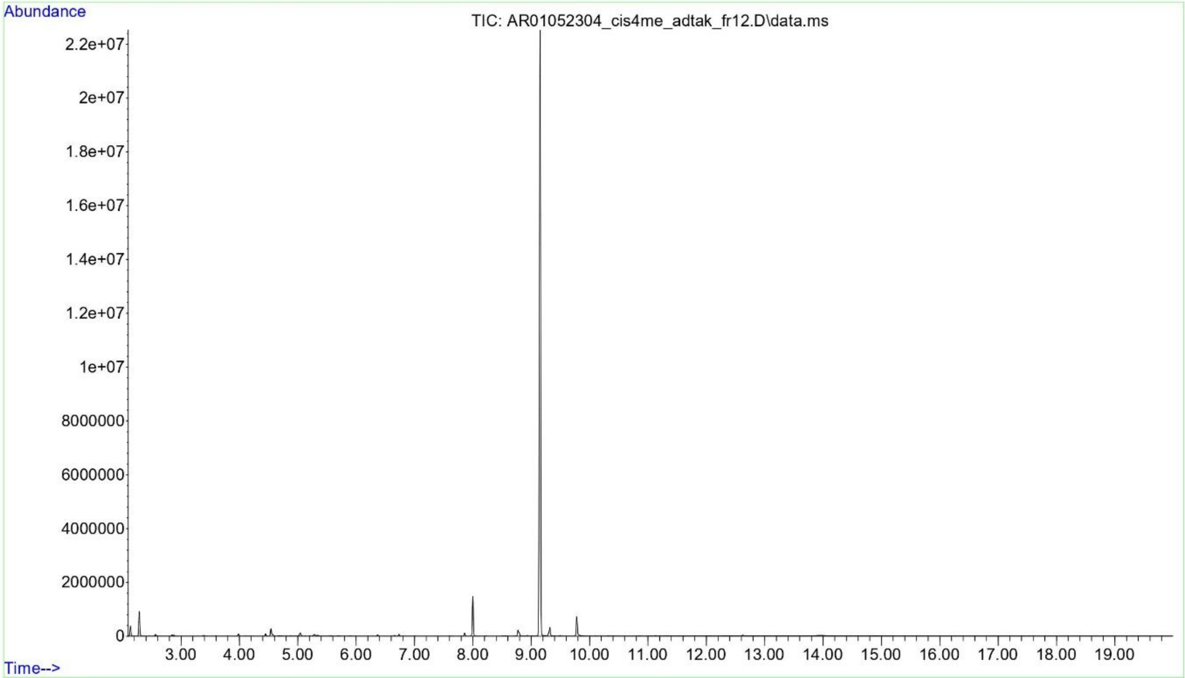
Abundance
TIC: AR01052304_cis4me_adtak_fr12.D\data.ms
2.2e+07
2e+07
1.8e+07
1.6e+07
1.4e+07
1.2e+07
1e+07
8000000
6000000
4000000
2000000
0
3.00 4.00 5.00 6.00 7.00 8.00 9.00 10.00 11.00 12.00 13.00 14.00 15.00 16.00 17.00 18.00 19.00
Time-->

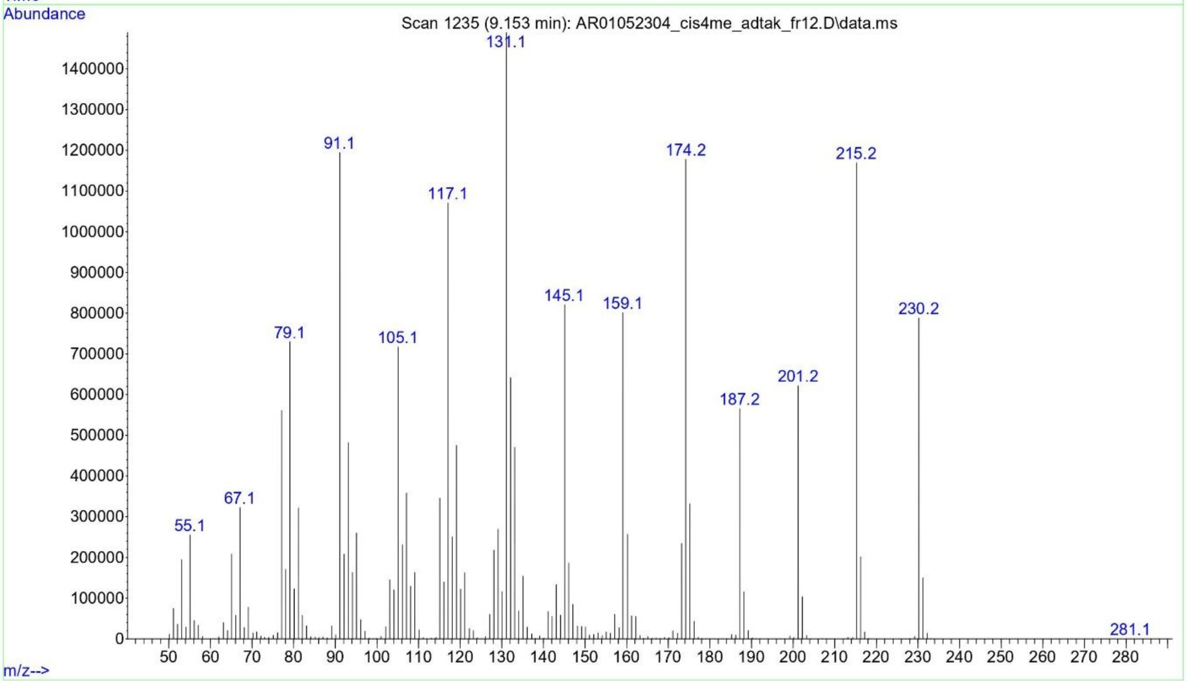
Abundance
Scan 1235 (9.153 min): AR01052304_cis4me_adtak_fr12.D\data.ms
131.1
91.1
174.2
215.2
117.1
145.1
159.1
230.2
79.1
105.1
201.2
187.2
67.1
55.1
281.1
1400000
1300000
1200000
1100000
1000000
900000
800000
700000
600000
500000
400000
300000
200000
100000
0
50 60 70 80 90 100 110 120 130 140 150 160 170 180 190 200 210 220 230 240 250 260 270 280
m/z-->

^{13}C NMR, 128 MHz, $CDCl_3$

^{1}H NMR, 500 Hz, $CDCl_3$

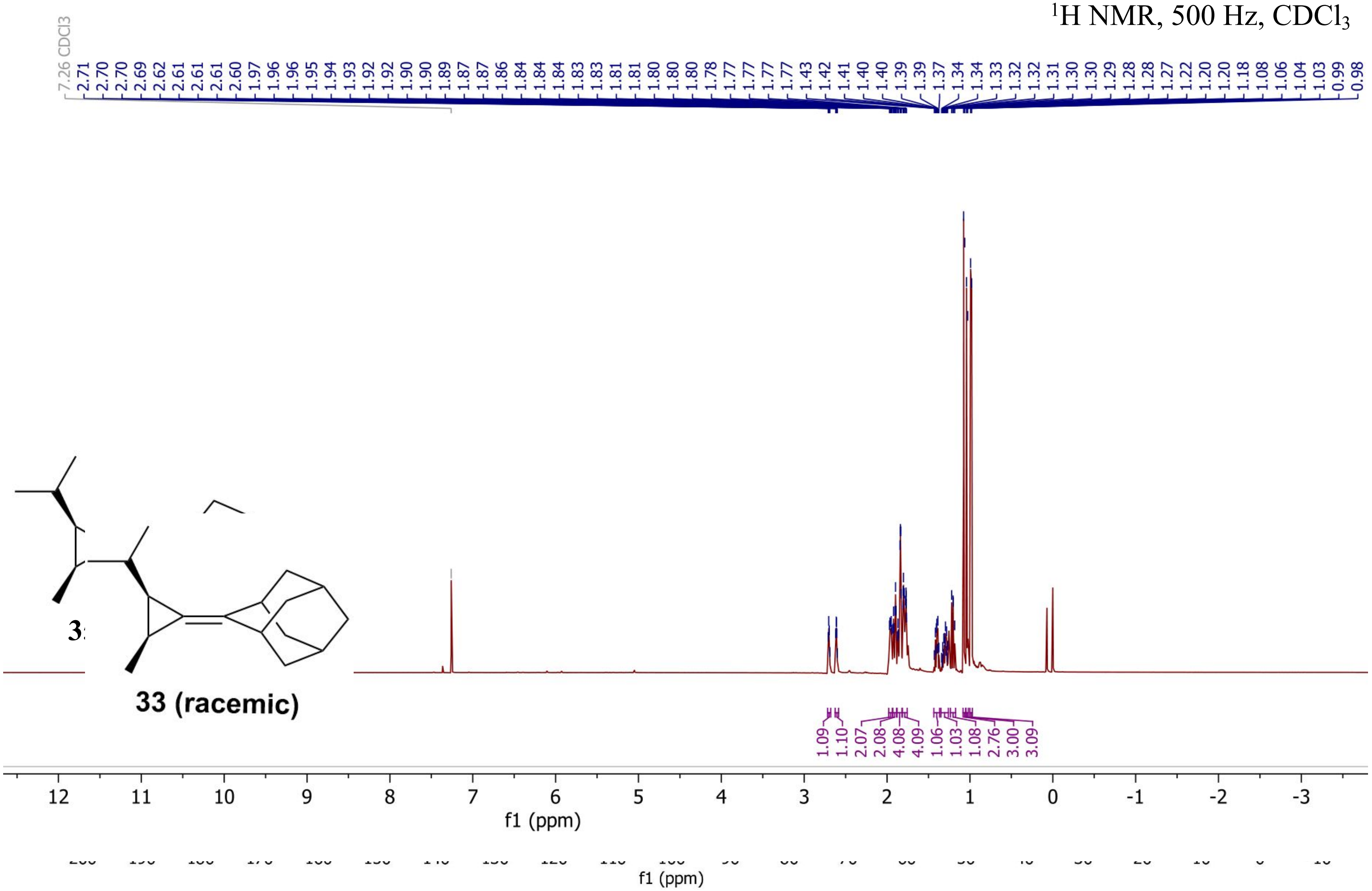

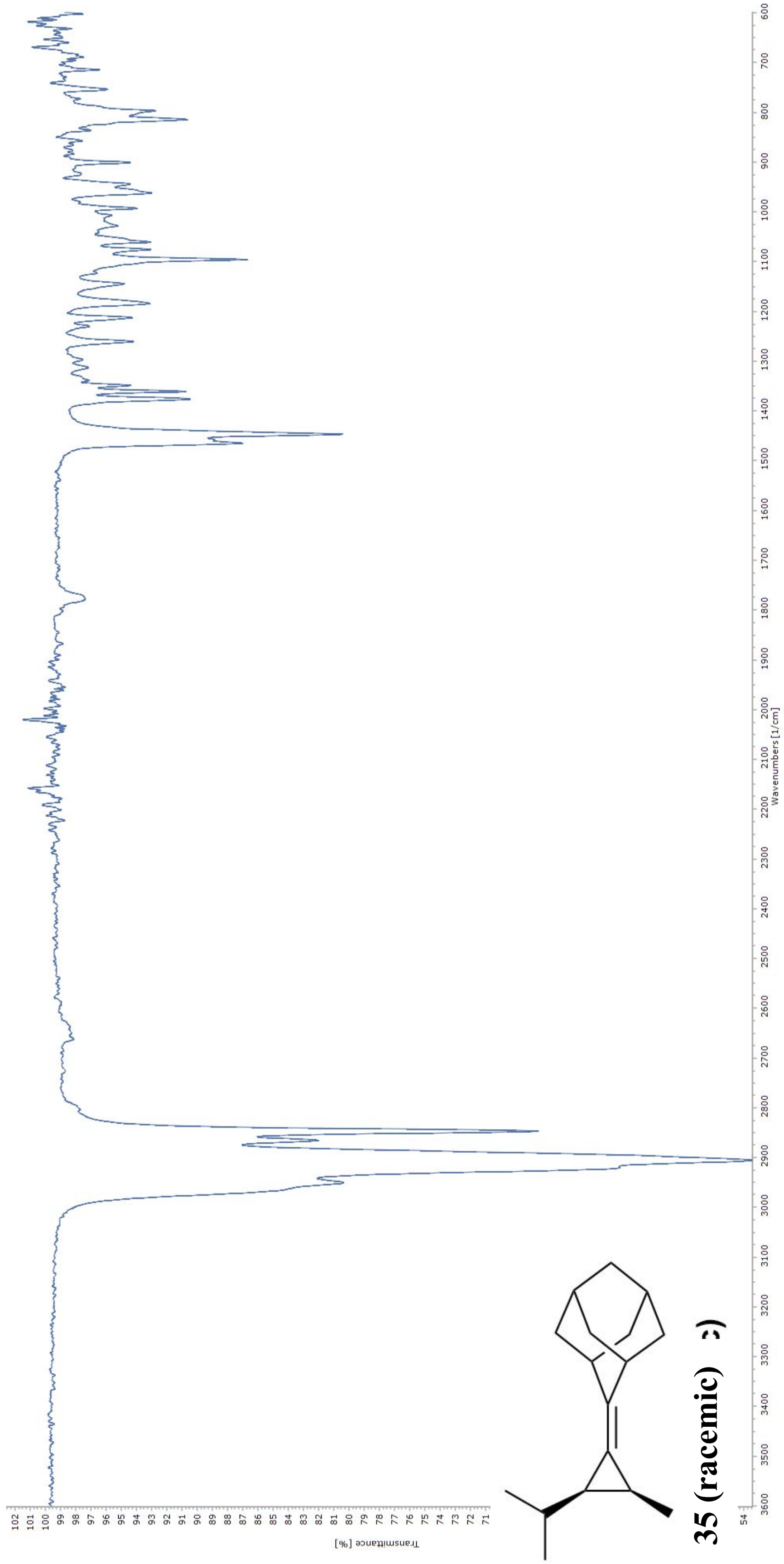

35 (racemic)

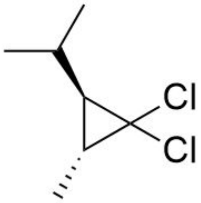

38 (racemic)

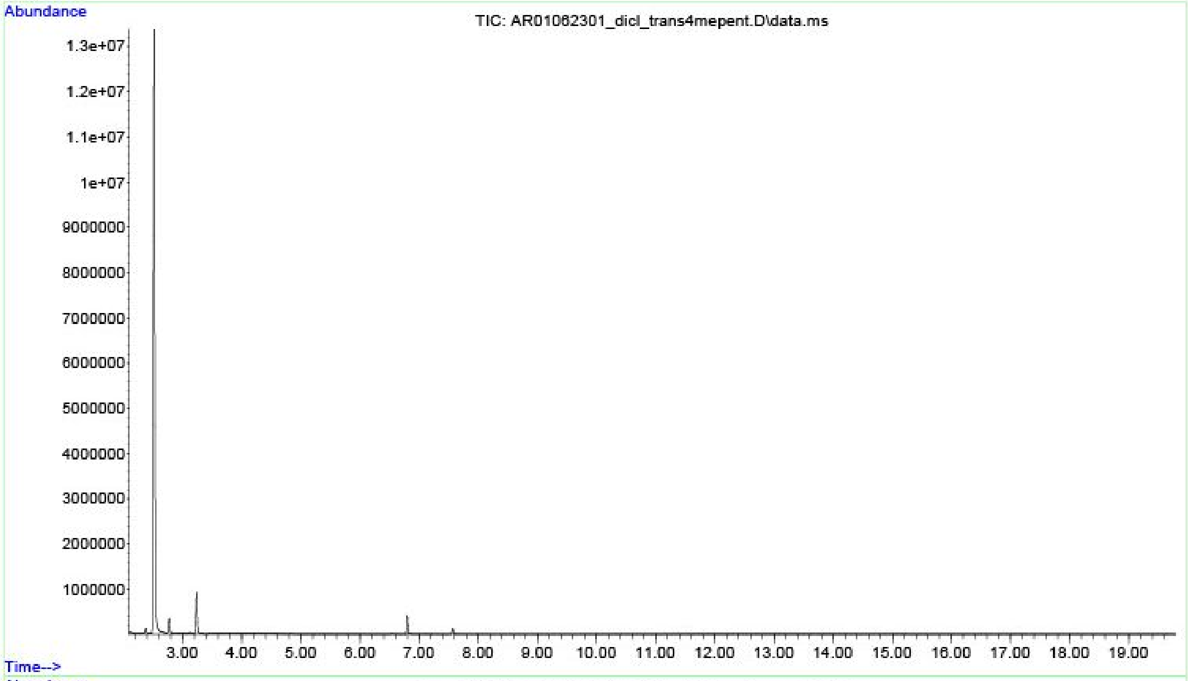

Abundance
TIC: AR01062301_dicl_trans4mepent.D\data.ms
Time-->

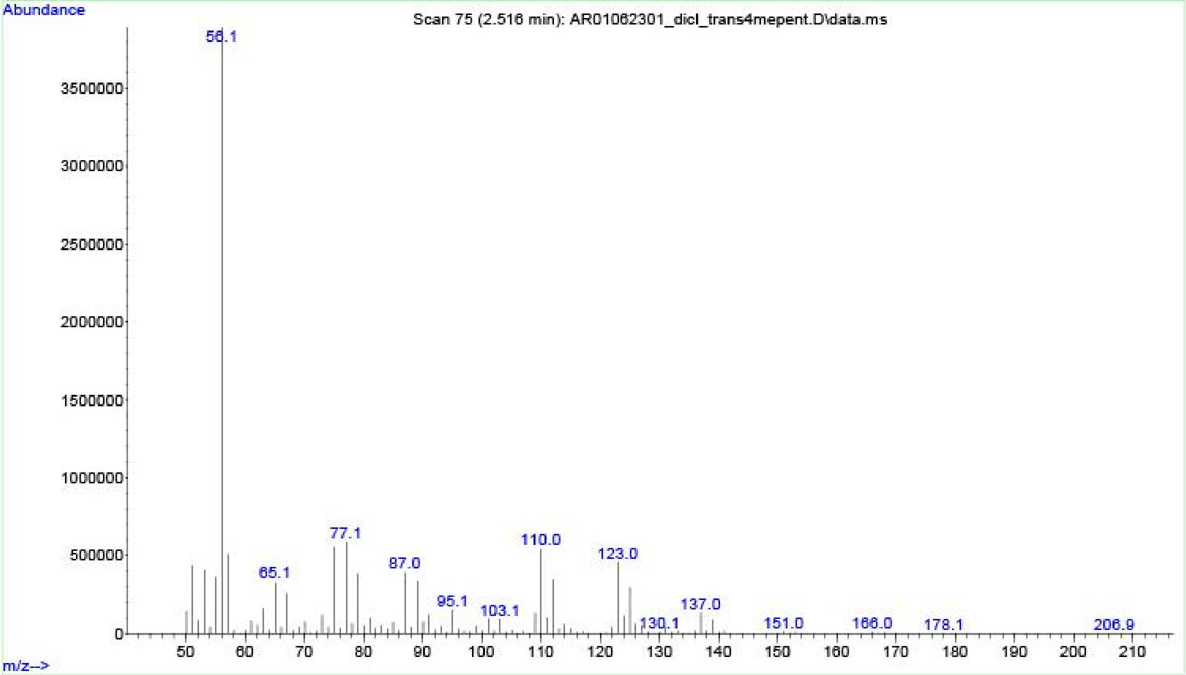

Abundance
Scan 75 (2.516 min): AR01062301_dicl_trans4mepent.D\data.ms
56.1
77.1
110.0
123.0
87.0
65.1
95.1
103.1
137.0
130.1
151.0
166.0
178.1
206.9
m/z-->

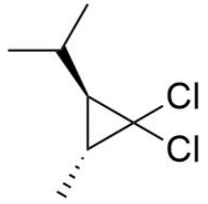
Cl
Cl

38 (racemic)

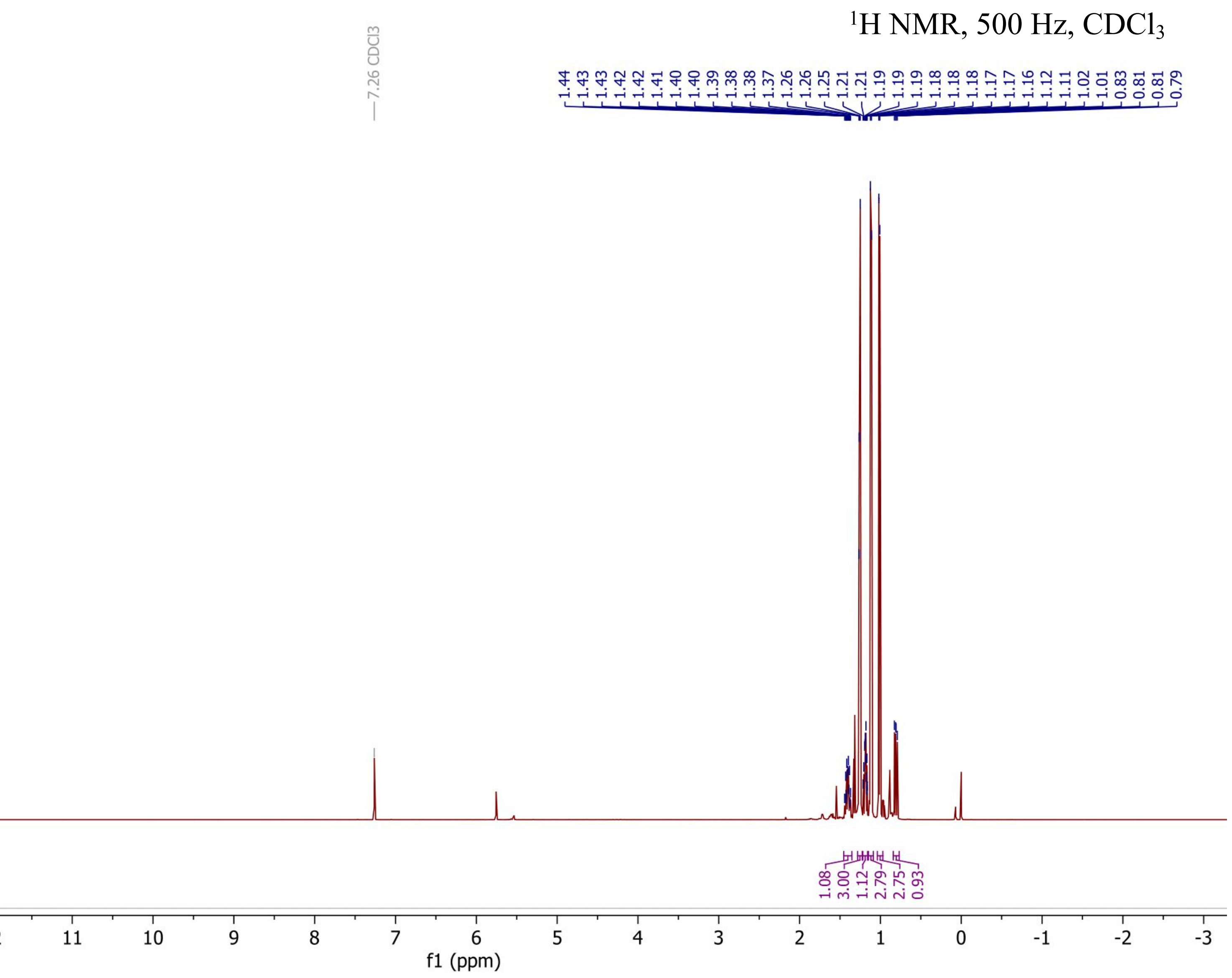
1H NMR, 500 Hz, CDCl3
7.26 CDCl3
1.44 1.43 1.43 1.42 1.42 1.41 1.40 1.40 1.39 1.38 1.38 1.37 1.26 1.26 1.25 1.21 1.21 1.19 1.19 1.19 1.18 1.18 1.18 1.17 1.17 1.16 1.12 1.11 1.02 1.01 0.83 0.81 0.81 0.79
1.08
3.00
1.12
2.79
2.75
0.93
14 13 12 11 10 9 8 7 6 5 4 3 2 1 0 -1 -2 -3
f1 (ppm)

^{13}C NMR, 128 MHz, $CDCl_3$

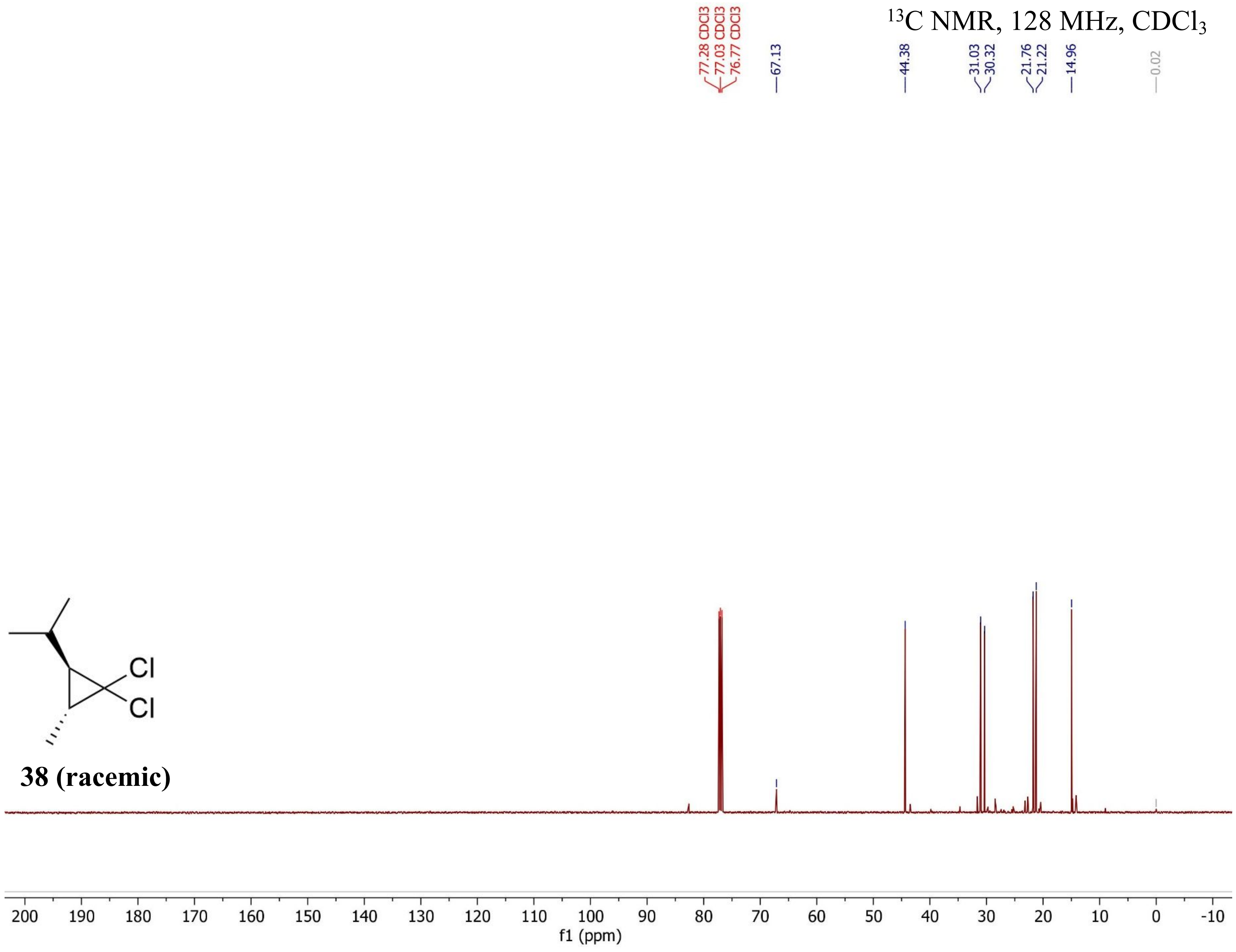

38 (racemic)

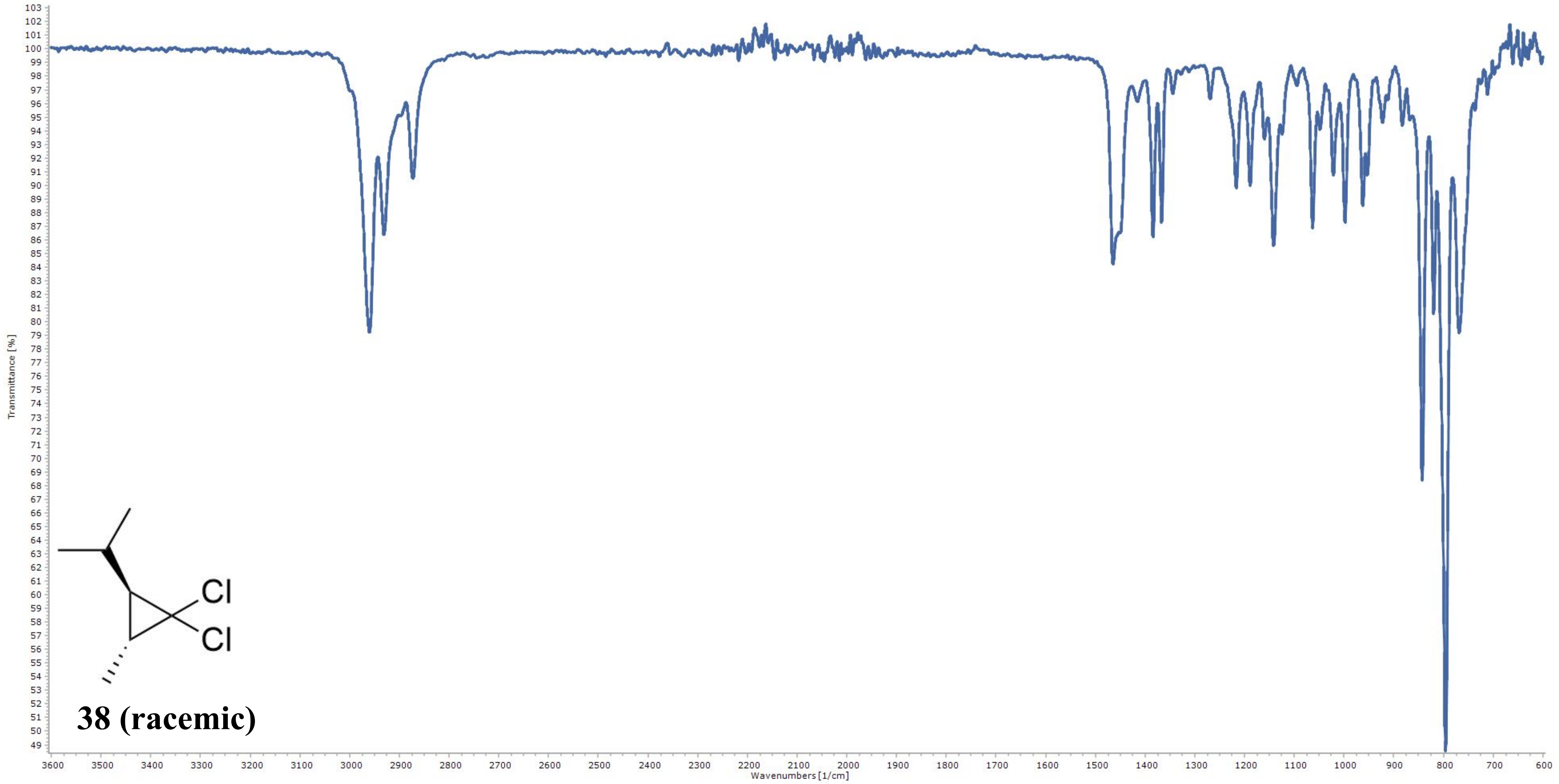

38 (racemic)

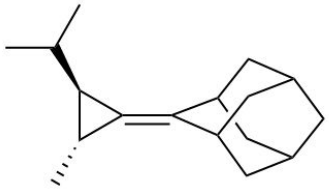

36 (racemic)

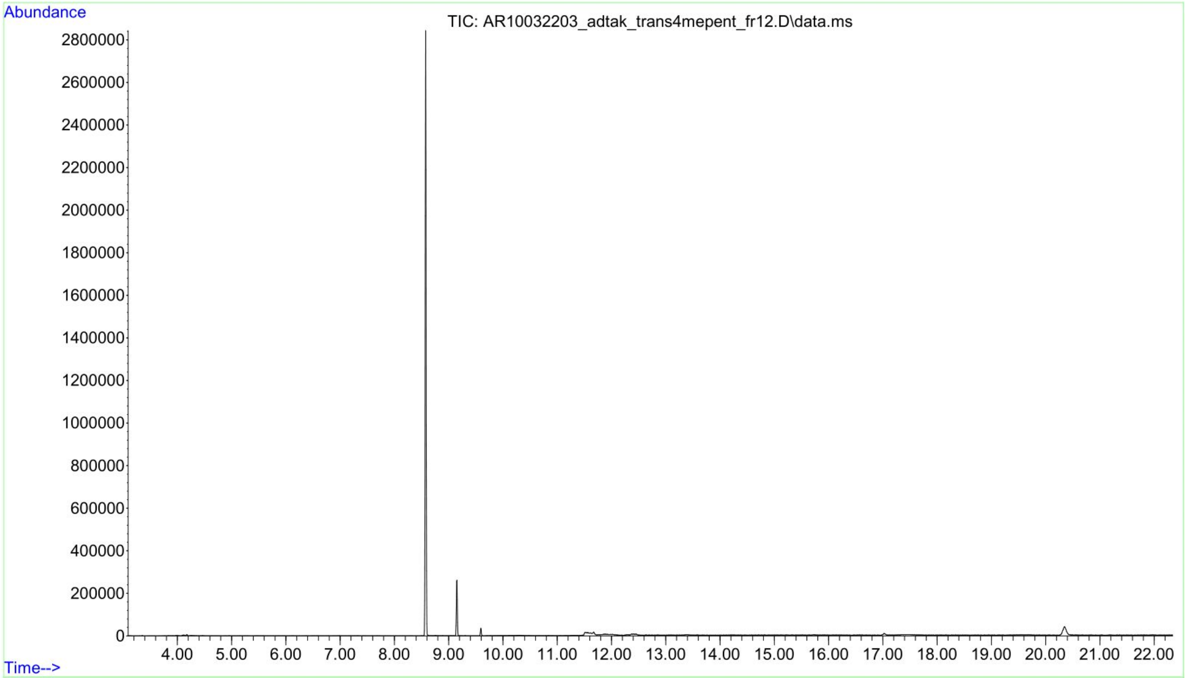

Abundance
TIC: AR10032203_adtak_trans4mepent_fr12.D\data.ms
2800000
2600000
2400000
2200000
2000000
1800000
1600000
1400000
1200000
1000000
800000
600000
400000
200000
0
4.00 5.00 6.00 7.00 8.00 9.00 10.00 11.00 12.00 13.00 14.00 15.00 16.00 17.00 18.00 19.00 20.00 21.00 22.00
Time-->

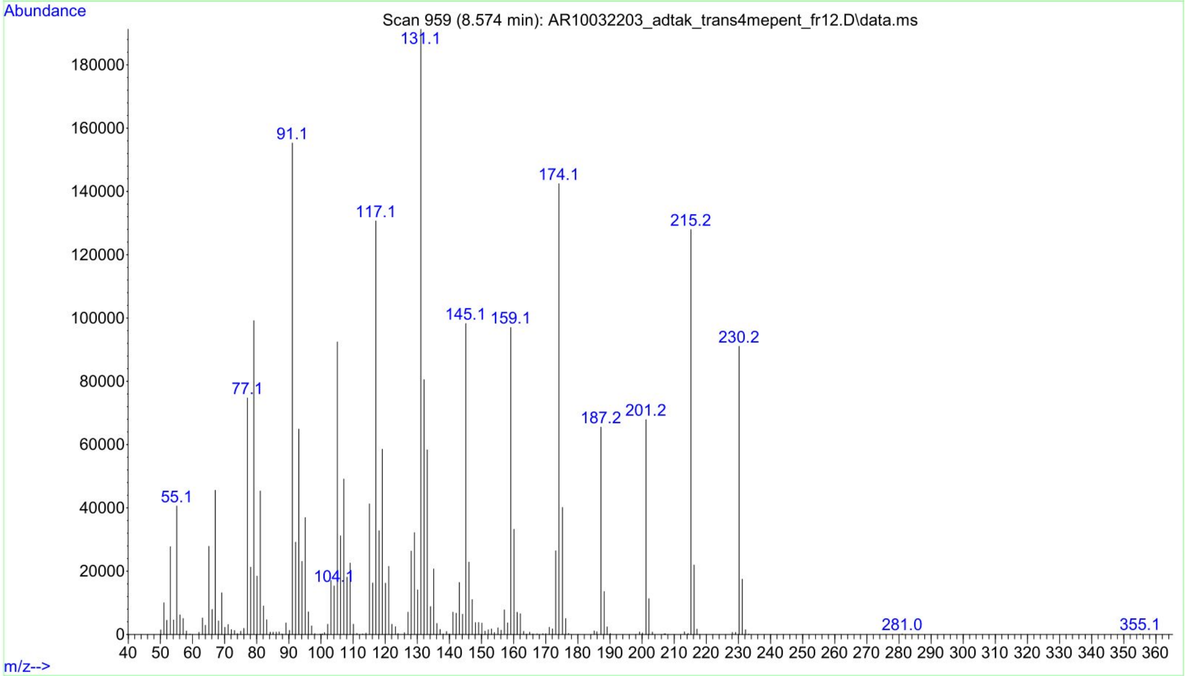

Abundance
Scan 959 (8.574 min): AR10032203_adtak_trans4mepent_fr12.D\data.ms
131.1
91.1
174.1
117.1
215.2
145.1
159.1
230.2
77.1
187.2
201.2
55.1
104.1
281.0
355.1
180000
160000
140000
120000
100000
80000
60000
40000
20000
0
40 50 60 70 80 90 100 110 120 130 140 150 160 170 180 190 200 210 220 230 240 250 260 270 280 290 300 310 320 330 340 350 360
m/z-->

^1H NMR, 500 Hz, $CDCl_3$

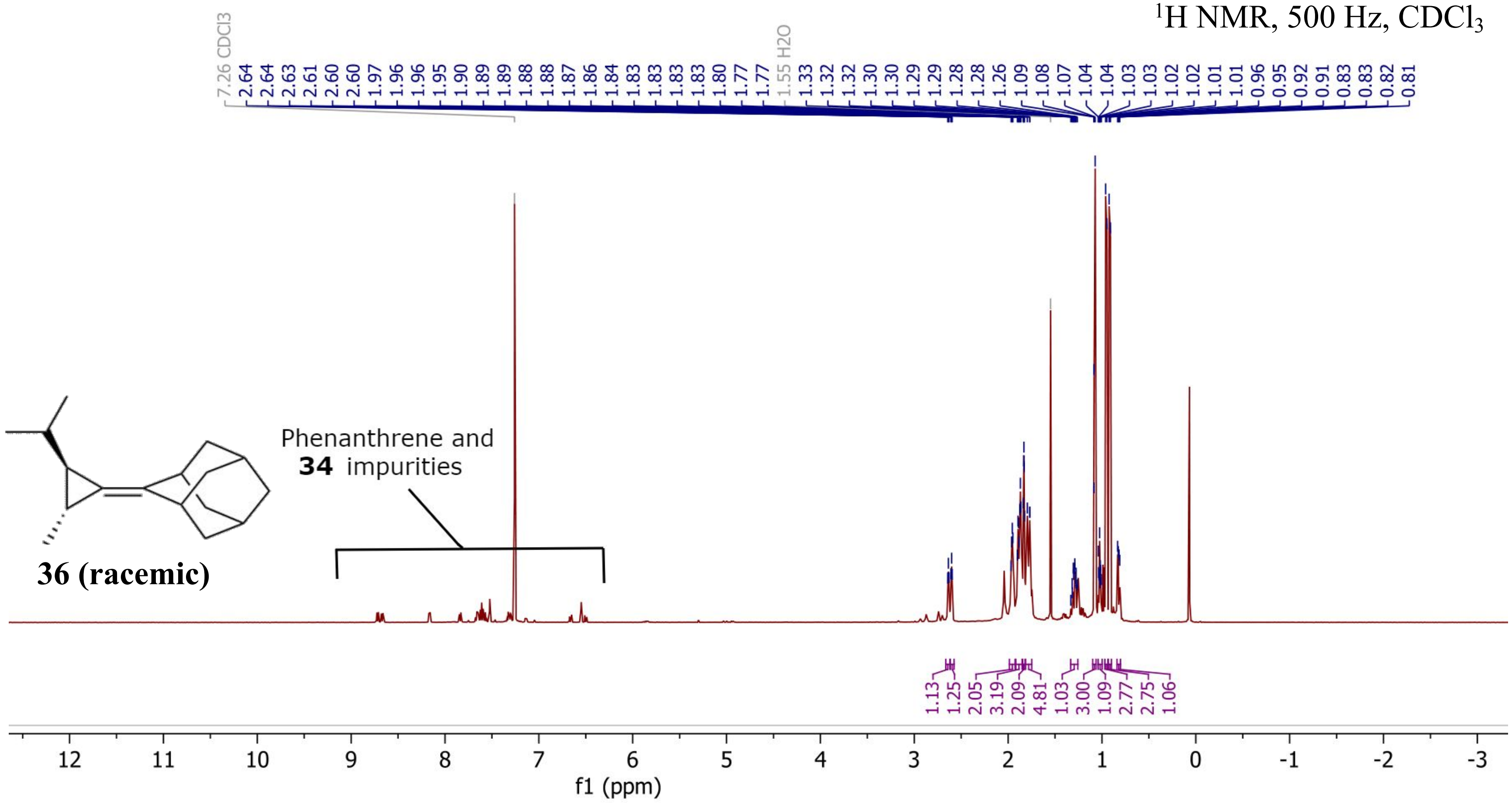

36 (racemic)

^{13}C NMR, 128 MHz, $CDCl_3$

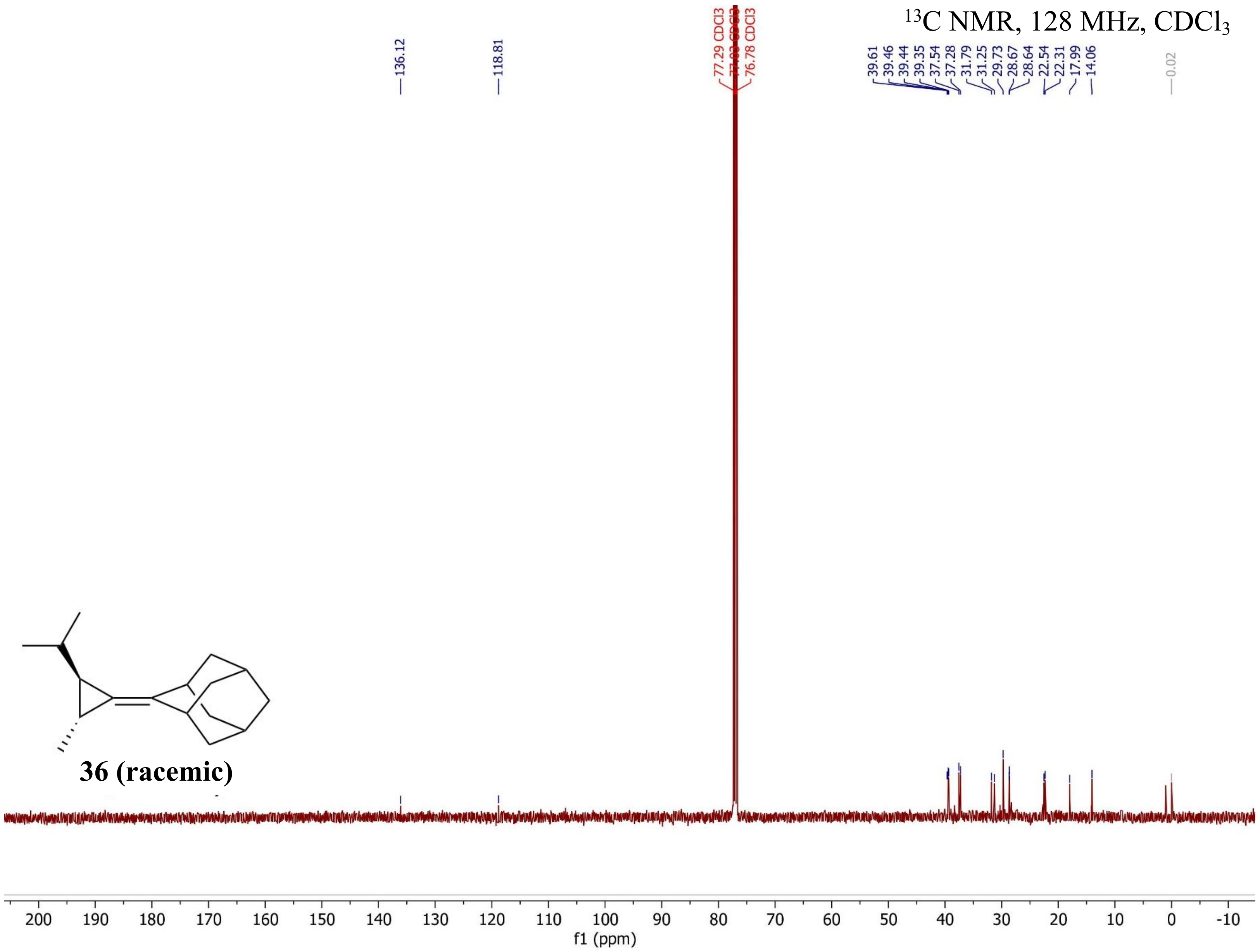

36 (racemic)

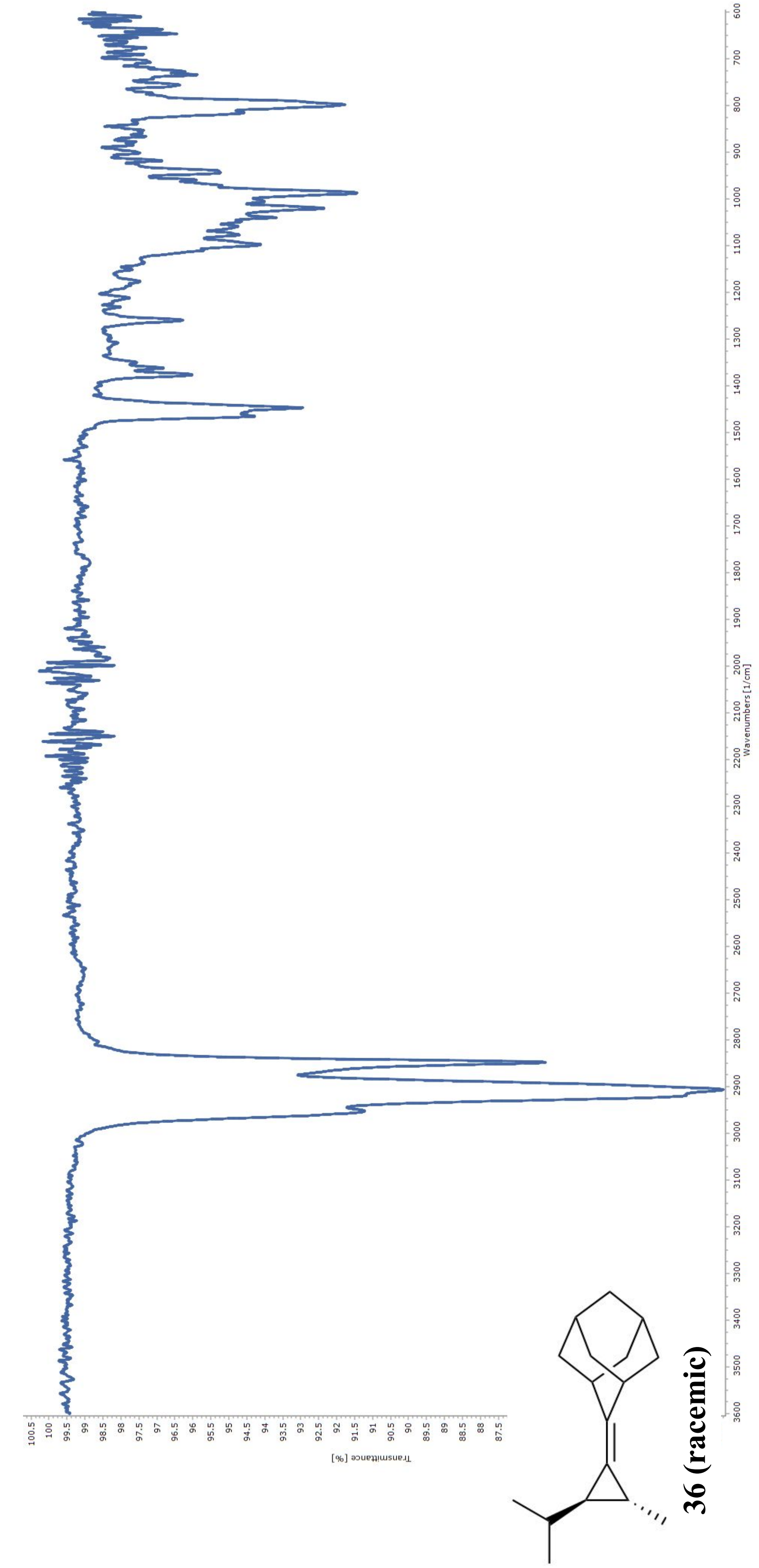

36 (racemic)

Photolysis Ai, t=3 hrs

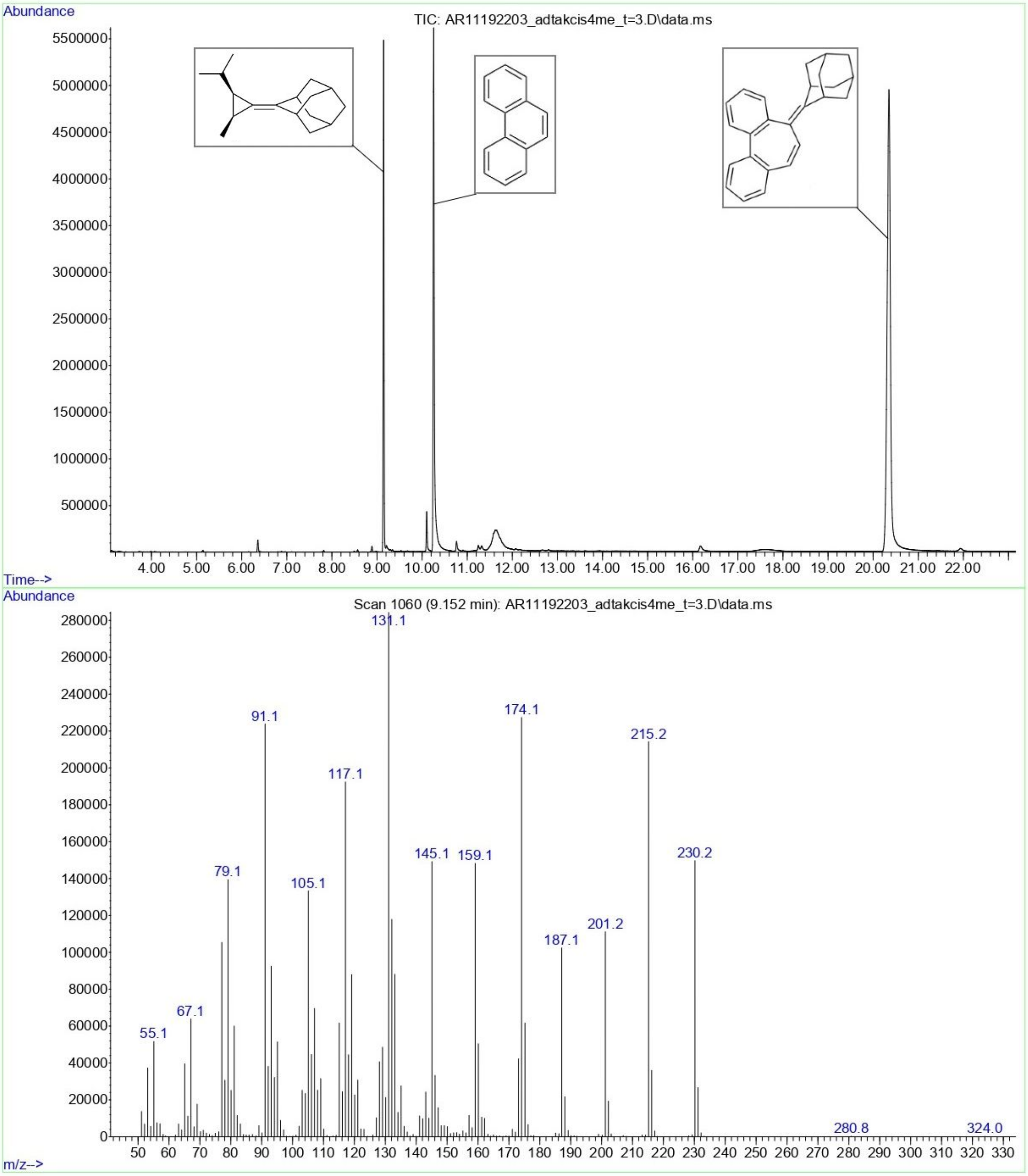

Photolysis Aii, t=1.5 hrs

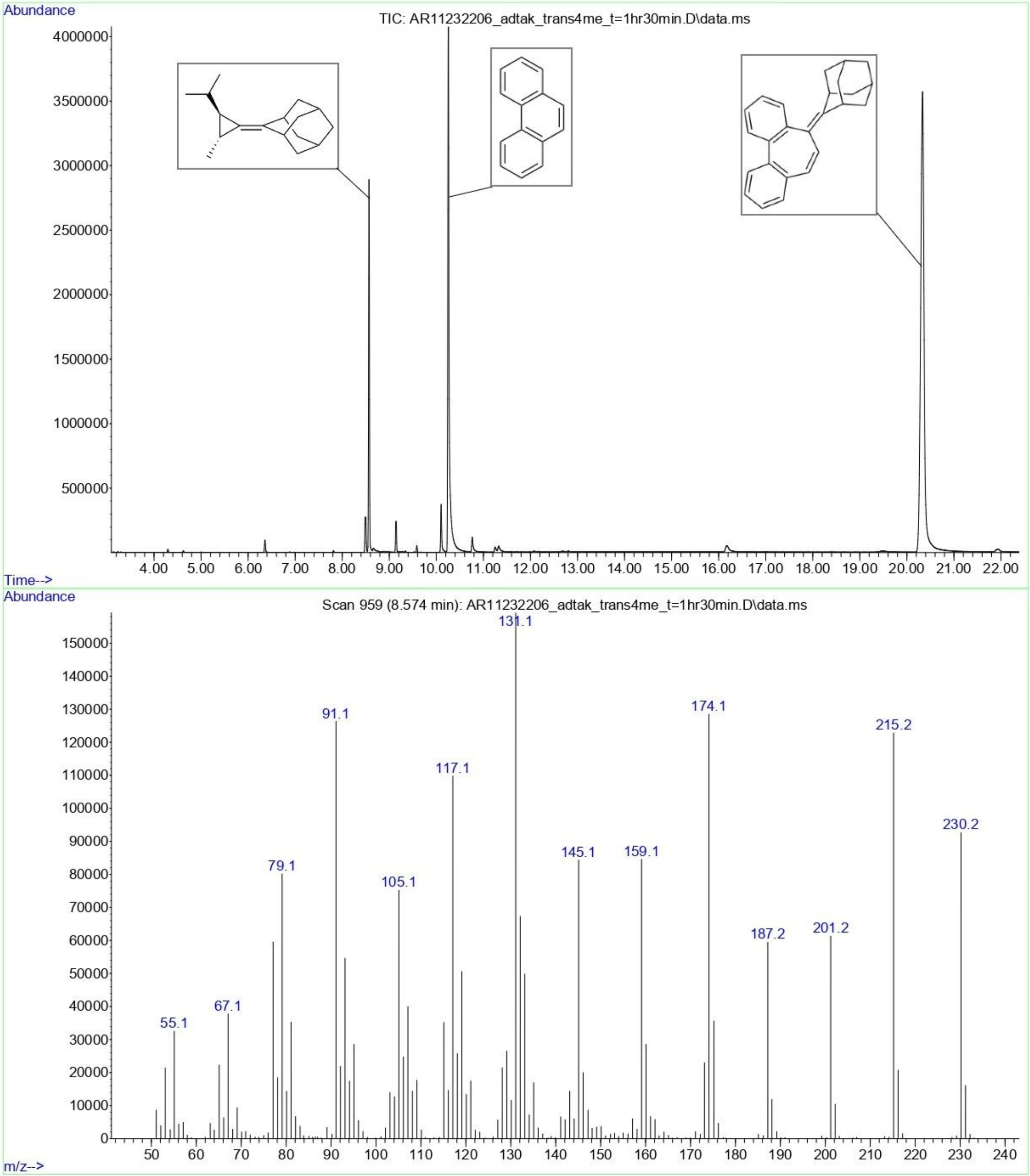

Photolysis C, t=20 hours

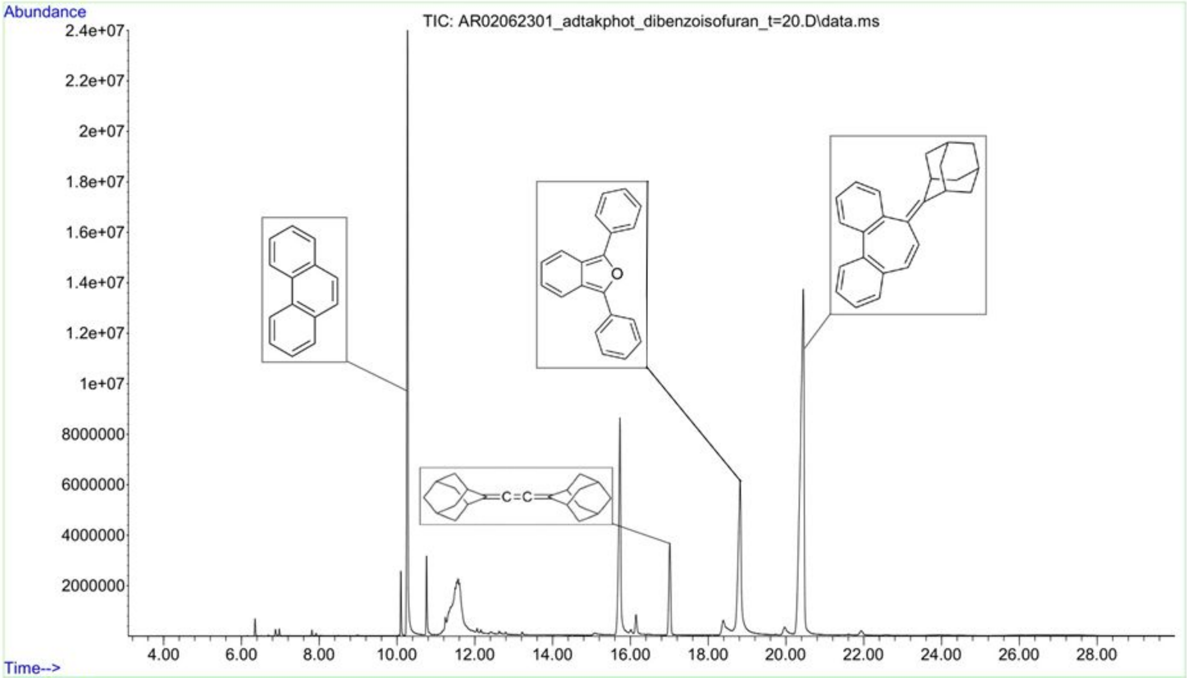

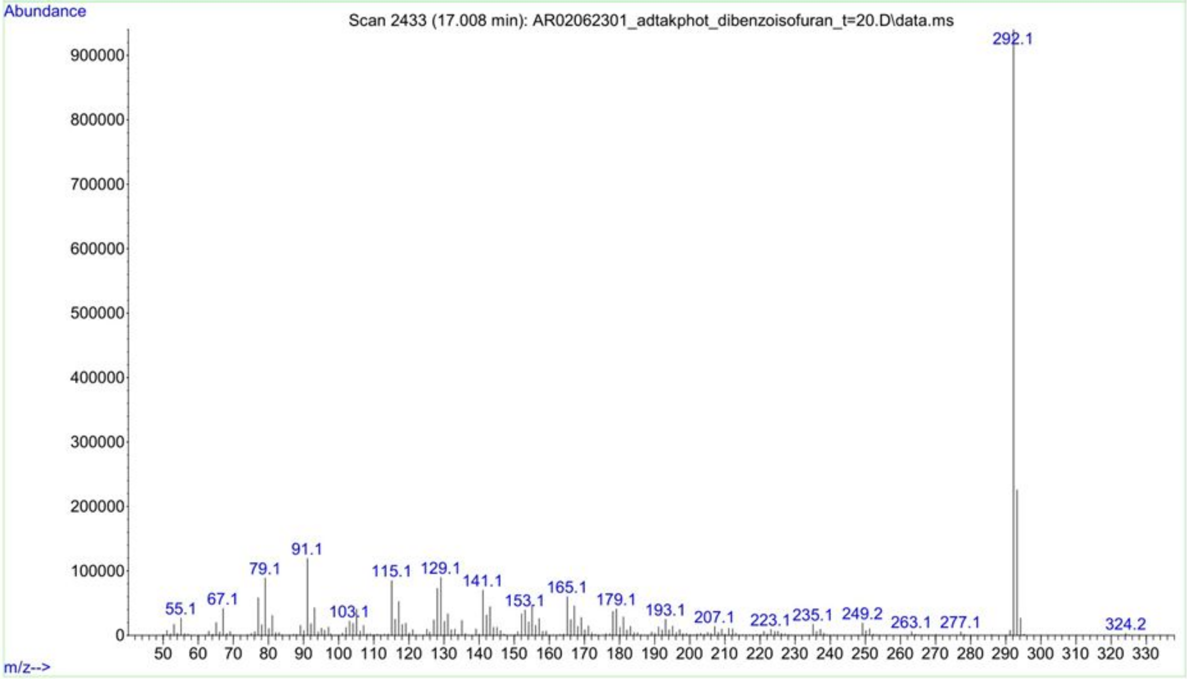

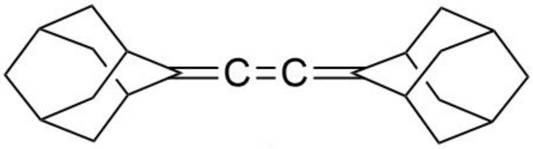

9

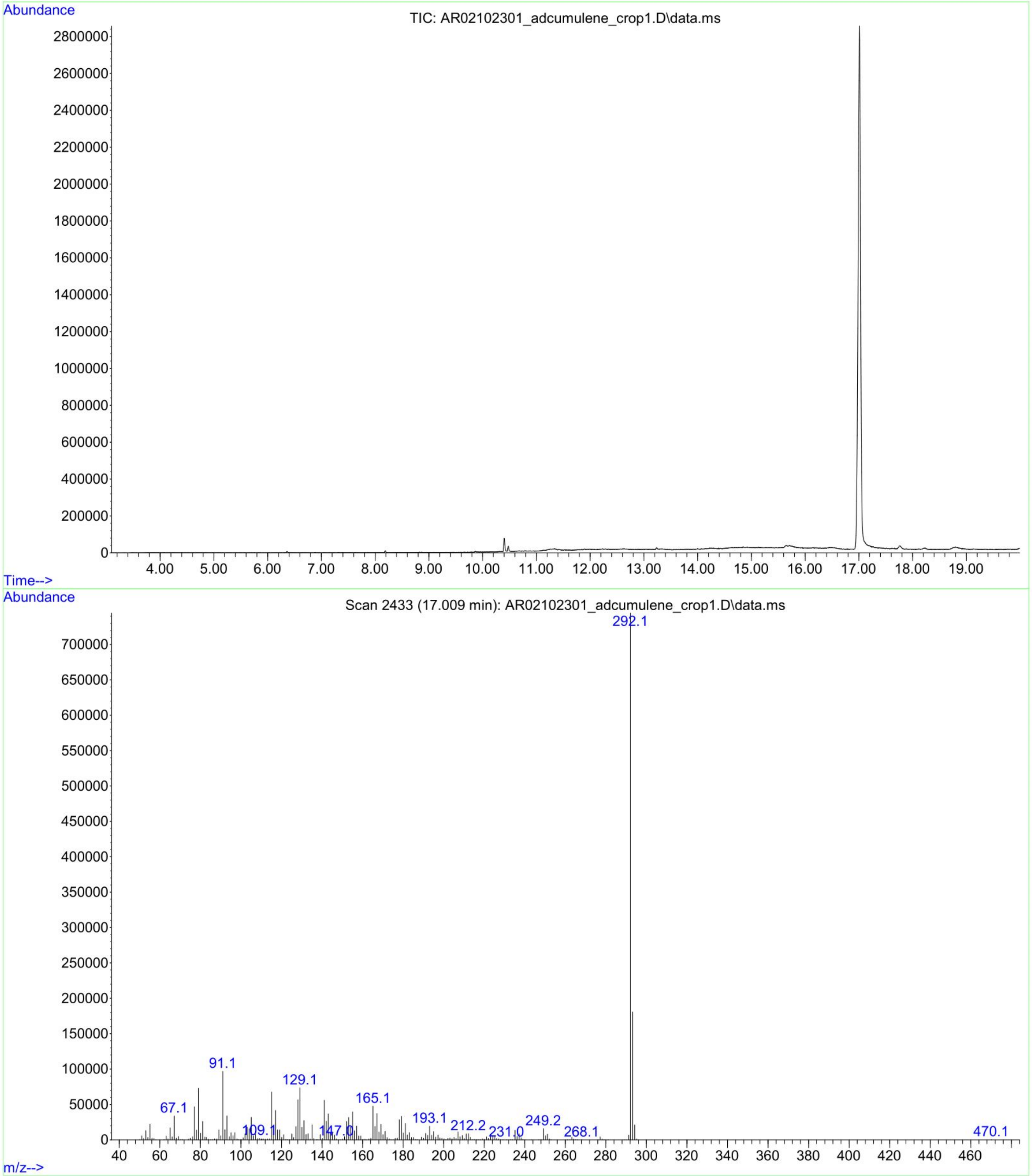
Abundance
TIC: AR02102301_adcumulene_crop1.D\data.ms
Time-->
Abundance
Scan 2433 (17.009 min): AR02102301_adcumulene_crop1.D\data.ms
292.1
91.1
129.1
67.1
165.1
193.1
109.1
147.0
212.2
231.0
249.2
268.1
470.1
m/z-->

Adamantylidene System

Optimized energies, coordinates, and frequencies for B2PLYP/def2-TZVP calculations:

1 (singlet)

INNER ENERGY

The inner energy is: U= E(el) + E(ZPE) + E(vib) + E(rot) + E(trans)
E(el) - is the total energy from the electronic structure calculation
= E(kin-el) + E(nuc-el) + E(el-el) + E(nuc-nuc)
E(ZPE) - the the zero temperature vibrational energy from the frequency calculation
E(vib) - the the finite temperature correction to E(ZPE) due to population
of excited vibrational states
E(rot) - is the rotational thermal energy
E(trans)- is the translational thermal energy

Summary of contributions to the inner energy U:
Electronic energy ... -427.24897996 Eh
Zero point energy ... 0.22408545 Eh 140.62 kcal/mol
Thermal vibrational correction ... 0.00542441 Eh 3.40 kcal/mol
Thermal rotational correction ... 0.00141627 Eh 0.89 kcal/mol
Thermal translational correction ... 0.00141627 Eh 0.89 kcal/mol
----
Total thermal energy -427.01663756 Eh

Summary of corrections to the electronic energy:
(perhaps to be used in another calculation)
Total thermal correction 0.00825695 Eh 5.18 kcal/mol
Non-thermal (ZPE) correction 0.22408545 Eh 140.62 kcal/mol

Total correction 0.23234240 Eh 145.80 kcal/mol

CARTESIAN COORDINATES (ANGSTROEM)

C	-1.972315	1.668279	0.184108
C	-1.435523	2.490974	-0.984007
C	-0.392087	1.632598	-1.714513
C	0.725974	1.232108	-0.743013
H	0.022623	2.207946	-2.547342
H	-0.870620	0.745100	-2.133749
C	-0.894449	1.243664	1.177691
C	0.139408	0.406731	0.409878
H	-0.329737	-0.502730	0.028891

H	0.934359	0.104283	1.097784
C	1.390218	2.496144	-0.180220
H	1.468400	0.632152	-1.274818
C	-0.237930	2.520337	1.724090
H	-1.334506	0.661684	1.987220
C	-0.769940	3.746190	-0.400255
H	-2.246758	2.765235	-1.658104
C	0.349401	3.340458	0.567726
H	-1.516186	4.356802	0.112240
H	-0.359808	4.344955	-1.218720
H	0.553024	2.241469	2.426390
H	-0.974542	3.109002	2.274807
H	0.824082	4.239634	0.967862
H	1.831459	3.080085	-0.992844
H	2.204424	2.221054	0.496162
C	-3.222071	1.359595	0.318508

SCF STABILITY ANALYSIS RESULT

RHF/RKS->UHF/UKS - triplet - external

Root	Eigenvalue (au)
0	0.016155
1	0.060221
2	0.093342

Stability Analysis indicates a stable HF/KS wave function.

No imaginary frequency.

1 (triplet)

INNER ENERGY

The inner energy is: U= E(el) + E(ZPE) + E(vib) + E(rot) + E(trans)
E(el) - is the total energy from the electronic structure calculation
= E(kin-el) + E(nuc-el) + E(el-el) + E(nuc-nuc)
E(ZPE) - the the zero temperature vibrational energy from the frequency calculation
E(vib) - the the finite temperature correction to E(ZPE) due to population
of excited vibrational states
E(rot) - is the rotational thermal energy
E(trans)- is the translational thermal energy

Summary of contributions to the inner energy U:
Electronic energy ... -427.17182948 Eh
Zero point energy ... 0.22256556 Eh 139.66 kcal/mol
Thermal vibrational correction ... 0.00538628 Eh 3.38 kcal/mol
Thermal rotational correction ... 0.00141627 Eh 0.89 kcal/mol
Thermal translational correction ... 0.00141627 Eh 0.89 kcal/mol
----
Total thermal energy -426.94104510 Eh

Summary of corrections to the electronic energy:
(perhaps to be used in another calculation)
Total thermal correction 0.00821882 Eh 5.16 kcal/mol
Non-thermal (ZPE) correction 0.22256556 Eh 139.66 kcal/mol

Total correction 0.23078439 Eh 144.82 kcal/mol

CARTESIAN COORDINATES (ANGSTROEM)

C	-1.975880	1.692673	0.199039
C	-1.442393	2.481958	-0.958608
C	-0.387735	1.634745	-1.707208
C	0.732287	1.235697	-0.742506
H	0.010973	2.221636	-2.539229
H	-0.862874	0.747520	-2.131381
C	-0.911770	1.258631	1.161789
C	0.141041	0.414811	0.406923
H	-0.323309	-0.497804	0.027021
H	0.923479	0.115579	1.109732
C	1.403152	2.496254	-0.183409
H	1.470671	0.631352	-1.275758
C	-0.226996	2.532154	1.718729
H	-1.360793	0.679844	1.968401
C	-0.755685	3.750148	-0.392045
H	-2.260069	2.753538	-1.625646
C	0.367504	3.343923	0.565190
H	-1.490887	4.372904	0.122478
H	-0.361526	4.334284	-1.228036
H	0.552756	2.227701	2.422115
H	-0.951630	3.131200	2.274750
H	0.845539	4.242509	0.964069
H	1.844262	3.077979	-0.997463
H	2.217334	2.218884	0.491787
C	-3.310552	1.245630	0.275034

SCF STABILITY ANALYSIS RESULT

UHF/UKS->UHF/UKS - singlet - internal

Root	Eigenvalue (au)
0	0.014666
1	0.081810
2	0.132135

Stability Analysis indicates a stable HF/KS wave function.

No imaginary frequency.

10

INNER ENERGY

The inner energy is: U= E(el) + E(ZPE) + E(vib) + E(rot) + E(trans)
E(el) - is the total energy from the electronic structure calculation
= E(kin-el) + E(nuc-el) + E(el-el) + E(nuc-nuc)
E(ZPE) - the the zero temperature vibrational energy from the frequency calculation
E(vib) - the the finite temperature correction to E(ZPE) due to population
of excited vibrational states
E(rot) - is the rotational thermal energy
E(trans)- is the translational thermal energy

Summary of contributions to the inner energy U:
Electronic energy ... -427.26237067 Eh
Zero point energy ... 0.22525040 Eh 141.35 kcal/mol
Thermal vibrational correction ... 0.00494055 Eh 3.10 kcal/mol
Thermal rotational correction ... 0.00141627 Eh 0.89 kcal/mol
Thermal translational correction ... 0.00141627 Eh 0.89 kcal/mol
----
Total thermal energy -427.02934718 Eh

Summary of corrections to the electronic energy:
(perhaps to be used in another calculation)
Total thermal correction 0.00777310 Eh 4.88 kcal/mol
Non-thermal (ZPE) correction 0.22525040 Eh 141.35 kcal/mol

Total correction 0.23302350 Eh 146.22 kcal/mol

CARTESIAN COORDINATES (ANGSTROEM)

C	-1.250581	3.119660	-0.534054
C	-0.797769	4.258305	-1.359943
C	0.553709	3.805207	-1.960530
C	-0.767995	2.524733	0.410505
C	0.493240	2.666344	1.167326
C	1.613637	2.498801	0.114340
C	1.698158	3.645372	-0.926415
H	0.882773	4.562118	-2.680510
H	0.405997	2.877774	-2.516361
H	1.495881	1.535332	-0.384487
H	2.576491	2.474900	0.635741
C	1.861440	4.967673	-0.177480
H	2.602445	3.451343	-1.510499
C	0.471582	4.112814	1.714649
H	0.629597	1.954714	1.982254
C	-0.588325	5.419706	-0.360285
H	-1.482809	4.559879	-2.152980
C	0.587473	5.215103	0.629899
H	-1.519054	5.599580	0.180365
H	-0.366603	6.329115	-0.928951
H	1.326943	4.240582	2.386638
H	-0.429631	4.256099	2.313157
H	0.686590	6.158986	1.173769
H	2.030782	5.786508	-0.882336
H	2.730529	4.925600	0.485031

SCF STABILITY ANALYSIS RESULT

RHF/RKS->UHF/UKS - triplet - external

Root	Eigenvalue (au)
0	0.002121
1	0.060605
2	0.119524

Stability Analysis indicates a stable HF/KS wave function.

No imaginary frequency.

TS (1 to 10)

INNER ENERGY

The inner energy is: U= E(el) + E(ZPE) + E(vib) + E(rot) + E(trans)
E(el) - is the total energy from the electronic structure calculation
= E(kin-el) + E(nuc-el) + E(el-el) + E(nuc-nuc)
E(ZPE) - the the zero temperature vibrational energy from the frequency calculation
E(vib) - the the finite temperature correction to E(ZPE) due to population
of excited vibrational states
E(rot) - is the rotational thermal energy
E(trans)- is the translational thermal energy

Summary of contributions to the inner energy U:
Electronic energy ... -427.22815022 Eh
Zero point energy ... 0.22315637 Eh 140.03 kcal/mol
Thermal vibrational correction ... 0.00488493 Eh 3.07 kcal/mol
Thermal rotational correction ... 0.00141627 Eh 0.89 kcal/mol
Thermal translational correction ... 0.00141627 Eh 0.89 kcal/mol
----
Total thermal energy -426.99727638 Eh

Summary of corrections to the electronic energy:
(perhaps to be used in another calculation)
Total thermal correction 0.00771747 Eh 4.84 kcal/mol
Non-thermal (ZPE) correction 0.22315637 Eh 140.03 kcal/mol

Total correction 0.23087385 Eh 144.88 kcal/mol

CARTESIAN COORDINATES (ANGSTROEM)

C	-1.749513	-0.606228	0.472531
C	-1.239891	0.468806	-1.107535
C	-0.218756	-0.502076	-1.695181
C	0.911435	-0.880154	-0.723117
H	0.233514	-0.011520	-2.564295
H	-0.733833	-1.390819	-2.062300
C	-0.589847	-0.916826	1.305534
C	0.417173	-1.719328	0.462412
H	-0.048234	-2.642684	0.113788
H	1.268356	-1.992112	1.091910
C	1.571233	0.399897	-0.195137
H	1.648881	-1.467795	-1.275504
C	0.036978	0.406102	1.781296
H	-0.930759	-1.507594	2.159138

C	-0.596535	1.638844	-0.367771
H	-1.874627	0.847940	-1.899176
C	0.538169	1.223808	0.583543
H	-1.367209	2.201195	0.161157
H	-0.174120	2.307998	-1.125721
H	0.877081	0.183046	2.444312
H	-0.697248	0.974612	2.354445
H	1.010448	2.133250	0.963234
H	1.969708	0.987490	-1.026887
H	2.415201	0.146814	0.452595
C	-2.677604	-0.282661	-0.303270

SCF STABILITY ANALYSIS RESULT

RHF/RKS->UHF/UKS - triplet - external

Root	Eigenvalue (au)
0	0.104826
1	0.128836
2	0.147987

Stability Analysis indicates a stable HF/KS wave function.

One imaginary frequency (-437.06 cm^{-1}).

Optimized energies, coordinates, and frequencies for B3LYP/def2-TZVP calculations:

1 (singlet)

INNER ENERGY

The inner energy is: U= E(el) + E(ZPE) + E(vib) + E(rot) + E(trans)
E(el) - is the total energy from the electronic structure calculation
= E(kin-el) + E(nuc-el) + E(el-el) + E(nuc-nuc)
E(ZPE) - the the zero temperature vibrational energy from the frequency calculation
E(vib) - the the finite temperature correction to E(ZPE) due to population
of excited vibrational states
E(rot) - is the rotational thermal energy
E(trans)- is the translational thermal energy

Summary of contributions to the inner energy U:
Electronic energy ... -427.39404457 Eh
Zero point energy ... 0.22294457 Eh 139.90 kcal/mol

Thermal vibrational correction ... 0.00536901 Eh 3.37 kcal/mol
Thermal rotational correction ... 0.00141627 Eh 0.89 kcal/mol
Thermal translational correction ... 0.00141627 Eh 0.89 kcal/mol
----
Total thermal energy -427.16289844 Eh

Summary of corrections to the electronic energy:
(perhaps to be used in another calculation)
Total thermal correction 0.00820155 Eh 5.15 kcal/mol
Non-thermal (ZPE) correction 0.22294457 Eh 139.90 kcal/mol

Total correction 0.23114612 Eh 145.05 kcal/mol

CARTESIAN COORDINATES (ANGSTROEM)

C	-1.976796	1.667498	0.185463
C	-1.437336	2.490291	-0.984363
C	-0.392027	1.631864	-1.716385
C	0.727759	1.230880	-0.744185
H	0.021786	2.208710	-2.549017
H	-0.869208	0.744262	-2.137603
C	-0.895545	1.242787	1.178693
C	0.139382	0.404465	0.409792
H	-0.329999	-0.505005	0.028499
H	0.934567	0.100190	1.097019
C	1.392710	2.496627	-0.180189
H	1.470565	0.631022	-1.276128
C	-0.238687	2.521055	1.726024
H	-1.333894	0.660264	1.988671
C	-0.770894	3.747670	-0.401064
H	-2.247810	2.764252	-1.659331
C	0.349876	3.341959	0.568529
H	-1.516941	4.360047	0.110226
H	-0.360129	4.345781	-1.220071
H	0.551897	2.242146	2.429304
H	-0.975119	3.109670	2.277502
H	0.824572	4.241547	0.968535
H	1.833910	3.081343	-0.992659
H	2.207217	2.222250	0.496548
C	-3.222956	1.362175	0.321963

SCF STABILITY ANALYSIS RESULT

RHF/RKS->UHF/UKS - triplet - external

Root	Eigenvalue (au)
0	0.031824
1	0.067109
2	0.130916

Stability Analysis indicates a stable HF/KS wave function.

No imaginary frequency.

10

INNER ENERGY

The inner energy is: U= E(el) + E(ZPE) + E(vib) + E(rot) + E(trans)
E(el)   - is the total energy from the electronic structure calculation
= E(kin-el) + E(nuc-el) + E(el-el) + E(nuc-nuc)
E(ZPE) - the the zero temperature vibrational energy from the frequency calculation
E(vib) - the the finite temperature correction to E(ZPE) due to population
of excited vibrational states
E(rot) - is the rotational thermal energy
E(trans)- is the translational thermal energy

Summary of contributions to the inner energy U:
Electronic energy ... -427.40289902 Eh
Zero point energy ... 0.22417664 Eh 140.67 kcal/mol
Thermal vibrational correction ... 0.00496446 Eh 3.12 kcal/mol
Thermal rotational correction ... 0.00141627 Eh 0.89 kcal/mol
Thermal translational correction ... 0.00141627 Eh 0.89 kcal/mol
----
Total thermal energy -427.17092538 Eh

Summary of corrections to the electronic energy:
(perhaps to be used in another calculation)
Total thermal correction 0.00779700 Eh 4.89 kcal/mol
Non-thermal (ZPE) correction 0.22417664 Eh 140.67 kcal/mol

Total correction 0.23197364 Eh 145.57 kcal/mol

CARTESIAN COORDINATES (ANGSTROEM)

C	-1.249042	3.115561	-0.530359
C	-0.799444	4.255130	-1.358506
C	0.554632	3.804921	-1.962391
C	-0.770238	2.525628	0.407328
C	0.490925	2.666582	1.166404
C	1.614348	2.496885	0.113002
C	1.702483	3.645962	-0.928168
H	0.879330	4.563187	-2.683163
H	0.408422	2.877085	-2.518368
H	1.494023	1.534441	-0.387428
H	2.576662	2.467690	0.635771
C	1.865769	4.969408	-0.177199
H	2.606840	3.451919	-1.512397
C	0.470422	4.115279	1.715587
H	0.627150	1.955237	1.982481
C	-0.590105	5.420782	-0.359046
H	-1.486075	4.555606	-2.151336
C	0.590113	5.219125	0.630282
H	-1.519658	5.597401	0.185016
H	-0.375093	6.330456	-0.930319
H	1.323733	4.239676	2.391302
H	-0.432575	4.260187	2.311219
H	0.689771	6.163546	1.173489
H	2.038166	5.788798	-0.881209
H	2.733940	4.925755	0.486849

SCF STABILITY ANALYSIS RESULT

RHF/RKS->UHF/UKS - triplet - external

Root	Eigenvalue (au)
0	0.047416
1	0.090432
2	0.153106

Stability Analysis indicates a stable HF/KS wave function.

No imaginary frequency.

TS (1 to 10)

INNER ENERGY

The inner energy is: U= E(el) + E(ZPE) + E(vib) + E(rot) + E(trans)
E(el) - is the total energy from the electronic structure calculation
= E(kin-el) + E(nuc-el) + E(el-el) + E(nuc-nuc)
E(ZPE) - the the zero temperature vibrational energy from the frequency calculation
E(vib) - the the finite temperature correction to E(ZPE) due to population
of excited vibrational states
E(rot) - is the rotational thermal energy
E(trans)- is the translational thermal energy

Summary of contributions to the inner energy U:
Electronic energy ... -427.37087916 Eh
Zero point energy ... 0.22183435 Eh 139.20 kcal/mol
Thermal vibrational correction ... 0.00492688 Eh 3.09 kcal/mol
Thermal rotational correction ... 0.00141627 Eh 0.89 kcal/mol
Thermal translational correction ... 0.00141627 Eh 0.89 kcal/mol
----
Total thermal energy -427.14128538 Eh

Summary of corrections to the electronic energy:
(perhaps to be used in another calculation)
Total thermal correction 0.00775943 Eh 4.87 kcal/mol
Non-thermal (ZPE) correction 0.22183435 Eh 139.20 kcal/mol

Total correction 0.22959378 Eh 144.07 kcal/mol

CARTESIAN COORDINATES (ANGSTROEM)

C	-1.755411	-0.607381	0.472364
C	-1.237017	0.465713	-1.106748
C	-0.212397	-0.499625	-1.698974
C	0.917524	-0.878429	-0.724129
H	0.239439	-0.003691	-2.565949
H	-0.723800	-1.388577	-2.071043
C	-0.593996	-0.916506	1.303014
C	0.415968	-1.720249	0.459220
H	-0.052531	-2.640169	0.104795
H	1.263523	-2.000994	1.090554
C	1.575093	0.404101	-0.193781
H	1.657353	-1.465759	-1.274182
C	0.036419	0.406554	1.783435
H	-0.933573	-1.508616	2.156823
C	-0.599473	1.639029	-0.366229
H	-1.878173	0.839972	-1.895086
C	0.538312	1.226833	0.585669

H	-1.372408	2.196322	0.165215
H	-0.181838	2.314012	-1.122280
H	0.876737	0.179019	2.445042
H	-0.695917	0.974670	2.359925
H	1.008660	2.137428	0.965723
H	1.970914	0.993533	-1.025927
H	2.420951	0.155015	0.453544
C	-2.684357	-0.302202	-0.300995

SCF STABILITY ANALYSIS RESULT

RHF/RKS->UHF/UKS - triplet - external

Root	Eigenvalue (au)
0	0.137765
1	0.144785
2	0.159118

Stability Analysis indicates a stable HF/KS wave function.

One imaginary frequency (-441.78 cm^{-1}).

Optimized energies, coordinates, and frequencies for PBE0/def2-TZVP calculations:

1 (singlet)

INNER ENERGY

The inner energy is: U= E(el) + E(ZPE) + E(vib) + E(rot) + E(trans)
E(el) - is the total energy from the electronic structure calculation
= E(kin-el) + E(nuc-el) + E(el-el) + E(nuc-nuc)
E(ZPE) - the the zero temperature vibrational energy from the frequency calculation
E(vib) - the the finite temperature correction to E(ZPE) due to population
of excited vibrational states
E(rot) - is the rotational thermal energy
E(trans)- is the translational thermal energy

Summary of contributions to the inner energy U:
Electronic energy ... -427.14399140 Eh
Zero point energy ... 0.22374444 Eh 140.40 kcal/mol
Thermal vibrational correction ... 0.00553601 Eh 3.47 kcal/mol
Thermal rotational correction ... 0.00141627 Eh 0.89 kcal/mol
Thermal translational correction ... 0.00141627 Eh 0.89 kcal/mol

----
Total thermal energy -426.91187840 Eh

Summary of corrections to the electronic energy:
(perhaps to be used in another calculation)
Total thermal correction 0.00836856 Eh 5.25 kcal/mol
Non-thermal (ZPE) correction 0.22374444 Eh 140.40 kcal/mol

Total correction 0.23211300 Eh 145.65 kcal/mol

CARTESIAN COORDINATES (ANGSTROEM)

C	-1.965920	1.670595	0.184442
C	-1.431282	2.488862	-0.980185
C	-0.391511	1.634487	-1.707779
C	0.722025	1.235071	-0.740322
H	0.023662	2.212549	-2.540630
H	-0.869124	0.746309	-2.130629
C	-0.891796	1.246910	1.173641
C	0.136891	0.413002	0.407867
H	-0.333273	-0.497638	0.026630
H	0.933635	0.108422	1.095245
C	1.383936	2.493953	-0.179400
H	1.465291	0.634019	-1.272945
C	-0.238658	2.518839	1.717136
H	-1.332025	0.663763	1.984383
C	-0.768028	3.739090	-0.399403
H	-2.244018	2.762600	-1.655197
C	0.346713	3.335110	0.565324
H	-1.514620	4.352899	0.112163
H	-0.356291	4.337474	-1.219493
H	0.553061	2.239569	2.421078
H	-0.975652	3.108198	2.269646
H	0.821966	4.235789	0.965733
H	1.825588	3.079357	-0.992846
H	2.199376	2.219257	0.498135
C	-3.213048	1.365263	0.323173

SCF STABILITY ANALYSIS RESULT

RHF/RKS->UHF/UKS - triplet - external

Root	Eigenvalue (au)
0	0.020801
1	0.061421
2	0.122025

Stability Analysis indicates a stable HF/KS wave function.

No imaginary frequency.

10

INNER ENERGY

The inner energy is: U= E(el) + E(ZPE) + E(vib) + E(rot) + E(trans)
E(el) - is the total energy from the electronic structure calculation
= E(kin-el) + E(nuc-el) + E(el-el) + E(nuc-nuc)
E(ZPE) - the the zero temperature vibrational energy from the frequency calculation
E(vib) - the the finite temperature correction to E(ZPE) due to population
of excited vibrational states
E(rot) - is the rotational thermal energy
E(trans)- is the translational thermal energy

Summary of contributions to the inner energy U:

Electronic energy	...	-427.15362598 Eh	
Zero point energy	...	0.22517324 Eh	141.30 kcal/mol
Thermal vibrational correction	...	0.00496481 Eh	3.12 kcal/mol
Thermal rotational correction	...	0.00141627 Eh	0.89 kcal/mol
Thermal translational correction	...	0.00141627 Eh	0.89 kcal/mol

----
Total thermal energy -426.92065539 Eh

Summary of corrections to the electronic energy:
(perhaps to be used in another calculation)

Total thermal correction	0.00779735 Eh	4.89 kcal/mol
Non-thermal (ZPE) correction	0.22517324 Eh	141.30 kcal/mol

Total correction 0.23297059 Eh 146.19 kcal/mol

CARTESIAN COORDINATES (ANGSTROEM)

C	-1.234957	3.126115	-0.530883
C	-0.793649	4.260181	-1.359466
C	0.552867	3.807140	-1.954665

C	-0.756148	2.536246	0.406734
C	0.497469	2.670828	1.166843
C	1.609219	2.503151	0.114152
C	1.691656	3.644954	-0.923288
H	0.884438	4.565004	-2.674958
H	0.404314	2.879276	-2.512701
H	1.488519	1.538183	-0.384361
H	2.575856	2.476748	0.632108
C	1.855962	4.961962	-0.176778
H	2.596991	3.450148	-1.508516
C	0.471584	4.112691	1.707867
H	0.633673	1.958495	1.983982
C	-0.585666	5.414049	-0.360185
H	-1.481340	4.560757	-2.153308
C	0.585881	5.208848	0.625924
H	-1.517767	5.590776	0.182268
H	-0.366685	6.327269	-0.927063
H	1.326361	4.243893	2.382807
H	-0.432040	4.255356	2.305629
H	0.685224	6.154486	1.170025
H	2.027985	5.782217	-0.881907
H	2.724754	4.917476	0.488582

SCF STABILITY ANALYSIS RESULT

RHF/RKS->UHF/UKS - triplet - external

Root	Eigenvalue (au)
0	0.038311
1	0.085519
2	0.145746

Stability Analysis indicates a stable HF/KS wave function.

No imaginary frequency.

TS (1 to 10)

INNER ENERGY

The inner energy is: U= E(el) + E(ZPE) + E(vib) + E(rot) + E(trans)
E(el) - is the total energy from the electronic structure calculation
= E(kin-el) + E(nuc-el) + E(el-el) + E(nuc-nuc)

E(ZPE) - the the zero temperature vibrational energy from the frequency calculation
E(vib) - the the finite temperature correction to E(ZPE) due to population
of excited vibrational states
E(rot) - is the rotational thermal energy
E(trans)- is the translational thermal energy

Summary of contributions to the inner energy U:
Electronic energy ... -427.12354928 Eh
Zero point energy ... 0.22292875 Eh 139.89 kcal/mol
Thermal vibrational correction ... 0.00487551 Eh 3.06 kcal/mol
Thermal rotational correction ... 0.00141627 Eh 0.89 kcal/mol
Thermal translational correction ... 0.00141627 Eh 0.89 kcal/mol
----
Total thermal energy -426.89291247 Eh

Summary of corrections to the electronic energy:
(perhaps to be used in another calculation)
Total thermal correction 0.00770805 Eh 4.84 kcal/mol
Non-thermal (ZPE) correction 0.22292875 Eh 139.89 kcal/mol

Total correction 0.23063680 Eh 144.73 kcal/mol

CARTESIAN COORDINATES (ANGSTROEM)

C	-1.737259	-0.601566	0.468792
C	-1.244912	0.459007	-1.095432
C	-0.220690	-0.500860	-1.686585
C	0.907588	-0.875686	-0.720399
H	0.230523	-0.006301	-2.555756
H	-0.732712	-1.392124	-2.056735
C	-0.584275	-0.913524	1.301550
C	0.417927	-1.713232	0.460781
H	-0.049544	-2.637092	0.111423
H	1.270725	-1.988450	1.089696
C	1.562227	0.401704	-0.196222
H	1.646933	-1.462583	-1.274223
C	0.040227	0.403718	1.777699
H	-0.932208	-1.505632	2.153703
C	-0.601140	1.628328	-0.362864
H	-1.863432	0.847703	-1.899457
C	0.532500	1.219460	0.582692
H	-1.372403	2.191775	0.167607
H	-0.180215	2.298967	-1.122289
H	0.885131	0.178249	2.436245

H	-0.692336	0.970437	2.357635
H	1.003548	2.132090	0.960848
H	1.953692	0.992053	-1.031383
H	2.412393	0.153718	0.448169
C	-2.652289	-0.280158	-0.315495

SCF STABILITY ANALYSIS RESULT

RHF/RKS->UHF/UKS - triplet - external

Root	Eigenvalue (au)
0	0.123783
1	0.131890
2	0.163930

Stability Analysis indicates a stable HF/KS wave function.

One imaginary frequency (-441.56 cm^{-1}).

Optimized energies, coordinates, and frequencies for ωB97x-D3BJ/def2-TZVP calculations:

1 (singlet)

INNER ENERGY

The inner energy is: U= E(el) + E(ZPE) + E(vib) + E(rot) + E(trans)
E(el) - is the total energy from the electronic structure calculation
= E(kin-el) + E(nuc-el) + E(el-el) + E(nuc-nuc)
E(ZPE) - the the zero temperature vibrational energy from the frequency calculation
E(vib) - the the finite temperature correction to E(ZPE) due to population
of excited vibrational states
E(rot) - is the rotational thermal energy
E(trans)- is the translational thermal energy

Summary of contributions to the inner energy U:

Electronic energy ...	-427.88477573 Eh		
Zero point energy ...	0.22592209 Eh	141.77 kcal/mol	
Thermal vibrational correction ...	0.00531156 Eh	3.33 kcal/mol	
Thermal rotational correction ...	0.00141627 Eh	0.89 kcal/mol	
Thermal translational correction ...	0.00141627 Eh	0.89 kcal/mol	

Total thermal energy -427.65070953 Eh

Summary of corrections to the electronic energy:
(perhaps to be used in another calculation)
Total thermal correction 0.00814411 Eh 5.11 kcal/mol
Non-thermal (ZPE) correction 0.22592209 Eh 141.77 kcal/mol

Total correction 0.23406620 Eh 146.88 kcal/mol

CARTESIAN COORDINATES (ANGSTROEM)

C	-1.969143	1.669767	0.184617
C	-1.435161	2.489296	-0.983601
C	-0.391189	1.633435	-1.714175
C	0.725634	1.232359	-0.742501
H	0.025743	2.210544	-2.546614
H	-0.869161	0.743980	-2.134624
C	-0.894456	1.243500	1.176548
C	0.138753	0.407114	0.409831
H	-0.332470	-0.502491	0.026651
H	0.935501	0.103060	1.097113
C	1.388964	2.496039	-0.180167
H	1.469700	0.631631	-1.274502
C	-0.238667	2.518921	1.723175
H	-1.335026	0.660067	1.987196
C	-0.770380	3.744494	-0.401474
H	-2.247754	2.762745	-1.659232
C	0.347888	3.338886	0.567196
H	-1.518162	4.355192	0.112709
H	-0.358037	4.344360	-1.219996
H	0.553987	2.241176	2.426414
H	-0.977731	3.108729	2.272992
H	0.822721	4.239537	0.968252
H	1.828851	3.081600	-0.994696
H	2.204098	2.221447	0.498070
C	-3.217606	1.368361	0.326594

SCF STABILITY ANALYSIS RESULT

RHF/RKS->UHF/UKS - triplet - external

Root	Eigenvalue (au)
0	0.044385

1	0.074039
2	0.131363

Stability Analysis indicates a stable HF/KS wave function.

No imaginary frequency.

1 (triplet)

INNER ENERGY

The inner energy is: U= E(el) + E(ZPE) + E(vib) + E(rot) + E(trans)
E(el) - is the total energy from the electronic structure calculation
= E(kin-el) + E(nuc-el) + E(el-el) + E(nuc-nuc)
E(ZPE) - the the zero temperature vibrational energy from the frequency calculation
E(vib) - the the finite temperature correction to E(ZPE) due to population
of excited vibrational states
E(rot) - is the rotational thermal energy
E(trans)- is the translational thermal energy

Summary of contributions to the inner energy U:
Electronic energy ... -427.80905084 Eh
Zero point energy ... 0.22453003 Eh 140.89 kcal/mol
Thermal vibrational correction ... 0.00534780 Eh 3.36 kcal/mol
Thermal rotational correction ... 0.00141627 Eh 0.89 kcal/mol
Thermal translational correction ... 0.00141627 Eh 0.89 kcal/mol
----
Total thermal energy -427.57634047 Eh

Summary of corrections to the electronic energy:
(perhaps to be used in another calculation)
Total thermal correction 0.00818034 Eh 5.13 kcal/mol
Non-thermal (ZPE) correction 0.22453003 Eh 140.89 kcal/mol

Total correction 0.23271038 Eh 146.03 kcal/mol

CARTESIAN COORDINATES (ANGSTROEM)

C	-1.967669	1.670832	0.184882
C	-1.438025	2.473993	-0.965727
C	-0.379418	1.631179	-1.710154
C	0.739983	1.236574	-0.743560
H	0.019315	2.222341	-2.540990

H	-0.847038	0.739372	-2.138025
C	-0.906654	1.249685	1.157259
C	0.147549	0.411587	0.402083
H	-0.311911	-0.503348	0.016384
H	0.930006	0.112476	1.106941
C	1.401869	2.499787	-0.181301
H	1.483946	0.635518	-1.275686
C	-0.232824	2.522847	1.716052
H	-1.358215	0.667633	1.962446
C	-0.761845	3.741869	-0.397045
H	-2.258269	2.741315	-1.634076
C	0.359472	3.340079	0.564992
H	-1.503611	4.360354	0.117152
H	-0.363427	4.331411	-1.229156
H	0.550131	2.225591	2.421423
H	-0.964522	3.117990	2.270509
H	0.831562	4.241795	0.967427
H	1.841062	3.085896	-0.995557
H	2.216679	2.225951	0.497321
C	-3.341250	1.361021	0.352174

SCF STABILITY ANALYSIS RESULT

UHF/UKS->UHF/UKS - singlet - internal

Root	Eigenvalue (au)
0	0.012402
1	0.082150
2	0.131981

Stability Analysis indicates a stable HF/KS wave function.

No imaginary frequency.

10

INNER ENERGY

The inner energy is: U= E(el) + E(ZPE) + E(vib) + E(rot) + E(trans)
E(el) - is the total energy from the electronic structure calculation
= E(kin-el) + E(nuc-el) + E(el-el) + E(nuc-nuc)
E(ZPE) - the the zero temperature vibrational energy from the frequency calculation
E(vib) - the the finite temperature correction to E(ZPE) due to population

of excited vibrational states
E(rot) - is the rotational thermal energy
E(trans)- is the translational thermal energy

Summary of contributions to the inner energy U:

Electronic energy	...	-427.88837237 Eh	
Zero point energy	...	0.22727582 Eh	142.62 kcal/mol
Thermal vibrational correction	...	0.00488357 Eh	3.06 kcal/mol
Thermal rotational correction	...	0.00141627 Eh	0.89 kcal/mol
Thermal translational correction	...	0.00141627 Eh	0.89 kcal/mol

----

Total thermal energy -427.65338044 Eh

Summary of corrections to the electronic energy:
(perhaps to be used in another calculation)

Total thermal correction	0.00771611 Eh	4.84 kcal/mol
Non-thermal (ZPE) correction	0.22727582 Eh	142.62 kcal/mol

Total correction 0.23499193 Eh 147.46 kcal/mol

CARTESIAN COORDINATES (ANGSTROEM)

C	-1.240877	3.120245	-0.529414
C	-0.797905	4.261456	-1.363551
C	0.552792	3.806807	-1.960532
C	-0.763083	2.532157	0.405171
C	0.497918	2.667006	1.170959
C	1.613279	2.499106	0.114701
C	1.695888	3.644618	-0.926123
H	0.884448	4.563813	-2.681470
H	0.404087	2.876870	-2.515547
H	1.490689	1.534780	-0.385171
H	2.580090	2.472937	0.631840
C	1.861711	4.965598	-0.176764
H	2.601248	3.449911	-1.511440
C	0.471401	4.113478	1.713624
H	0.631476	1.953972	1.987135
C	-0.588835	5.418876	-0.360846
H	-1.487079	4.559476	-2.156210
C	0.587059	5.213376	0.628110
H	-1.520272	5.593236	0.184069
H	-0.370364	6.332365	-0.927091
H	1.325019	4.245413	2.389617
H	-0.434715	4.256091	2.308162

H	0.686977	6.159187	1.171861
H	2.033616	5.786094	-0.881690
H	2.729930	4.919379	0.489442

SCF STABILITY ANALYSIS RESULT

RHF/RKS->UHF/UKS - triplet - external

Root	Eigenvalue (au)
0	0.048249
1	0.095898
2	0.159668

Stability Analysis indicates a stable HF/KS wave function.

No imaginary frequency.

TS (1 to 10)

INNER ENERGY

The inner energy is: U= E(el) + E(ZPE) + E(vib) + E(rot) + E(trans)
E(el) - is the total energy from the electronic structure calculation
= E(kin-el) + E(nuc-el) + E(el-el) + E(nuc-nuc)
E(ZPE) - the the zero temperature vibrational energy from the frequency calculation
E(vib) - the the finite temperature correction to E(ZPE) due to population
of excited vibrational states
E(rot) - is the rotational thermal energy
E(trans)- is the translational thermal energy

Summary of contributions to the inner energy U:
Electronic energy ... -427.86005685 Eh
Zero point energy ... 0.22504027 Eh 141.21 kcal/mol
Thermal vibrational correction ... 0.00478491 Eh 3.00 kcal/mol
Thermal rotational correction ... 0.00141627 Eh 0.89 kcal/mol
Thermal translational correction ... 0.00141627 Eh 0.89 kcal/mol
----
Total thermal energy -427.62739913 Eh

Summary of corrections to the electronic energy:
(perhaps to be used in another calculation)
Total thermal correction 0.00761745 Eh 4.78 kcal/mol

Non-thermal (ZPE) correction 0.22504027 Eh 141.21 kcal/mol

Total correction 0.23265772 Eh 145.99 kcal/mol

CARTESIAN COORDINATES (ANGSTROEM)

C	-1.744894	-0.603916	0.470520
C	-1.251289	0.461123	-1.101535
C	-0.221628	-0.503061	-1.692594
C	0.909750	-0.878519	-0.722442
H	0.230671	-0.008499	-2.560858
H	-0.733657	-1.395398	-2.060204
C	-0.584032	-0.916912	1.308140
C	0.418864	-1.718841	0.463327
H	-0.050936	-2.641309	0.112706
H	1.272583	-1.994286	1.090847
C	1.567066	0.403275	-0.197947
H	1.648853	-1.466014	-1.276084
C	0.040962	0.404524	1.783948
H	-0.932567	-1.508543	2.159336
C	-0.604392	1.633960	-0.363230
H	-1.859824	0.854274	-1.909579
C	0.534109	1.222956	0.584012
H	-1.375932	2.192782	0.171812
H	-0.184649	2.306435	-1.121339
H	0.886678	0.180976	2.442300
H	-0.692900	0.972429	2.361451
H	1.005094	2.135716	0.962015
H	1.957294	0.993153	-1.034178
H	2.416616	0.154530	0.447127
C	-2.651840	-0.280835	-0.317552

SCF STABILITY ANALYSIS RESULT

RHF/RKS->UHF/UKS - triplet - external

Root	Eigenvalue (au)
0	0.142181
1	0.148907
2	0.167707

Stability Analysis indicates a stable HF/KS wave function.

One imaginary frequency (-439.96 cm^{-1}).

Single point energies and T1 diagnostics for DLPNO-CCSD(T)/def2-TZVP//B2PLYP/def2-TZVP calculations:

1 (singlet)

------------------------- --------------------
FINAL SINGLE POINT ENERGY -426.659537371009
------------------------- --------------------
T1 diagnostic ... 0.010964611

1 (triplet)

------------------------- --------------------
FINAL SINGLE POINT ENERGY -426.580801100666
------------------------- --------------------
T1 diagnostic ... 0.018497789

10

------------------------- --------------------
FINAL SINGLE POINT ENERGY -426.670261734749
------------------------- --------------------
T1 diagnostic ... 0.009906300

TS (1 to 10)

------------------------- --------------------
FINAL SINGLE POINT ENERGY -426.636642063238
------------------------- --------------------
T1 diagnostic ... 0.010902760

Single point energies and T1 diagnostics for DLPNO-CCSD(T)/def2-TZVP//B3LYP/def2-TZVP calculations:

1 (singlet)

------------------------- --------------------
FINAL SINGLE POINT ENERGY -426.659476980130
------------------------- --------------------
T1 diagnostic ... 0.010958141

10

------------------------- --------------------
FINAL SINGLE POINT ENERGY -426.669961462427
------------------------- --------------------
T1 diagnostic ... 0.009851681

TS (1 to 10)

------------------------- --------------------

FINAL SINGLE POINT ENERGY -426.636482727749
------------------------- --------------------
T1 diagnostic ... 0.010757788

Single point energies and T1 diagnostics for DLPNO-CCSD(T)/def2-TZVP//PBE0/def2-TZVP calculations:

1 (singlet)
------------------------- --------------------
FINAL SINGLE POINT ENERGY -426.659104267427
------------------------- --------------------
T1 diagnostic ... 0.010949806

10
------------------------- --------------------
FINAL SINGLE POINT ENERGY -426.669474741547
------------------------- --------------------
T1 diagnostic ... 0.009838084

TS (1 to 10)
------------------------- --------------------
FINAL SINGLE POINT ENERGY -426.636048989728
------------------------- --------------------
T1 diagnostic ... 0.011034788

Single point energies and T1 diagnostics for DLPNO-CCSD(T)/def2-TZVP//ωB97x-D3BJ/def2-TZVP calculations:

1 (singlet)
------------------------- --------------------
FINAL SINGLE POINT ENERGY -426.659546772780
------------------------- --------------------
T1 diagnostic ... 0.010955629

1 (triplet)
------------------------- --------------------
FINAL SINGLE POINT ENERGY -426.580014454794
------------------------- --------------------
T1 diagnostic ... 0.012722613

10
------------------------- --------------------
FINAL SINGLE POINT ENERGY -426.669772231807
------------------------- --------------------
T1 diagnostic ... 0.009829049

TS (1 to 10)

------------------------ --------------------
FINAL SINGLE POINT ENERGY -426.636460872114
------------------------ --------------------

T1 diagnostic ... 0.011180485

Cyclohexylidene System

Optimized energies, coordinates, stability analyses, and frequencies for B2PLYP/def2-TZVP calculations:

20

INNER ENERGY

The inner energy is: U= E(el) + E(ZPE) + E(vib) + E(rot) + E(trans)
E(el) - is the total energy from the electronic structure calculation
= E(kin-el) + E(nuc-el) + E(el-el) + E(nuc-nuc)
E(ZPE) - the the zero temperature vibrational energy from the frequency calculation
E(vib) - the the finite temperature correction to E(ZPE) due to population
of excited vibrational states
E(rot) - is the rotational thermal energy
E(trans)- is the translational thermal energy

Summary of contributions to the inner energy U:
Electronic energy ... -272.47761252 Eh
Zero point energy ... 0.15064564 Eh 94.53 kcal/mol
Thermal vibrational correction ... 0.00404478 Eh 2.54 kcal/mol
Thermal rotational correction ... 0.00141627 Eh 0.89 kcal/mol
Thermal translational correction ... 0.00141627 Eh 0.89 kcal/mol
----
Total thermal energy -272.32008956 Eh

Summary of corrections to the electronic energy:
(perhaps to be used in another calculation)
Total thermal correction 0.00687732 Eh 4.32 kcal/mol
Non-thermal (ZPE) correction 0.15064564 Eh 94.53 kcal/mol

Total correction 0.15752296 Eh 98.85 kcal/mol

CARTESIAN COORDINATES (ANGSTROEM)

C -3.774026 3.012270 -0.240633
C -4.579147 1.806778 0.243967

C	-3.934404	0.488432	-0.190842
H	-5.600047	1.852790	-0.141230
H	-4.645277	1.825696	1.334875
C	-2.476352	0.475725	0.229850
H	-3.968189	0.397584	-1.280928
H	-4.465419	-0.367279	0.224716
C	-1.633299	1.666349	-0.187272
C	-2.326504	2.959877	0.247598
H	-1.537494	1.642373	-1.277185
H	-0.629570	1.595951	0.230423
H	-1.765856	3.815378	-0.135102
H	-2.305827	3.023291	1.338660
H	-4.247530	3.935911	0.097304
H	-3.784837	3.036968	-1.335542
C	-1.996771	-0.467293	0.979879

SCF STABILITY ANALYSIS RESULT

RHF/RKS->UHF/UKS - triplet - external

Root	Eigenvalue (au)
0	0.015448
1	0.061643
2	0.091280

Stability Analysis indicates a stable HF/KS wave function.

No imaginary frequency.

21

INNER ENERGY

The inner energy is: U= E(el) + E(ZPE) + E(vib) + E(rot) + E(trans)
E(el) - is the total energy from the electronic structure calculation
= E(kin-el) + E(nuc-el) + E(el-el) + E(nuc-nuc)
E(ZPE) - the the zero temperature vibrational energy from the frequency calculation
E(vib) - the the finite temperature correction to E(ZPE) due to population
of excited vibrational states
E(rot) - is the rotational thermal energy
E(trans)- is the translational thermal energy

Summary of contributions to the inner energy U:

```
Electronic energy                ...   -272.51863748 Eh
Zero point energy                ...      0.15151949 Eh      95.08 kcal/mol
Thermal vibrational correction   ...      0.00377513 Eh       2.37 kcal/mol
Thermal rotational correction    ...      0.00141627 Eh       0.89 kcal/mol
Thermal translational correction ...      0.00141627 Eh       0.89 kcal/mol
-----------------------------------------------------------------------
Total thermal energy                   -272.36051032 Eh


Summary of corrections to the electronic energy:
(perhaps to be used in another calculation)
Total thermal correction                  0.00660767 Eh       4.15 kcal/mol
Non-thermal (ZPE) correction              0.15151949 Eh      95.08 kcal/mol
-----------------------------------------------------------------------
Total correction                          0.15812716 Eh      99.23 kcal/mol

---------------------------------
CARTESIAN COORDINATES (ANGSTROEM)
---------------------------------
  C     -3.635403    2.748164   -0.338094
  C     -4.599318    1.718806    0.305408
  C     -4.386312    0.241610   -0.112939
  H     -5.627921    1.999653    0.063839
  H     -4.501378    1.786411    1.391804
  C     -1.856427    0.621468   -0.104925
  H     -4.787347    0.064848   -1.114157
  H     -4.911912   -0.431319    0.565613
  C     -1.159420    1.908250   -0.094899
  C     -2.243968    2.935294    0.318492
  H     -0.776217    2.136375   -1.092797
  H     -0.311050    1.945466    0.589475
  H     -1.875207    3.937489    0.084248
  H     -2.365351    2.890294    1.403708
  H     -4.136403    3.717479   -0.290177
  H     -3.513293    2.523422   -1.402406
  C     -2.933614    0.066420   -0.112474

-----------------------------
SCF STABILITY ANALYSIS RESULT
-----------------------------

RHF/RKS->UHF/UKS - triplet - external

   Root    Eigenvalue (au)
     0      0.034347
     1      0.097712
```

2 0.127528

Stability Analysis indicates a stable HF/KS wave function.

No imaginary frequency.

TS (20 to 21)

INNER ENERGY

The inner energy is: U= E(el) + E(ZPE) + E(vib) + E(rot) + E(trans)
E(el) - is the total energy from the electronic structure calculation
= E(kin-el) + E(nuc-el) + E(el-el) + E(nuc-nuc)
E(ZPE) - the the zero temperature vibrational energy from the frequency calculation
E(vib) - the the finite temperature correction to E(ZPE) due to population
of excited vibrational states
E(rot) - is the rotational thermal energy
E(trans)- is the translational thermal energy

Summary of contributions to the inner energy U:
Electronic energy ... -272.45738179 Eh
Zero point energy ... 0.14929846 Eh 93.69 kcal/mol
Thermal vibrational correction ... 0.00365971 Eh 2.30 kcal/mol
Thermal rotational correction ... 0.00141627 Eh 0.89 kcal/mol
Thermal translational correction ... 0.00141627 Eh 0.89 kcal/mol
----
Total thermal energy -272.30159107 Eh

Summary of corrections to the electronic energy:
(perhaps to be used in another calculation)
Total thermal correction 0.00649225 Eh 4.07 kcal/mol
Non-thermal (ZPE) correction 0.14929846 Eh 93.69 kcal/mol

Total correction 0.15579071 Eh 97.76 kcal/mol

CARTESIAN COORDINATES (ANGSTROEM)

C	-0.568594	1.145873	-0.235265
C	-1.369887	-0.052507	0.281342
C	-0.888688	-1.416043	-0.194624
H	-2.406645	0.055718	-0.051415
H	-1.388967	-0.049737	1.373773
C	0.969255	-1.265657	0.284335

H	-0.737560	-1.458257	-1.272544
H	-1.615747	-2.175067	0.067339
C	1.659660	-0.059783	-0.161583
C	0.876719	1.190932	0.255703
H	1.788518	-0.083080	-1.248289
H	2.658531	-0.064364	0.279634
H	1.376912	2.074504	-0.144547
H	0.892844	1.271041	1.344855
H	-1.075458	2.065131	0.064756
H	-0.576587	1.132626	-1.330707
C	0.405694	-2.311329	0.687239

SCF STABILITY ANALYSIS RESULT

RHF/RKS->UHF/UKS - triplet - external

Root	Eigenvalue (au)
0	0.105234
1	0.136218
2	0.159785

Stability Analysis indicates a stable HF/KS wave function.

One imaginary frequency (-470.96 cm^{-1}).

Single point energies and T1 diagnostics for DLPNO-CCSD(T)/def2-TZVP//B2PLYP/def2-TZVP calculations:

20

------------------------- --------------------
FINAL SINGLE POINT ENERGY -272.099760603489
------------------------- --------------------
T1 diagnostic ... 0.011632781

21

------------------------- --------------------
FINAL SINGLE POINT ENERGY -272.137546412212
------------------------- --------------------
T1 diagnostic ... 0.010105545

TS (20 to 21)

------------------------- --------------------
FINAL SINGLE POINT ENERGY -272.077233552890
------------------------- --------------------

T1 diagnostic ... 0.011472400

4-Methylcyclohexylidene System

Optimized energies, coordinates, stability analyses, and frequencies for B2PLYP/def2-TZVP calculations:

22

INNER ENERGY

The inner energy is: U= E(el) + E(ZPE) + E(vib) + E(rot) + E(trans)
E(el) - is the total energy from the electronic structure calculation
= E(kin-el) + E(nuc-el) + E(el-el) + E(nuc-nuc)
E(ZPE) - the the zero temperature vibrational energy from the frequency calculation
E(vib) - the the finite temperature correction to E(ZPE) due to population
of excited vibrational states
E(rot) - is the rotational thermal energy
E(trans)- is the translational thermal energy

Summary of contributions to the inner energy U:
Electronic energy ... -311.76917348 Eh
Zero point energy ... 0.17837853 Eh 111.93 kcal/mol
Thermal vibrational correction ... 0.00553817 Eh 3.48 kcal/mol
Thermal rotational correction ... 0.00141627 Eh 0.89 kcal/mol
Thermal translational correction ... 0.00141627 Eh 0.89 kcal/mol
----
Total thermal energy -311.58242423 Eh

Summary of corrections to the electronic energy:
(perhaps to be used in another calculation)
Total thermal correction 0.00837071 Eh 5.25 kcal/mol
Non-thermal (ZPE) correction 0.17837853 Eh 111.93 kcal/mol

Total correction 0.18674925 Eh 117.19 kcal/mol

CARTESIAN COORDINATES (ANGSTROEM)

C	-2.282157	2.798562	-0.105425
C	-2.833270	1.401923	0.188028
C	-1.945371	0.293249	-0.381782
H	-3.841945	1.318889	-0.224897
H	-2.918397	1.271339	1.271079

C	-0.516533	0.421497	0.151167
C	0.094012	1.792547	-0.144716
H	0.114524	-0.356121	-0.286792
H	-0.521845	0.258290	1.233157
C	-0.838961	2.879153	0.353926
H	0.213764	1.923900	-1.224443
H	1.080627	1.890052	0.307043
H	-2.886046	3.568712	0.373288
H	-2.301335	2.990252	-1.182633
C	-2.523712	-1.084556	-0.080010
H	-1.908675	0.420734	-1.471303
H	-3.532169	-1.187082	-0.483709
H	-2.576721	-1.251534	0.998064
H	-1.907153	-1.875111	-0.510400
C	-0.443146	3.782822	1.195376

SCF STABILITY ANALYSIS RESULT

RHF/RKS->UHF/UKS - triplet - external

Root	Eigenvalue (au)
0	0.015489
1	0.061841
2	0.091340

Stability Analysis indicates a stable HF/KS wave function.

No imaginary frequency.

23

INNER ENERGY

The inner energy is: U= E(el) + E(ZPE) + E(vib) + E(rot) + E(trans)
E(el) - is the total energy from the electronic structure calculation
= E(kin-el) + E(nuc-el) + E(el-el) + E(nuc-nuc)
E(ZPE) - the the zero temperature vibrational energy from the frequency calculation
E(vib) - the the finite temperature correction to E(ZPE) due to population
of excited vibrational states
E(rot) - is the rotational thermal energy
E(trans)- is the translational thermal energy

Summary of contributions to the inner energy U:

Electronic energy ... -311.80823494 Eh
Zero point energy ... 0.17971985 Eh 112.78 kcal/mol
Thermal vibrational correction ... 0.00511833 Eh 3.21 kcal/mol
Thermal rotational correction ... 0.00141627 Eh 0.89 kcal/mol
Thermal translational correction ... 0.00141627 Eh 0.89 kcal/mol
----
Total thermal energy -311.62056422 Eh

Summary of corrections to the electronic energy:
(perhaps to be used in another calculation)
Total thermal correction 0.00795088 Eh 4.99 kcal/mol
Non-thermal (ZPE) correction 0.17971985 Eh 112.78 kcal/mol

Total correction 0.18767073 Eh 117.76 kcal/mol

CARTESIAN COORDINATES (ANGSTROEM)

C	-2.762599	2.984875	-0.159397
C	-2.843481	1.511450	0.314255
C	-1.848158	0.515343	-0.344546
H	-3.862194	1.155710	0.135848
H	-2.687444	1.485508	1.396216
C	-0.424596	0.484555	0.279148
C	0.567459	1.570830	-0.207603
H	0.022654	-0.493363	0.079561
H	-0.525029	0.567245	1.364855
C	-0.215320	2.805878	-0.231791
H	0.930203	1.334185	-1.211129
H	1.439774	1.612693	0.445888
H	-3.319631	3.635035	0.516379
H	-3.211198	3.091923	-1.150241
C	-2.444517	-0.891956	-0.251556
H	-1.753482	0.774289	-1.405331
H	-3.429295	-0.936561	-0.718443
H	-2.558184	-1.194703	0.792434
H	-1.804363	-1.626590	-0.741737
C	-1.331281	3.277805	-0.217337

SCF STABILITY ANALYSIS RESULT

RHF/RKS->UHF/UKS - triplet - external

Root	Eigenvalue (au)
0	0.033227
1	0.096891
2	0.127491

Stability Analysis indicates a stable HF/KS wave function.

No imaginary frequency.

TS (22 to 23)

```
------------
INNER ENERGY
------------

The inner energy is: U= E(el) + E(ZPE) + E(vib) + E(rot) + E(trans)
    E(el)   - is the total energy from the electronic structure calculation
              = E(kin-el) + E(nuc-el) + E(el-el) + E(nuc-nuc)
    E(ZPE)  - the the zero temperature vibrational energy from the frequency calculation
    E(vib)  - the the finite temperature correction to E(ZPE) due to population
              of excited vibrational states
    E(rot)  - is the rotational thermal energy
    E(trans)- is the translational thermal energy

Summary of contributions to the inner energy U:
Electronic energy                ...   -311.74890336 Eh
Zero point energy                ...      0.17727410 Eh     111.24 kcal/mol
Thermal vibrational correction   ...      0.00505418 Eh       3.17 kcal/mol
Thermal rotational correction    ...      0.00141627 Eh       0.89 kcal/mol
Thermal translational correction ...      0.00141627 Eh       0.89 kcal/mol
-----------------------------------------------------------------------
Total thermal energy                   -311.56374254 Eh


Summary of corrections to the electronic energy:
(perhaps to be used in another calculation)
Total thermal correction                  0.00788673 Eh       4.95 kcal/mol
Non-thermal (ZPE) correction              0.17727410 Eh     111.24 kcal/mol
-----------------------------------------------------------------------
Total correction                          0.18516083 Eh     116.19 kcal/mol

---------------------------------
CARTESIAN COORDINATES (ANGSTROEM)
---------------------------------
  C     -0.794609    1.796679   -0.156783
  C     -1.180188    0.384058    0.248099
  C     -0.292512   -0.735271   -0.312195
```

H	-2.203316	0.211540	-0.102229
H	-1.208150	0.323185	1.339452
C	1.154796	-0.654037	0.177914
C	1.841768	0.666817	-0.192091
H	1.727293	-1.480656	-0.248860
H	1.174506	-0.770805	1.264659
C	1.072755	1.796600	0.314847
H	1.961650	0.745195	-1.277023
H	2.841791	0.721631	0.243176
H	-1.585125	2.495916	0.087250
H	-0.600149	1.888020	-1.225416
C	-0.887736	-2.099182	0.025986
H	-0.280841	-0.629309	-1.404793
H	-1.902993	-2.193471	-0.361749
H	-0.929103	-2.242841	1.107914
H	-0.289559	-2.908075	-0.395882
C	0.379721	2.684006	0.867723

SCF STABILITY ANALYSIS RESULT

RHF/RKS->UHF/UKS - triplet - external

Root	Eigenvalue (au)
0	0.103979
1	0.133956
2	0.159704

Stability Analysis indicates a stable HF/KS wave function.

One imaginary frequency (-464.62 cm^{-1}).

Single point energies and T1 diagnostics for DLPNO-CCSD(T)/def2-TZVP//B2PLYP/def2-TZVP calculations:

22

------------------------- --------------------
FINAL SINGLE POINT ENERGY -311.337264840971
------------------------- --------------------
T1 diagnostic ... 0.011250907

23

------------------------- --------------------
FINAL SINGLE POINT ENERGY -311.373272827783
------------------------- --------------------

T1 diagnostic ... 0.009924832

TS (22 to 23)

------------------------ --------------------
FINAL SINGLE POINT ENERGY -311.314715945215
------------------------ --------------------
T1 diagnostic ... 0.011257611

<u>ω-camphylidene System</u>

Optimized energies, coordinates, stability analyses, and frequencies for B2PLYP/def2-TZVP calculations:

12

INNER ENERGY

The inner energy is: U= E(el) + E(ZPE) + E(vib) + E(rot) + E(trans)
E(el) - is the total energy from the electronic structure calculation
= E(kin-el) + E(nuc-el) + E(el-el) + E(nuc-nuc)
E(ZPE) - the the zero temperature vibrational energy from the frequency calculation
E(vib) - the the finite temperature correction to E(ZPE) due to population
of excited vibrational states
E(rot) - is the rotational thermal energy
E(trans)- is the translational thermal energy

Summary of contributions to the inner energy U:
Electronic energy ... -389.13350173 Eh
Zero point energy ... 0.21344077 Eh 133.94 kcal/mol
Thermal vibrational correction ... 0.00658421 Eh 4.13 kcal/mol
Thermal rotational correction ... 0.00141627 Eh 0.89 kcal/mol
Thermal translational correction ... 0.00141627 Eh 0.89 kcal/mol
----
Total thermal energy -388.91064421 Eh

Summary of corrections to the electronic energy:
(perhaps to be used in another calculation)
Total thermal correction 0.00941676 Eh 5.91 kcal/mol
Non-thermal (ZPE) correction 0.21344077 Eh 133.94 kcal/mol

Total correction 0.22285752 Eh 139.84 kcal/mol

CARTESIAN COORDINATES (ANGSTROEM)

C	-2.162302	1.849048	-0.109411
C	-2.375077	0.310620	-0.194363
C	-1.460621	-0.230522	0.922807
H	-2.109322	-0.106519	-1.163570
H	-3.411875	0.041279	0.012488
C	-0.002229	-0.052690	0.431325
C	0.294194	1.457422	0.450959
C	-1.071656	1.996562	0.967104
H	-3.072429	2.346243	0.227311
H	-1.889505	2.294163	-1.062904
C	-1.505960	0.905975	1.959382
H	-1.700976	-1.231259	1.268919
H	-0.825190	0.754387	2.793777
H	-2.515184	1.061633	2.345386
H	-0.984332	3.006042	1.368983
C	0.650734	-1.134572	0.167097
C	1.416902	1.771200	1.447121
H	1.182037	1.428006	2.452562
H	1.586318	2.849629	1.481890
H	2.343131	1.286815	1.139127
C	0.699895	1.975826	-0.929424
H	0.021835	1.641276	-1.711109
H	1.696823	1.616723	-1.184672
H	0.721173	3.068011	-0.930387

SCF STABILITY ANALYSIS RESULT

RHF/RKS->UHF/UKS - triplet - external

Root	Eigenvalue (au)
0	0.019342
1	0.061684
2	0.090519

Stability Analysis indicates a stable HF/KS wave function.

No imaginary frequency.

13

INNER ENERGY

The inner energy is: U= E(el) + E(ZPE) + E(vib) + E(rot) + E(trans)

E(el) - is the total energy from the electronic structure calculation
= E(kin-el) + E(nuc-el) + E(el-el) + E(nuc-nuc)
E(ZPE) - the the zero temperature vibrational energy from the frequency calculation
E(vib) - the the finite temperature correction to E(ZPE) due to population
of excited vibrational states
E(rot) - is the rotational thermal energy
E(trans)- is the translational thermal energy

Summary of contributions to the inner energy U:
Electronic energy ... -389.15527622 Eh
Zero point energy ... 0.21442049 Eh 134.55 kcal/mol
Thermal vibrational correction ... 0.00626716 Eh 3.93 kcal/mol
Thermal rotational correction ... 0.00141627 Eh 0.89 kcal/mol
Thermal translational correction ... 0.00141627 Eh 0.89 kcal/mol
----
Total thermal energy -388.93175603 Eh

Summary of corrections to the electronic energy:
(perhaps to be used in another calculation)
Total thermal correction 0.00909970 Eh 5.71 kcal/mol
Non-thermal (ZPE) correction 0.21442049 Eh 134.55 kcal/mol

Total correction 0.22352019 Eh 140.26 kcal/mol

CARTESIAN COORDINATES (ANGSTROEM)

C	-2.014310	1.552121	-0.265892
C	-2.622353	0.142795	-0.006828
C	-1.744255	-0.455713	1.108320
H	-2.626011	-0.477414	-0.901212
H	-3.651479	0.222729	0.349192
C	-0.405519	-0.762237	0.525499
C	0.538507	1.529930	0.378581
C	-0.953215	1.809653	0.843087
H	-2.792137	2.313777	-0.210399
H	-1.586693	1.617214	-1.262113
C	-1.423642	0.822881	1.933487
H	-2.225607	-1.249688	1.674879
H	-0.700977	0.648499	2.727740
H	-2.347867	1.196265	2.384505
H	-0.991050	2.843870	1.195643
C	0.434835	0.056218	0.172291
C	1.530184	1.880291	1.492293
H	1.305178	1.352429	2.416438

H	1.503794	2.954236	1.689128
H	2.544226	1.614930	1.192980
C	0.913523	2.321500	-0.873517
H	0.317323	2.039099	-1.737567
H	1.960181	2.145127	-1.123085
H	0.782933	3.391194	-0.695778

SCF STABILITY ANALYSIS RESULT

RHF/RKS->UHF/UKS - triplet - external

Root	Eigenvalue (au)
0	0.027690
1	0.084461
2	0.150710

Stability Analysis indicates a stable HF/KS wave function.

No imaginary frequency.

TS (12 to 13)

INNER ENERGY

The inner energy is: U= E(el) + E(ZPE) + E(vib) + E(rot) + E(trans)
E(el) - is the total energy from the electronic structure calculation
= E(kin-el) + E(nuc-el) + E(el-el) + E(nuc-nuc)
E(ZPE) - the the zero temperature vibrational energy from the frequency calculation
E(vib) - the the finite temperature correction to E(ZPE) due to population
of excited vibrational states
E(rot) - is the rotational thermal energy
E(trans)- is the translational thermal energy

Summary of contributions to the inner energy U:

Electronic energy ...	-389.12122951 Eh		
Zero point energy ...	0.21283735 Eh	133.56 kcal/mol	
Thermal vibrational correction ...	0.00603384 Eh	3.79 kcal/mol	
Thermal rotational correction ...	0.00141627 Eh	0.89 kcal/mol	
Thermal translational correction ...	0.00141627 Eh	0.89 kcal/mol	

Total thermal energy -388.89952578 Eh

Summary of corrections to the electronic energy:
(perhaps to be used in another calculation)
Total thermal correction 0.00886638 Eh 5.56 kcal/mol
Non-thermal (ZPE) correction 0.21283735 Eh 133.56 kcal/mol

Total correction 0.22170373 Eh 139.12 kcal/mol

CARTESIAN COORDINATES (ANGSTROEM)

C	-1.506646	0.497402	-0.716896
C	-1.916756	-0.997987	-0.622828
C	-0.988046	-1.525347	0.488887
H	-1.769347	-1.537317	-1.556047
H	-2.960611	-1.108001	-0.326575
C	0.381355	-1.574577	-0.035554
C	1.013520	0.238875	-0.093695
C	-0.417196	0.672604	0.363364
H	-2.350423	1.142981	-0.472500
H	-1.170519	0.777408	-1.710920
C	-0.901369	-0.311198	1.434958
H	-1.293835	-2.466670	0.941269
H	-0.207368	-0.459529	2.258194
H	-1.879853	-0.038360	1.834100
H	-0.338202	1.707418	0.701913
C	1.565810	-1.558507	-0.460381
C	2.034171	0.591543	0.977925
H	1.832541	0.084137	1.918236
H	2.005010	1.671319	1.156792
H	3.033852	0.314012	0.654356
C	1.385776	0.849886	-1.434196
H	0.818427	0.414157	-2.252203
H	2.440859	0.688484	-1.638901
H	1.188852	1.927266	-1.409298

SCF STABILITY ANALYSIS RESULT

RHF/RKS->UHF/UKS - triplet - external

Root	Eigenvalue (au)
0	0.108012
1	0.150650
2	0.155554

Stability Analysis indicates a stable HF/KS wave function.

One imaginary frequency (-256.53 cm^{-1}).

Single point energies and T1 diagnostics for DLPNO-CCSD(T)/def2-TZVP//B2PLYP/def2-TZVP calculations:

12

```
-------------------------   --------------------
FINAL SINGLE POINT ENERGY      -388.596808933107
-------------------------   --------------------
T1 diagnostic                          ...    0.011251918
```

13

```
-------------------------   --------------------
FINAL SINGLE POINT ENERGY      -388.615862614955
-------------------------   --------------------
T1 diagnostic                          ...    0.011117878
```

TS (12 to 13)

```
-------------------------   --------------------
FINAL SINGLE POINT ENERGY      -388.583213396464
-------------------------   --------------------
T1 diagnostic                          ...    0.010017911
```

ω-longifolylidene System

Optimized energies, coordinates, stability analyses, and frequencies for B2PLYP/def2-TZVP calculations:

15

```
------------
INNER ENERGY
------------

The inner energy is: U= E(el) + E(ZPE) + E(vib) + E(rot) + E(trans)
   E(el)   - is the total energy from the electronic structure calculation
            = E(kin-el) + E(nuc-el) + E(el-el) + E(nuc-nuc)
   E(ZPE)  - the the zero temperature vibrational energy from the frequency calculation
   E(vib)  - the the finite temperature correction to E(ZPE) due to population
            of excited vibrational states
   E(rot)  - is the rotational thermal energy
   E(trans)- is the translational thermal energy

Summary of contributions to the inner energy U:
Electronic energy              ...   -584.36270678 Eh
Zero point energy              ...      0.33615971 Eh     210.94 kcal/mol
```

Thermal vibrational correction ... 0.01118399 Eh 7.02 kcal/mol
Thermal rotational correction ... 0.00141627 Eh 0.89 kcal/mol
Thermal translational correction ... 0.00141627 Eh 0.89 kcal/mol
----
Total thermal energy -584.01253054 Eh

Summary of corrections to the electronic energy:
(perhaps to be used in another calculation)
Total thermal correction 0.01401653 Eh 8.80 kcal/mol
Non-thermal (ZPE) correction 0.33615971 Eh 210.94 kcal/mol

Total correction 0.35017624 Eh 219.74 kcal/mol

CARTESIAN COORDINATES (ANGSTROEM)

C	-1.888772	1.680117	-0.316252
C	-2.298700	0.233605	0.082768
C	-1.609014	0.049354	1.447201
H	-1.973147	-0.518035	-0.633017
H	-3.380226	0.144763	0.198003
C	-0.094158	-0.038324	1.142776
C	0.383568	1.386083	0.803951
C	-0.978101	2.129235	0.841970
H	-2.761756	2.331817	-0.359846
H	-1.405818	1.731030	-1.288616
C	-1.706106	1.477306	2.038345
H	-1.998945	-0.768042	2.046101
H	-0.861942	3.210174	0.886574
C	0.463993	-1.201347	1.198725
C	1.377108	1.916319	1.888965
C	1.083841	1.424254	-0.556641
H	0.504315	0.934502	-1.335671
H	2.044990	0.914044	-0.493391
H	1.267872	2.459761	-0.853454
C	-1.234209	1.757089	3.495812
H	-2.759219	1.770427	1.985777
C	-0.367266	3.028486	3.562863
C	0.933252	3.119368	2.724262
H	-0.120058	3.185915	4.615796
H	-1.007457	3.870300	3.286042
H	1.764372	3.350762	3.393787
H	0.856404	3.982280	2.059347
H	2.289634	2.221988	1.372499
H	1.673140	1.095325	2.536108

C	-0.537799	0.574199	4.185522
H	0.365787	0.239244	3.690276
H	-1.207453	-0.284735	4.249610
H	-0.277678	0.862064	5.206205
C	-2.499167	2.042187	4.324518
H	-3.042767	2.902946	3.930371
H	-2.244867	2.248913	5.365576
H	-3.171161	1.181617	4.308276

SCF STABILITY ANALYSIS RESULT

RHF/RKS->UHF/UKS - triplet - external

Root	Eigenvalue (au)
0	0.018158
1	0.057721
2	0.087481

Stability Analysis indicates a stable HF/KS wave function.

No imaginary frequency.

16

INNER ENERGY

The inner energy is: U= E(el) + E(ZPE) + E(vib) + E(rot) + E(trans)
E(el) - is the total energy from the electronic structure calculation
= E(kin-el) + E(nuc-el) + E(el-el) + E(nuc-nuc)
E(ZPE) - the the zero temperature vibrational energy from the frequency calculation
E(vib) - the the finite temperature correction to E(ZPE) due to population
of excited vibrational states
E(rot) - is the rotational thermal energy
E(trans)- is the translational thermal energy

Summary of contributions to the inner energy U:
Electronic energy ... -584.38482098 Eh
Zero point energy ... 0.33712675 Eh 211.55 kcal/mol
Thermal vibrational correction ... 0.01083568 Eh 6.80 kcal/mol
Thermal rotational correction ... 0.00141627 Eh 0.89 kcal/mol
Thermal translational correction ... 0.00141627 Eh 0.89 kcal/mol

----

Total thermal energy -584.03402600 Eh

Summary of corrections to the electronic energy:
(perhaps to be used in another calculation)
Total thermal correction 0.01366823 Eh 8.58 kcal/mol
Non-thermal (ZPE) correction 0.33712675 Eh 211.55 kcal/mol

Total correction 0.35079498 Eh 220.13 kcal/mol

CARTESIAN COORDINATES (ANGSTROEM)

C	-1.733527	1.377688	-0.370539
C	-2.589944	0.208262	0.195795
C	-1.989287	-0.050904	1.588266
H	-2.549815	-0.675604	-0.438327
H	-3.636663	0.501210	0.299159
C	-0.633315	-0.630378	1.371786
C	0.636946	1.407960	0.761202
C	-0.859872	1.925367	0.798488
H	-2.377283	2.170759	-0.751384
H	-1.126708	1.039475	-1.204953
C	-1.670314	1.423274	2.017784
H	-2.652941	-0.582977	2.266792
H	-0.835606	3.016016	0.770745
C	0.348621	-0.044294	0.930528
C	1.491863	1.962648	1.941449
C	1.321841	1.784434	-0.552280
H	0.799785	1.400203	-1.425249
H	2.335335	1.383225	-0.567719
H	1.386857	2.871761	-0.638981
C	-1.189898	1.703774	3.464847
H	-2.630904	1.945711	1.923069
C	-0.363291	3.006677	3.529102
C	0.917620	3.179404	2.668972
H	-0.098218	3.150204	4.579990
H	-1.039667	3.831098	3.286534
H	1.708673	3.564023	3.316131
H	0.746175	3.967654	1.932535
H	2.464433	2.254889	1.539773
H	1.699742	1.168513	2.652176
C	-0.458845	0.538810	4.151492
H	0.438230	0.209505	3.644076
H	-1.114849	-0.329204	4.232631
H	-0.187796	0.841533	5.165425
C	-2.454469	1.946368	4.310522

H	-3.016747	2.806691	3.943019
H	-2.195624	2.131572	5.354557
H	-3.111564	1.074745	4.280179

SCF STABILITY ANALYSIS RESULT

RHF/RKS->UHF/UKS - triplet - external

Root	Eigenvalue (au)
0	0.029297
1	0.083836
2	0.148373

Stability Analysis indicates a stable HF/KS wave function.

No imaginary frequency.

TS (15 to 16)

INNER ENERGY

The inner energy is: U= E(el) + E(ZPE) + E(vib) + E(rot) + E(trans)
E(el) - is the total energy from the electronic structure calculation
= E(kin-el) + E(nuc-el) + E(el-el) + E(nuc-nuc)
E(ZPE) - the the zero temperature vibrational energy from the frequency calculation
E(vib) - the the finite temperature correction to E(ZPE) due to population
of excited vibrational states
E(rot) - is the rotational thermal energy
E(trans)- is the translational thermal energy

Summary of contributions to the inner energy U:
Electronic energy ... -584.35057515 Eh
Zero point energy ... 0.33563567 Eh 210.61 kcal/mol
Thermal vibrational correction ... 0.01061325 Eh 6.66 kcal/mol
Thermal rotational correction ... 0.00141627 Eh 0.89 kcal/mol
Thermal translational correction ... 0.00141627 Eh 0.89 kcal/mol

----
Total thermal energy -584.00149369 Eh

Summary of corrections to the electronic energy:
(perhaps to be used in another calculation)
Total thermal correction 0.01344580 Eh 8.44 kcal/mol

Non-thermal (ZPE) correction 0.33563567 Eh 210.61 kcal/mol

Total correction 0.34908147 Eh 219.05 kcal/mol

CARTESIAN COORDINATES (ANGSTROEM)

C	-1.124781	0.013463	-2.279774
C	-1.739309	-1.340467	-1.827493
C	-1.059885	-1.554972	-0.463066
H	-1.523882	-2.153920	-2.516983
H	-2.821046	-1.269951	-1.705448
C	0.358485	-1.826631	-0.727883
C	1.194456	-0.128590	-1.100083
C	-0.243373	0.467186	-1.091199
H	-1.905332	0.756992	-2.441595
H	-0.573336	-0.074313	-3.210634
C	-1.043190	-0.104702	0.092816
H	-1.522074	-2.309274	0.170650
H	-0.142813	1.552416	-1.075990
C	1.573226	-1.999984	-1.006714
C	2.057197	0.391784	0.073317
C	1.871771	0.129980	-2.439343
H	1.407697	-0.425302	-3.249463
H	2.916697	-0.162464	-2.394122
H	1.809667	1.200044	-2.669623
C	-0.601602	0.162919	1.558430
H	-2.062496	0.283293	0.001628
C	0.228688	1.461024	1.653476
C	1.530261	1.615701	0.823907
H	0.466164	1.601038	2.711124
H	-0.437330	2.288726	1.394394
H	2.329756	1.936467	1.494632
H	1.406844	2.438549	0.116262
H	3.035638	0.646870	-0.337829
H	2.247215	-0.414037	0.771768
C	0.100450	-1.019623	2.246172
H	-0.591681	-1.855665	2.361954
H	0.412499	-0.716704	3.247653
H	0.968998	-1.395100	1.719270
C	-1.886982	0.407475	2.369953
H	-2.434521	1.272796	1.991997
H	-1.654767	0.585703	3.421386
H	-2.547309	-0.460726	2.316452

SCF STABILITY ANALYSIS RESULT

RHF/RKS->UHF/UKS - triplet - external

Root	Eigenvalue (au)
0	0.105131
1	0.150487
2	0.153027

Stability Analysis indicates a stable HF/KS wave function.

One imaginary frequency (-254.31 cm^{-1}).

Single point energies and T1 diagnostics for DLPNO-CCSD(T)/def2-TZVP//B2PLYP/def2-TZVP calculations:

15

------------------------- --------------------
FINAL SINGLE POINT ENERGY -583.558244781931
------------------------- --------------------
T1 diagnostic ... 0.010473444

16

------------------------- --------------------
FINAL SINGLE POINT ENERGY -583.577913938002
------------------------- --------------------
T1 diagnostic ... 0.010481006

TS (15 to 16)

------------------------- --------------------
FINAL SINGLE POINT ENERGY -583.545036143992
------------------------- --------------------
T1 diagnostic ... 0.009585924

Potential Energy Surface Diagram for DLPNO-CCSD(T)/def2-TZVP//B2PLYP/def2-TZVP calculations:

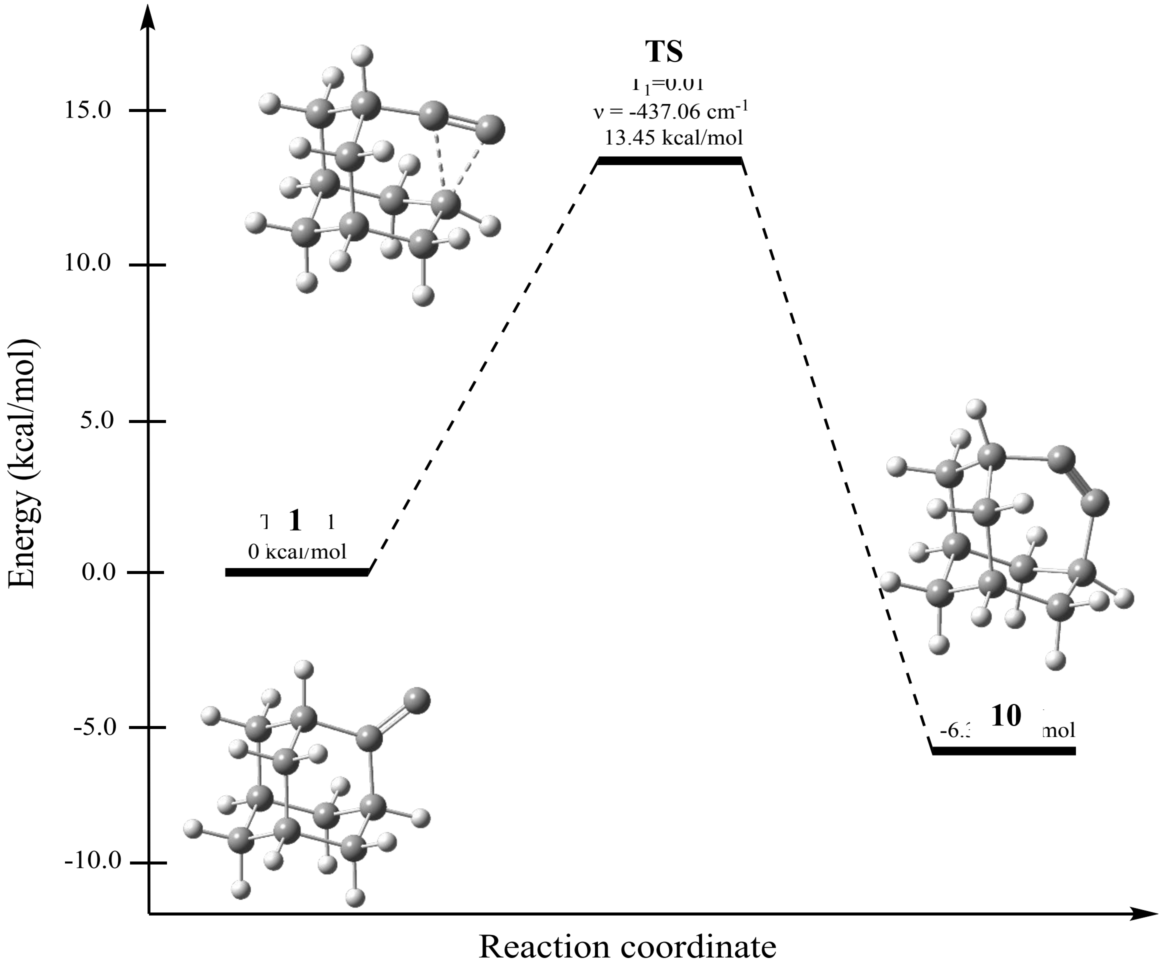

B2PLYP

Potential Energy Surface Diagram for DLPNO-CCSD(T)/def2-TZVP//B3LYP/def2-TZVP calculations:

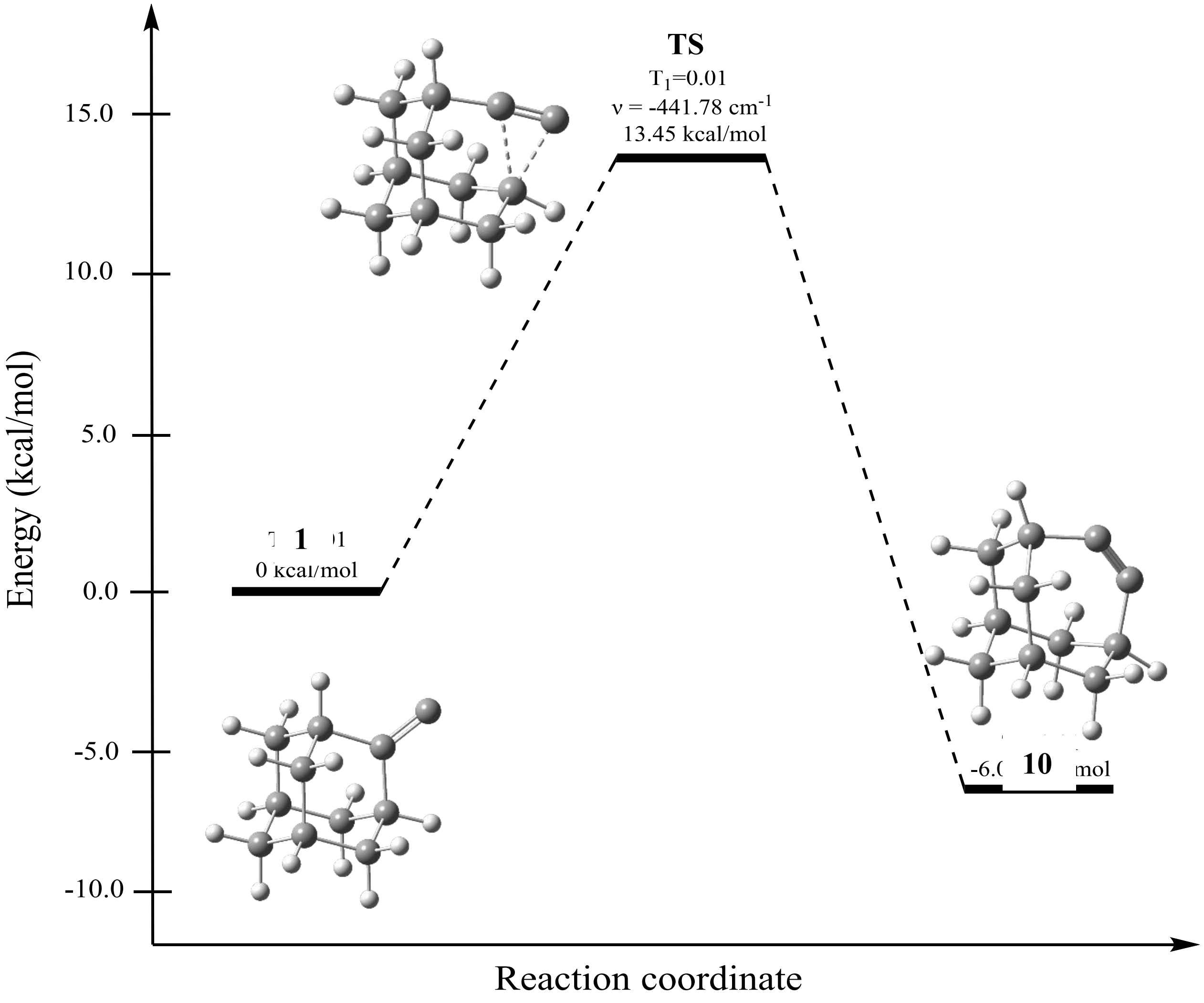

wb97x

Potential Energy Surface Diagram for DLPNO-CCSD(T)/def2-TZVP//PBE0/def2-TZVP calculations:

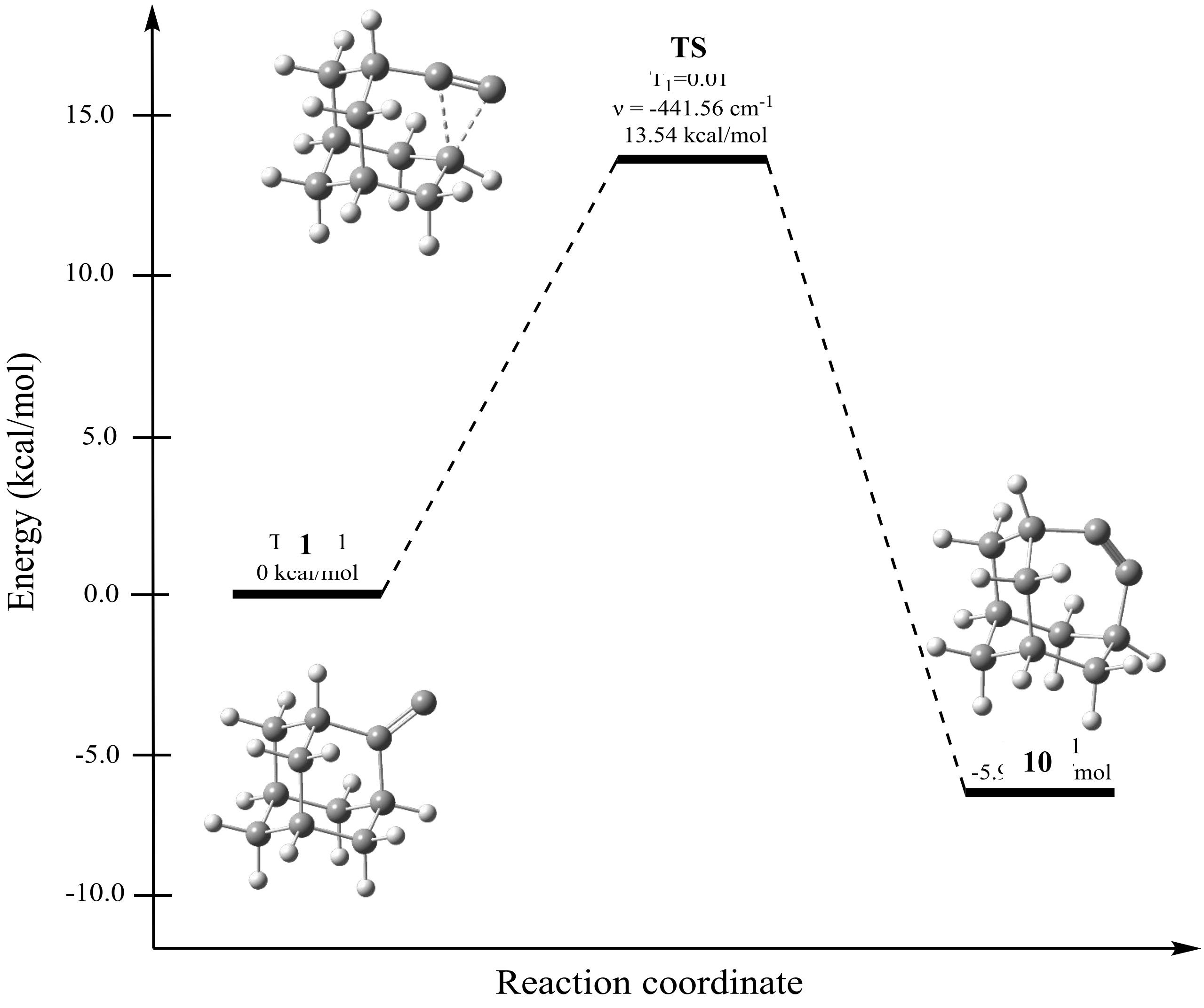

wb97x

Potential Energy Surface Diagram for DLPNO-CCSD(T)/def2-TZVP//ωB79x-D3BJ/def2-TZVP calculations:

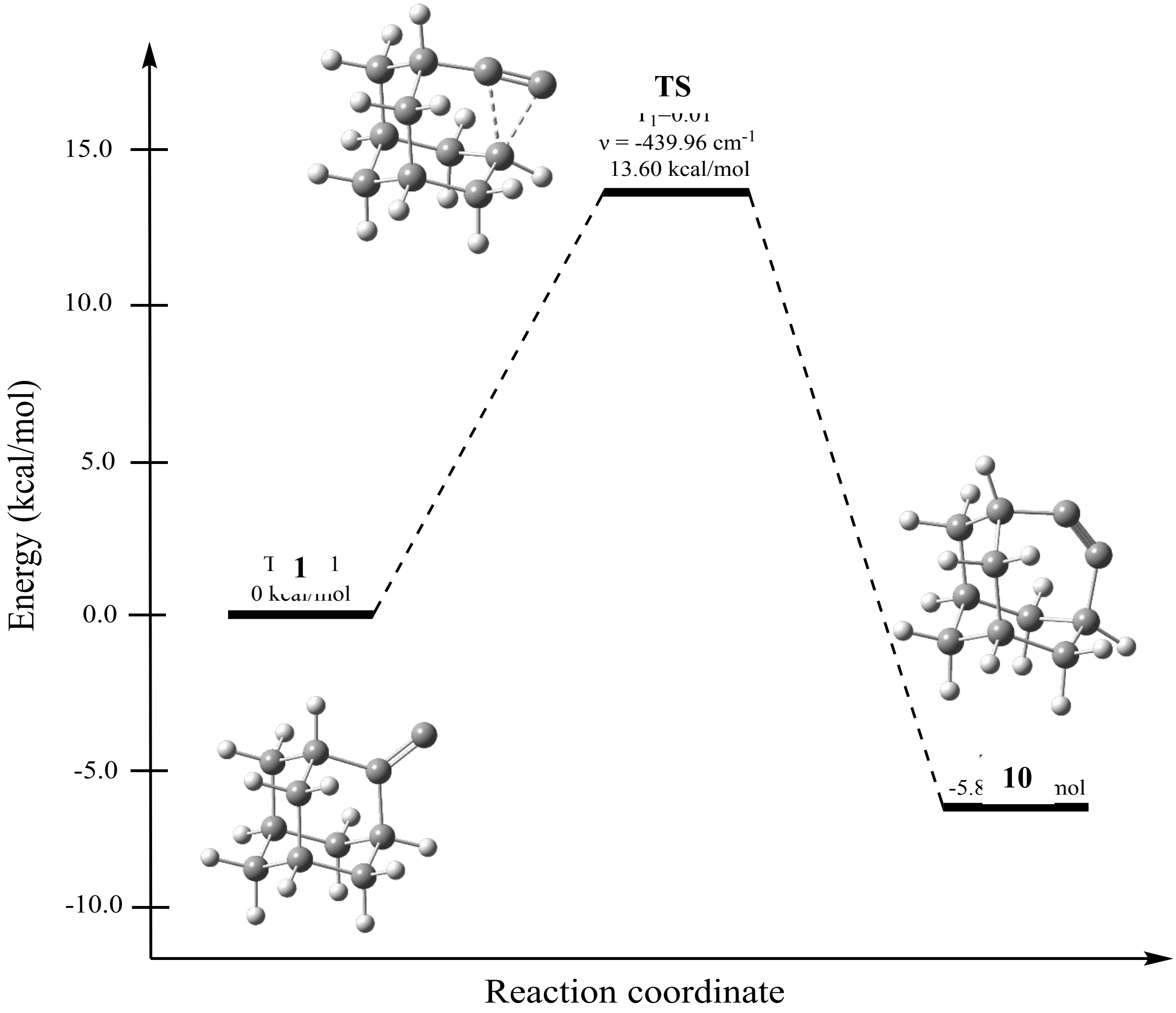

wb97x
